# Impact of substituents on molecular properties and catalytic activities of trinuclear Ru macrocycles in water oxidation†

**DOI:** 10.1039/d0sc01097a

**Published:** 2020-04-29

**Authors:** Ana-Lucia Meza-Chincha, Joachim O. Lindner, Dorothee Schindler, David Schmidt, Ana-Maria Krause, Merle I. S. Röhr, Roland Mitrić, Frank Würthner

**Affiliations:** Universität Würzburg, Institut für Organische Chemie Am Hubland 97074 Würzburg Germany wuerthner@uni-wuerzburg.de; Universität Würzburg, Center for Nanosystems Chemistry (CNC) Theodor-Boveri-Weg 97074 Würzburg Germany; Universität Würzburg, Institut für Physikalische und Theoretische Chemie Emil-Fischer-Str. 42 97074 Würzburg Germany

## Abstract

Herein we report a broad series of new trinuclear supramolecular Ru(bda) macrocycles bearing different substituents at the axial or equatorial ligands which enabled investigation of substituent effects on the catalytic activities in chemical and photocatalytic water oxidation. Our detailed investigations revealed that the activities of these functionalized macrocycles in water oxidation are significantly affected by the position at which the substituents were introduced. Interestingly, this effect could not be explained based on the redox properties of the catalysts since these are not markedly influenced by the functionalization of the ligands. Instead, detailed investigations by X-ray crystal structure analysis and theoretical simulations showed that conformational changes imparted by the substituents are responsible for the variation of catalytic activities of the Ru macrocycles. For the first time, macrocyclic structure of this class of water oxidation catalysts is unequivocally confirmed and experimental indication for a hydrogen-bonded water network present in the cavity of the macrocycles is provided by crystal structure analysis. We ascribe the high catalytic efficiency of our Ru(bda) macrocycles to cooperative proton abstractions facilitated by such a network of preorganized water molecules in their cavity, which is reminiscent of catalytic activities of enzymes at active sites.

## Abstract 2

Conformational changes induced by ligand substituents in macrocyclic Ru complexes strongly affect their chemical and photocatalytic efficiencies in water oxidation.

## Graphical abstract

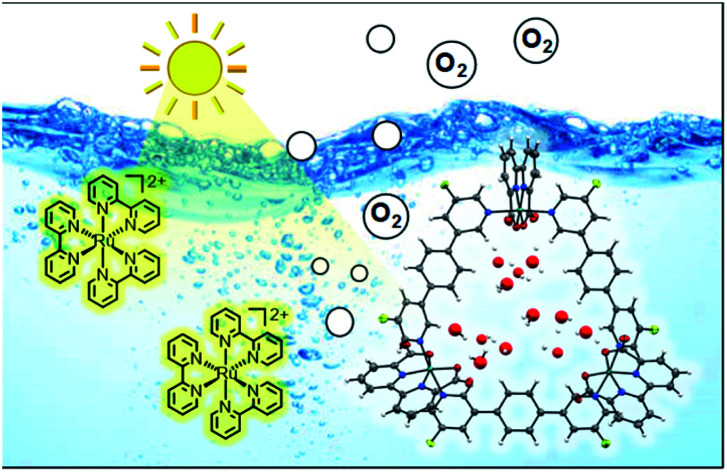

## Introduction

The development of clean and renewable energy sources that enable an efficient transition to a carbon-neutral economy constitutes one of the main challenges of our time.^1,2^ In this context, artificial photosynthesis offers a promising tool to conveniently store the energy of sunlight in chemical bonds of solar fuels such as hydrogen, methanol or other compounds generated by the reduction of CO_2_.^3–5^ As in natural photosynthesis, the required reducing equivalents are obtained from the oxidation of water to molecular oxygen in a very complex four-electron process.^6,7^ Accordingly, there is a need for efficient water oxidation catalysts (WOCs) that are able to perform this reaction at low overpotentials.^8,9^ Over the last decades, numerous homogeneous WOCs have been developed.^10–13^ Among those, ruthenium-based catalysts^12,13^ particularly Ru(bda) WOCs (bda: 2,2′-bipyridine-6,6′-dicarboxylic acid)^14,15^ have attracted much attention as some of these catalysts exhibit activities comparable to those of the oxygen-evolving complex of photosystem II.^16,17^

During the last decade, some insightful studies have been performed on the effect of substituents at axial^18–25^ and equatorial^26–28^ ligands on the catalytic performance of monomeric Ru(bda) WOCs. Hereby, uncomplicated manipulations of the axial ligands have enabled the synthesis of a plethora of catalysts within the family of mononuclear Ru(bda) WOCs. On the contrary, there are only sparse examples of modifications on the equatorial bda backbone presumably due to its higher synthetic complexity.^26–29^ These studies mainly focused on the electronic properties and non-covalent interactions of catalysts imparted by the substituents and how these affect the operating mechanism of water oxidation. However, the conformational effect imposed by ligand substituents on the accessibility of water molecules to the 7^th^ coordination site of Ru and thus on the catalytic activity of WOCs still remained unaddressed.

**Fig. 1:**
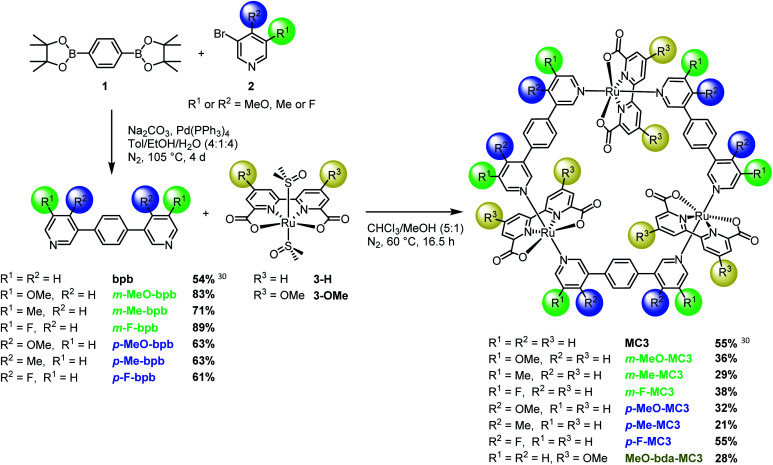
Synthesis of **MC3** macrocycles functionalized at the bda or bridging ligand in *meta* or *para*-position.

We have previously reported that the supramolecular ruthenium macrocycle **MC3** (Fig. 1) containing three catalytically active Ru(bda) units in a cyclic arrangement is a highly efficient catalyst for chemical water oxidation using ceric ammonium nitrate (CAN) as an oxidant under acidic conditions.^30^ Based on molecular dynamics simulations, we have recently proposed that the formation of a hydrogen-bonded network of preorganized water molecules inside the macrocyclic cavity might play a crucial role by promoting cooperative proton abstractions between the Ru centers. We hypothesized that such a defined water network might reduce activation barriers of proton-coupled reaction steps and, therefore, lead to the high catalytic activity of the catalyst.^31^ Kinetic studies as well as ^18^O labelling experiments have further shown that the supramolecular **MC3** catalyst operates by the WNA (water nucleophilic attack) mechanism in which a highly oxidized Ru^V^=O center is nucleophilically attacked by a water molecule to generate the O–O bond. In addition, the oxidation of Ru^IV^ to Ru^V^ was found to be the rate-determining step in this catalytic water oxidation.^30^ Thus, we envisioned that electron donating substituents either in the bridging or equatorial ligand might increase the electron density at the Ru centers and, as a result, decrease the Ru^V/IV^ oxidation potential and accelerate the rate of oxygen formation. Therefore, we have synthesized a series of new **MC3** derivatives that contain different substituents of varied electronic nature either at axial or equatorial ligands and thoroughly studied their catalytic activities in water oxidation.

Here we report the synthesis of a broad series of **MC3** derivatives that bear methoxy, methyl or fluoro substituents either in the bridging or equatorial bda ligand (Fig. 1 and S1†). The catalytic activities of these diversely substituted Ru macrocycles were studied in chemical water oxidation with Ce^IV^ as oxidant and under photochemical conditions using a three-component system based on a photosensitizer, a sacrificial electron acceptor and the supramolecular catalyst at a neutral pH. Our studies revealed that, although the redox properties of these catalysts are not significantly influenced by ligand substituents, the catalytic activities of these Ru macrocycles are strongly dependent on the position of the substituents. Based on crystallographic analysis and theoretical simulations, this unusual finding is explained by conformational effect imposed by the substituents in Ru macrocycles.

## Results

### Synthesis and structural characterization of functionalized Ru macrocycles

The new **MC3** derivatives bearing methoxy, methyl or fluoro groups in the *meta* or *para*-position of the bridging ligands were synthesized according to the route displayed in Fig. 1. The substituted bridging ligands **m-X-bpb** or **p-X-bpb** (X: MeO, Me or F) were readily accessible by Suzuki–Miyaura cross-coupling reactions (see ESI for details†). The macrocycles were then obtained by self-assembly of the ruthenium precursor [Ru(bda)(dmso)_2_] **3-H** and the respective bridging ligand in a chloroform/methanol mixture under a nitrogen atmosphere. Oligomeric side products as well as macrocycle fragments were removed by column chromatography over Al_2_O_3_ or SiO_2_ to yield the desired **MC3** derivatives in moderate to good yields for such supramolecular macrocyclization (21–55%). The synthetically more challenging **MeO-bda-MC3** macrocycle containing methoxy groups in the equatorial bda ligand was prepared in a four-step synthesis as depicted in Fig. S1.† Detailed synthetic procedures and characterization data of all new compounds are reported in the ESI.†

**Fig. 2:**
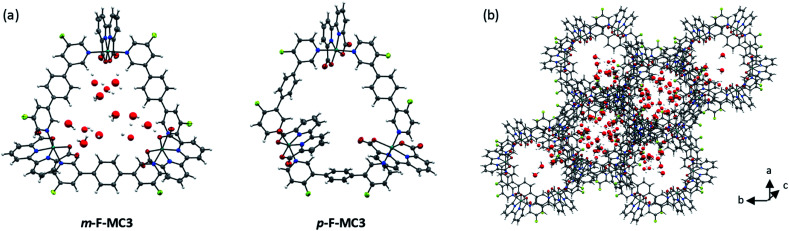
(a) Crystal structures of **m-F-MC3** and **p-F-MC3** determined by single crystal X-ray diffraction (ORTEP diagram with thermal ellipsoids set at 50% probability; grey: carbon, white: hydrogen, red: oxygen, blue: nitrogen, turquoise: ruthenium, green-yellow: fluorine). The crystals were grown from a DCM/methanol solvent (not dried) mixture. (b) Crystal packing of **m-F-MC3**, view along axis *c* with water and methanol molecules in the pores. *a* = 23.4927(5) Å, *b* = 23.4927(5) Å, *c* = 32.5166(7) Å, *α* = 90°, *β* = 90°, *γ* = 120°.

Single crystals of **m-F-MC3** and **p-F-MC3** suitable for X-ray analysis were obtained by slow evaporation of their respective solutions in not dried dichloromethane/methanol 5 : 1 mixture under argon atmosphere. The crystal structures of **m-F-MC3** and **p-F-MC3** unequivocally confirmed the formation of macrocyclic trimeric Ru complexes (Fig. 2). After refining the structure of **m-F-MC3** as a two-component twin, diffuse electron density originating from solvent molecules was located inside the macrocyclic cavity. Water molecules were assigned to the maxima of electron density, although the origin of these Q-peaks could not be elucidated unambiguously. However, the resulting hydrogen bonding network is in good agreement with the performed molecular dynamics simulations.^31^ We assume that the high hydrophilicity of the cavity constitutes the driving force for the accumulation of water molecules from not dried solvent in the macrocyclic cavity. The macrocyclic structure of the *para*-derivative **p-F-MC3** was refined as a two-component twin similarly to **m-F-MC3**. However, in this case residual electron density could not be modeled satisfactorily indicating the lack of ordered solvent molecules in the cavity and was therefore removed. Pleasingly, the remaining structure could be refined adequately.

Macrocycle **m-F-MC3** crystallizes in the trigonal space group *R*3̄ with a slipped stacked arrangement leading to the formation of one-dimensional pores (Fig. 2b). The distances between Ru center and the axially coordinated pyridyl rings are 2.081(5) Å and 2.084(4) Å. These values are comparable to those of the previously reported acyclic mononuclear complex [Ru(bda)(pic)_2_] (2.070(6) Å and 2.084(6) Å)^32^ and a larger macrocycle **MC4** (2.066(5) Å and 2.078(7) Å)^31^ that contains an additional phenyl ring in its bridging ligand. The distorted octahedrally coordinated Ru centers present an obtuse O–Ru–O angle of 122.8(2)°. Similarly to **MC4**, the axial pyridyl rings of **m-F-MC3** attached to one Ru center form a N_ax_–Ru–N_ax_ angle of 172.5(2)° and they are torsionally twisted by 48.7° (Fig. S2a†), thus leaving largely available Ru active sites for coordination of water molecules. Moreover, a torsion angle of 54.8° between the terminal pyridyl rings within one single bridging ligand is observed (Fig. S2b†). The intramolecular distance between two Ru centers is 12.057(9) Å and, more importantly, the open coordination sites of all Ru centers point to the interior of the macrocyclic cavity that should be favorable for the water oxidation process by preorganization of water molecules in the cavity.

Macrocycle **p-F-MC3** crystallizes in the triclinic *P*1̄ space group. The distances between the Ru centers and the axial pyridyl rings as well as the obtuse O–Ru–O and the N_ax_–Ru–N_ax_ angles are all very similar to those of the *meta*-substituted macrocycle **m-F-MC3** (Table S1†). In the crystal structure of **p-F-MC3** one Ru center with its open coordination site points to the exterior of the macrocyclic cavity. In strong contrast to **m-F-MC3**, the terminal pyridyl rings within the individual bridging ligands of **p-F-MC3** coordinated to the inverted Ru(bda) moiety show only a slight torsion of 2.0° and 5.3° (Fig. S2b†). Moreover, the axial pyridyl rings attached to two of the Ru centers are torsionally twisted by only 29.0° and 18.0° (Fig. S2a†). This results in restricted access of water molecules to the 7^th^ coordination site of these Ru centers.

### Redox and optical properties

Redox properties of Ru macrocycles were studied by cyclic voltammetry (CV) and differential pulse voltammetry (DPV) using 2,2,2-trifluoroethanol (TFE) as a non-coordinating co-solvent due to their poor solubility in pure water. The measurements were performed at pH 1 and pH 7 to resemble the conditions of the chemical and photocatalytic water oxidation experiments, respectively (see below). The redox properties observed in acidic and neutral media are summarized in Table 1 and Table S2,† respectively, and the voltammograms are displayed in Fig. S3–S17 (ESI†).

**Table 1:** Summary of redox properties of the macrocyclic **MC3** derivatives at pH 1^a^

Macrocycle	*E vs.* NHE [V]			
	Ru^III^_3_/Ru^II^_3_	Ru^III^_2_Ru^IV^/Ru^III^_3_	Ru^III^Ru^IV^_2_/Ru^III^_2_Ru^IV^	Ru^IV^_3_/Ru^III^Ru^IV^_2_
**MC3**	+0.71	+0.94	+1.17	+1.36
**m-MeO-MC3**	+0.73	—b	+1.17	+1.35
**m-F-MC3**	+0.82	—b	+1.17	+1.40
**m-Me-MC3**	+0.70	—b	+1.17	+1.35
**p-F-MC3**	+0.75	—b	+1.17	+1.39
**p-MeO-MC3**	+0.65	+0.94	+1.17	+1.36
**p-Me-MC3**	+0.67	+0.94	+1.17	+1.35
**MeO-bda-MC3**	+0.60	+0.87	+1.14	+1.35

a CV and DPV in TFE/H_2_O 1 : 1 (pH 1, triflic acid), *c* = 0.25 mM.

b Not detectable due to low intensity and overlap with neighboring waves.

Under acidic conditions, four reversible oxidation processes were detected which correspond to an initial three-electron Ru^III^_3_/Ru^II^_3_ oxidation followed by three subsequent one-electron oxidation events leading to the formation of a Ru^IV^_3_ state (Fig. S3–S9†). The final oxidation to Ru^V^ could not be observed, presumably due to overlap with the water oxidation current. The differences in peak current intensity observed in CV and DPV for the single redox processes (Fig. S3–S9†) might be explained based on the number of transferred electrons and the kinetic hindrance resulting from proton coupling to the electron transfer processes. These made it challenging to assign the Ru^III^_2_Ru^IV^/Ru^III^Ru^IV^_2_ oxidation potential for all macrocycles, albeit the electrochemical trend is clear. Contrary to our expectations, the introduction of substituents to the bridging ligand affected the Ru^III^_3_/Ru^II^_3_ redox potential but had a negligible impact on the higher oxidations. The methoxy groups on the bda ligand of **MeO-bda-MC3** had a more pronounced effect on the redox properties of the macrocycle, however, only up to the Ru^III^Ru^IV^_2_/Ru^III^_2_Ru^IV^ oxidation.

Under neutral conditions (pH 7), three three-electron oxidation events were observed for each of the **MC3** derivatives (Fig. S10–S17†). These were assigned to the reversible electron redox couples Ru^III^_3_/Ru^II^_3_, Ru^IV^_3_/Ru^III^_3_ and Ru^V^_3_/Ru^IV^_3_ in accordance to the measured Pourbaix diagram (Fig. S18†). At this pH, the introduction of substituents again mainly affected the initial Ru^III^_3_/Ru^II^_3_ oxidation (Table S2†). This is in agreement with previous reports by Llobet^21,33^ and Sun^15^ and co-workers. These authors independently stated that, although it is possible to affect the Ru^III/II^ potential of monomeric Ru(bda) WOCs *via* the introduction of substituents to the axial ligand, the lower participation of these ligands in the HOMO/LUMO at higher oxidation states of Ru leads to a limited effect on the Ru^IV/III^ and Ru^V/IV^ oxidations. Surprisingly, functionalization of the bda moiety, which was expected to have a more prominent effect on the redox properties of the macrocycle, led to similar results.

UV/Vis absorption spectroscopy of the **MC3** macrocycles at the Ru^II^_3_ state revealed that the substituents at bridging ligand have negligible effect on the optical properties of the WOCs under acidic and neutral conditions (Fig. S20†). Under both conditions, the strong band at around 300 nm corresponds to the π–π* ligand-centered transitions whereas the broad bands between 350 nm and 550 nm can be assigned to metal-to-ligand charge transfer (MLCT) processes.^18,34^ According to theoretical calculations,^31^ the higher energy MLCT band at around 350 nm can be assigned to the transition from the Ru d-orbital to the π*-orbital of the axial ligand. The less energetic bands between 450 nm and 550 nm are characteristic for the transition from the Ru d-orbital to the π*-orbital of the bda ligand. As it can be seen in Fig. S20,† the absorption spectra of all macrocycles are very similar with only slight differences in the position of the higher energetic MLCT band as expected after functionalization of the axial ligands. Additionally, spectroelectrochemistry was performed to analyze the absorption spectra of the oxidized macrocycles upon increasing the set potential from 500 mV to approximately 750 mV and then to 1000 mV (Fig. S21†). Here again, only modest shifts of the characteristic bands at 700 nm and 550 nm for the Ru^III^_3_ and Ru^IV^_3_ states, respectively, were observed. Detailed spectroelectrochemistry data of the **MC3** derivatives are presented in Fig. S22–S25 (ESI†).

### Catalytic water oxidation with chemical oxidant in acidic aqueous solutions

Catalytic activities of the functionalized **MC3** macrocycles toward water oxidation were first explored by using ceric ammonium nitrate (CAN) as a strong one-electron oxidant. Hereby, a solution of the respective macrocycle was injected into a sealed Schlenk vial containing a fresh solution of CAN in acetonitrile (MeCN)/water mixtures (pH 1, triflic acid). Acetonitrile was used as an organic co-solvent due to its known robustness under oxidative conditions.^19,31,35^ The amount of evolved oxygen was determined by the increase of pressure in the vials after catalyst injection as detected by attached pressure sensors. Further, gas composition of the headspace in each reaction vial at the end of catalysis was evaluated by gas chromatography (GC).

Initially, chemical water oxidation experiments with the **MC3** derivatives as catalysts were performed in variable amounts of acetonitrile (Table S3†). This organic solvent, though necessary to achieve solubility of the macrocycles in aqueous mixtures, is known for its ability to compete with water for the binding sites of Ru(bda) WOCs and thus reduce their catalytic performance.^19^ Therefore, prior to conducting detailed concentration-dependent experiments the optimal solvent composition for each **MC3** macrocycle was determined. Interestingly, while the highest catalytic activity of the unsubstituted **MC3** compound is reached in aqueous mixtures containing 60% of MeCN,^30^ the functionalized macrocycles showed their best performance at 50%. Accordingly, all further experiments were carried out in MeCN/H_2_O 1 : 1 solvent mixture.

**Fig. 3:**
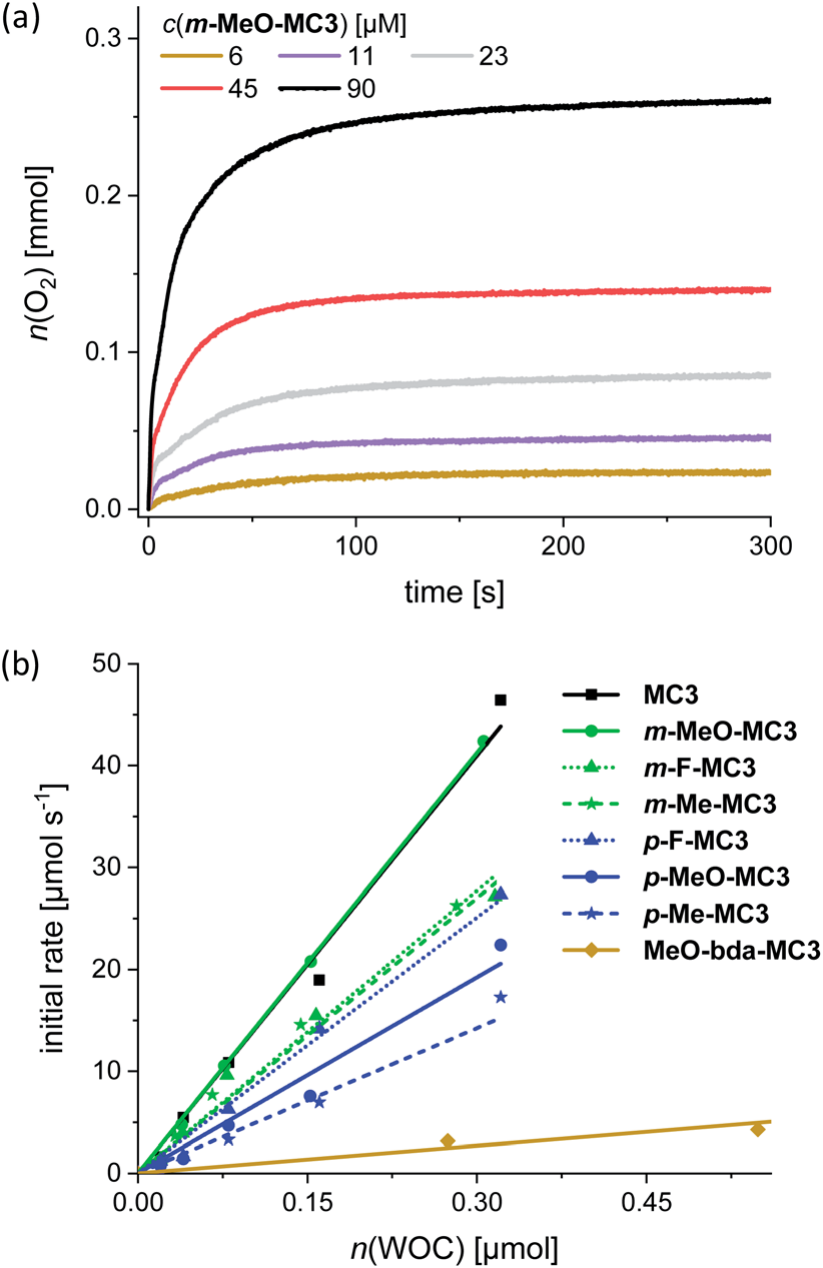
(a) Oxygen evolution curves of **m-MeO-MC3** at variable concentrations in MeCN/H_2_O 1 : 1 (pH 1, triflic acid), *c*(CAN) = 0.6 M. (b) Concentration-dependent initial rates of all macrocycles including linear regression for the determination of averaged TOF.

Water oxidation at varying WOC concentrations was performed for accurate determination of TOF and TON values for each of the Ru macrocycles, including the parent compound **MC3** as a reference. As exemplarily depicted in Fig. 3a for **m-MeO-MC3** (see Fig. S26–S33† for other compounds), the amount of evolved oxygen is clearly dependent on the concentration of the catalyst. Further, a linear relationship between the catalyst amount and the initial rates of catalysis was observed (Fig. 3b). The initial rates were determined in the first two seconds after injection of the WOC and the observed linear relationship complies with the first order kinetics of the previously proposed WNA mechanism for the unsubstituted **MC3** macrocycle.^30^ The calculated linear regressions in Fig. 3b represent the averaged TOF value of the respective **MC3** derivative. Additionally, a TON was calculated for each concentration and the highest TON is reported in Table 2.

**Table 2:** Catalytic activity of **MC3** derivatives in chemical water oxidation^a^

Macrocycle	TOF [s^−1^]	TON
**MC3**	136 ± 2	5300 ± 800
**m-MeO-MC3**	138 ± 1	1300 ± 100
**m-F-MC3**	92 ± 6	4700 ± 800
**m-Me-MC3**	90 ± 4	3700 ± 500
**p-F-MC3**	84 ± 4	2500 ± 400
**p-MeO-MC3**	64 ± 5	2200 ± 400
**p-Me-MC3**	47 ± 6	2300 ± 450
**MeO-bda-MC3**	10 ± 1	1000 ± 150

a Experiments were performed in MeCN/H_2_O 1 : 1 (pH 1, triflic acid); *c*(CAN) = 0.6 M, *c*(WOC) = 5–322 μM.

The data in Table 2 revealed that the introduction of substituents in either the bridging or equatorial bda ligand did not result in an improvement of the catalytic activities of the functionalized macrocycles in chemical water oxidation compared to the unsubstituted **MC3** WOC (TOF = 136 s^−1^, TON = 5300). This is in line with the observed redox properties of the macrocycles in acidic medium (see Table 1). However, there are some distinct differences with regard to the catalytic performance of the **MC3** derivatives that cannot be explained based on the redox properties of the catalysts. For instance, independent of the introduced substituents the *meta*-substituted macrocycles reached in all cases higher TOF values than their *para*-substituted counterparts. This regioisomer effect on catalytic activity is more pronounced for the methoxy and methyl-substituted macrocycles as the TOF values of the *para*-derivatives are halved compared to those of the respective *meta*-**MC3** macrocycles. Further, functionalization of the bda ligand with methoxy groups led to a decrease in catalytic efficiency of more than one order of magnitude compared to parent compound **MC3** (see Table 2). Control experiments have shown that Ru precursors RuCl_2_(dmso)_4_ and Ru(bda)(dmso)_2_ do not exhibit any significant catalytic activities for water oxidation under identical experimental conditions (Fig. S51a†).

The reduction in TON of the **MC3** derivatives might be attributed to the diminished stability of the functionalized ligands under strong oxidative conditions. As it was demonstrated by CV and DPV (Fig. S19†), the modified bridging ligands indeed undergo an irreversible oxidation whereas the unsubstituted **bpb** ligand remains intact within the same electrochemical window. The lowest TON (reflects the stability of the catalyst) values obtained for the methoxy-functionalized macrocycles compared to the methyl and fluoro **MC3** derivatives can be attributed to a higher lability of the methoxy bearing ligands in acidic media. However, there is no apparent correlation between the TOFs and TONs of the functionalized macrocycles as **m-MeO-MC3** with the second lowest TON of 1300 reached the highest TOF value in the series with 138 s^−1^. GC analysis of each reaction headspace confirmed that oxygen was the only gaseous product formed during catalysis (Fig. S34–S41†). Thus, we assume that catalyst deactivation involves to some extent oxidative decomposition of the ligands without the release of CO or CO_2_ in addition to the disassembly of the macrocycles by loss of the axial ligands, which is usual deactivation pathway for Ru(bda) WOCs^17^ and our previously reported Ru macrocycles.^30,31,36^

### Photocatalytic water oxidation under neutral conditions

**Fig. 4:**
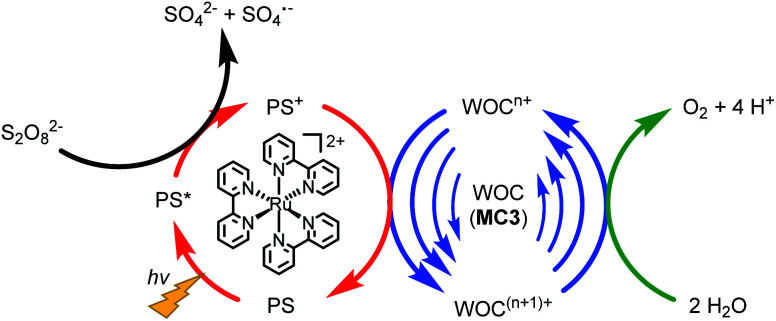
Illustration of the catalytic cycle of photocatalytic water oxidation using Na_2_S_2_O_8_ as sacrificial electron acceptor, [Ru(bpy)_3_]^2+^ as PS and **MC3** derivatives as WOC.

The catalytic reactivity of the **MC3** macrocycles was further investigated in photocatalytic water oxidation using a three-component system based on ruthenium tris(bipyridine) as a photosensitizer (PS), sodium persulfate as sacrificial electron acceptor and the **MC3** derivatives as WOC (a general scheme is shown in Fig. 4). In these experiments, the mild oxidant [Ru(bpy)_3_]^3+^ is photogenerated *in situ* by oxidation of the excited PS* in ^3^MLCT state by the sacrificial electron acceptor.^37,38^ Upon one-electron reduction, the persulfate ion splits into a SO_4_^2−^ anion and a SO_4_˙^−^ radical. The latter is able to oxidize a second PS molecule or even the WOC.^39^ Subsequently, the PS is regenerated through one-electron oxidation of the WOC by PS^+^.^39^ This process is repeated until the catalyst reaches the Ru^V^ oxidation state which finally oxidizes water to molecular oxygen. We note the higher complexity of our system for which due to the presence of three Ru centers com- or disproportionations may play a role.

**Fig. 5:**
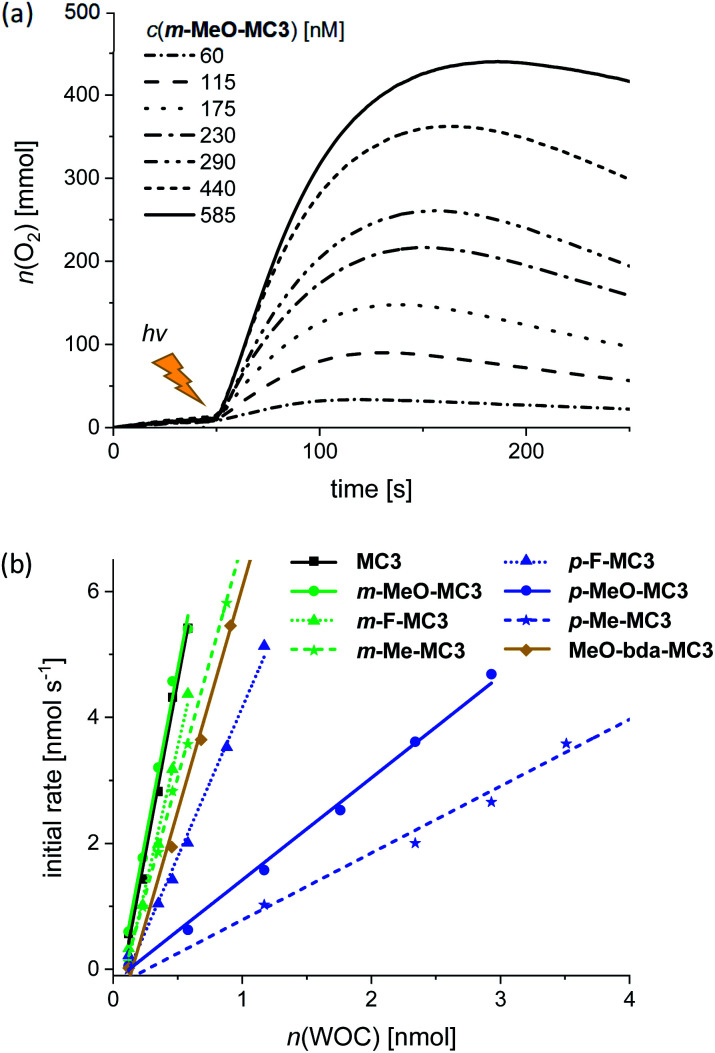
(a) Concentration-dependent oxygen evolution curves of **m-MeO-MC3** in MeCN/H_2_O 1 : 1 (pH 7, phosphate buffer), *c*(PS) = 1.5 mM, *c*(Na_2_S_2_O_8_) = 37 mM. The lighting symbol indicates the start of sample irradiation at *t* = 50 s. (b) Initial rates of all macrocycles with linear regression for the determination of averaged TOF.

Photocatalytic water oxidation was performed in phosphate buffer at pH 7, using again acetonitrile as an oxidatively stable co-solvent to circumvent the poor water solubility of the macrocycles. Hereby, a 1 : 1 ratio of MeCN/H_2_O was used as for chemical water oxidation. Samples were irradiated using a xenon lamp carefully calibrated to an irradiation power of 100 mW cm^−2^ (Fig. S42†) and the generated oxygen was detected with a Clark electrode set-up. The catalytic activities of the **MC3** derivatives were studied under identical conditions to enable a reliable comparison of the results. Accordingly, in all experiments a large excess of PS (up to 2.5 × 10^3^ equiv.) and Na_2_S_2_O_8_ (6.0 × 10^5^ equiv.) was used. Both components were dissolved in the dark and mixed with the respective **MC3** macrocycle directly in the experiment chamber, which was kept in the dark at 20 °C for 50 s prior to irradiation. As for chemical water oxidation, the amount of evolved oxygen was measured at variable WOC concentrations. Fig. 5a shows the oxygen evolution curves of **m-MeO-MC3** as a representative example (see Fig. S43–S50† for other catalysts). Note that after reaching a plateau of maximal O_2_ concentration, some of the dissolved oxygen is released into the gas phase leading to a reduction in the amount of gas detected by the Clark electrode. As in chemical water oxidation, both the amount of oxygen and the initial rates of oxygen generation in photocatalytic oxidation are dependent on the concentration of the WOC. The initial rates were determined from the linear part of the curves at the beginning of catalysis between 55–70 s. An averaged TOF value for each macrocycle was then obtained from a linear regression in the plot of the initial rates *vs.* the amount of catalyst (Fig. 5b). The TON was calculated from the maximum amount of oxygen produced by each WOC during the experiments. As before, the reported TON is the highest value obtained for each macrocycle (Table 3).

**Table 3:** Catalytic activity of **MC3** derivatives in photochemical water oxidation^a^

Macrocycle	TOF [s^−1^]	TON
**MC3** b	10.9 ± 0.5	430 ± 20
**m-MeO-MC3**	10.8 ± 0.6	480 ± 20
**m-F-MC3**	9.1 ± 0.7	400 ± 10
**m-Me-MC3**	7.8 ± 0.3	380 ± 20
**p-F-MC3**	4.8 ± 0.3	260 ± 20
**p-MeO-MC3**	2.0 ± 0.1	170 ± 20
**p-Me-MC3**	1.7 ± 0.2	120 ± 10
**MeO-bda-MC3**	7.8 ± 0.1	120 ± 20

a Photochemical water oxidation in MeCN : H_2_O 1 : 1 (pH 7, phosphate buffer), *c*(PS) = 1.5 mM, *c*(Na_2_S_2_O_8_) = 37 mM, *c*(WOC) = 60–600 nM.

b In a previous publication (ref. 30) different catalytic values were reported for **MC3** (TOF > 13.1 s^−1^, TON > 1255). This discrepancy might be attributed to contamination of the Clark electrode or not properly accounted intensity fluctuations of the light source in our previous work.

Under photocatalytic conditions a similar trend was observed for the catalytic activities of the functionalized **MC3** macrocycles as for chemical water oxidation, with the exception that **MeO-bda-MC3** exhibits here a comparatively high TOF of about 8 s^−1^ while in chemical catalysis this WOC exhibited the lowest activity in the series. Otherwise, independent of the nature of the substituents at the bridging ligand, the *meta*-substituted derivatives showed similar TOFs as the parent **MC3** macrocycle (TOF = 11 s^−1^) and also reached in all cases higher values than the respective *para*-**MC3** WOCs (Table 3). It is noteworthy that the difference in TOF comprises nearly one order of magnitude in the most extreme case of the methoxy **MC3** derivatives (TOF**m-MeO-MC3** = 11 s^−1^, TOF**p-MeO-MC3** = 2 s^−1^). It should be noted that the photocatalytic TOFs are distinctly lower than those measured under chemical conditions. This can be attributed to the limited stability of the PS and to the general complexity inherent to the applied three-component system as it has been extensively discussed in the literature.^40–42^ However, it should be emphasized that the functionalized macrocycles exhibit higher photocatalytic activities in water oxidation than most of the so far reported homogeneous Ru catalysts, including Ru(bda) WOCs that do not reach TOFs above 1 s^−1^ (ref. 43) (see Table S4 in ESI† for comparison of TOF and TON values of selected Ru WOCs). Control experiments with Ru precursors revealed that the measured catalytic activities are originated from trinuclear Ru(bda) catalysts (Fig. S51b†). Under photocatalytic conditions the *meta*-substituted macrocycles exhibit similar TON values as the parent compound **MC3** (TON ≥ 380), whereas the *para*-derivatives show significantly lower TONs compared to the reference and their *meta* congeners as well. Our results clearly indicate that the catalytic activities in terms of TOFs could be influenced by the stabilities of the functionalized macrocycles under photocatalytic conditions which are reflected in the obtained TON values. However, **MeO-bda-MC3** with the lowest TON of the series (120) exhibits a rather high catalytic activity reaching a TOF of 7.8 s^−1^. In both chemical and photocatalytic water oxidation experiments, **MeO-bda-MC3** was least stable which is in agreement with a previous report by Liu and co-workers where a drastic decrease in TON was also observed by functionalization of the bda ligand of Ru WOCs at the same position.^27^

## Discussion

**Fig. 6:**
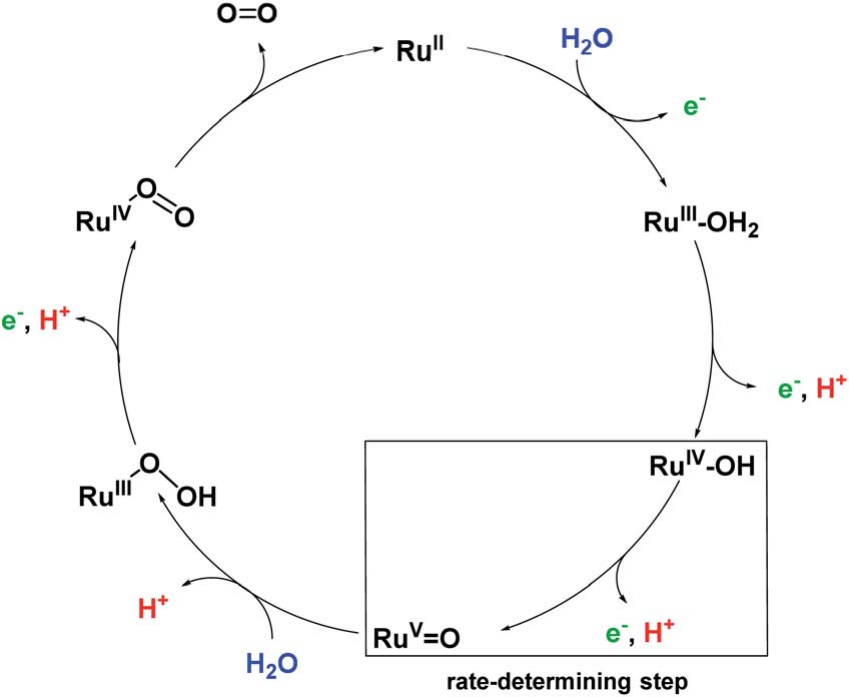
Simplified proposal for WNA mechanism of water oxidation for **MC3** macrocycle.^30^ Presumably, after liberation of O_2_ the Ru^II^ species is instantaneously oxidized by neighboring Ru^IV^ centers by comproportionation.

Our detailed studies on chemical and photocatalytic water oxidation with a series of Ru(bda) macrocycles revealed that the catalytic activities are strongly influenced by the substituents at axial or equatorial ligands. However, the introduction of methoxy, methyl or fluoro substituents did not achieve the expected modification of the redox properties of the macrocycles neither at pH 1 nor pH 7 (see Tables 1 and S2†). Moreover, a linear relationship between the catalyst amount and the initial rates of catalysis was observed for all **MC3** macrocycles in both chemical and photocatalytic water oxidation which complies with the first order kinetics of the previously proposed WNA mechanism (Fig. 6) for the unsubstituted **MC3** macrocycle, facilitated by network of hydrogen-bonded water molecules in the cavity.^30,31^ Thus, the observed trends in catalytic performance revealing a higher catalytic activity of all *meta*-substituted macrocycles compared to their *para*-substituted counterparts can neither be ascribed to a modification of the electronic properties of the macrocycles nor to a change in operating mechanism of water oxidation. Based on these results, we consider that conformational changes imparted by the ligand substituents as the reason for diverse catalytic activities of the Ru macrocycles. Therefore, theoretical calculations were performed to substantiate this notion that are discussed in the following.

### Characterization of the water network inside the cavity

**Fig. 7:**
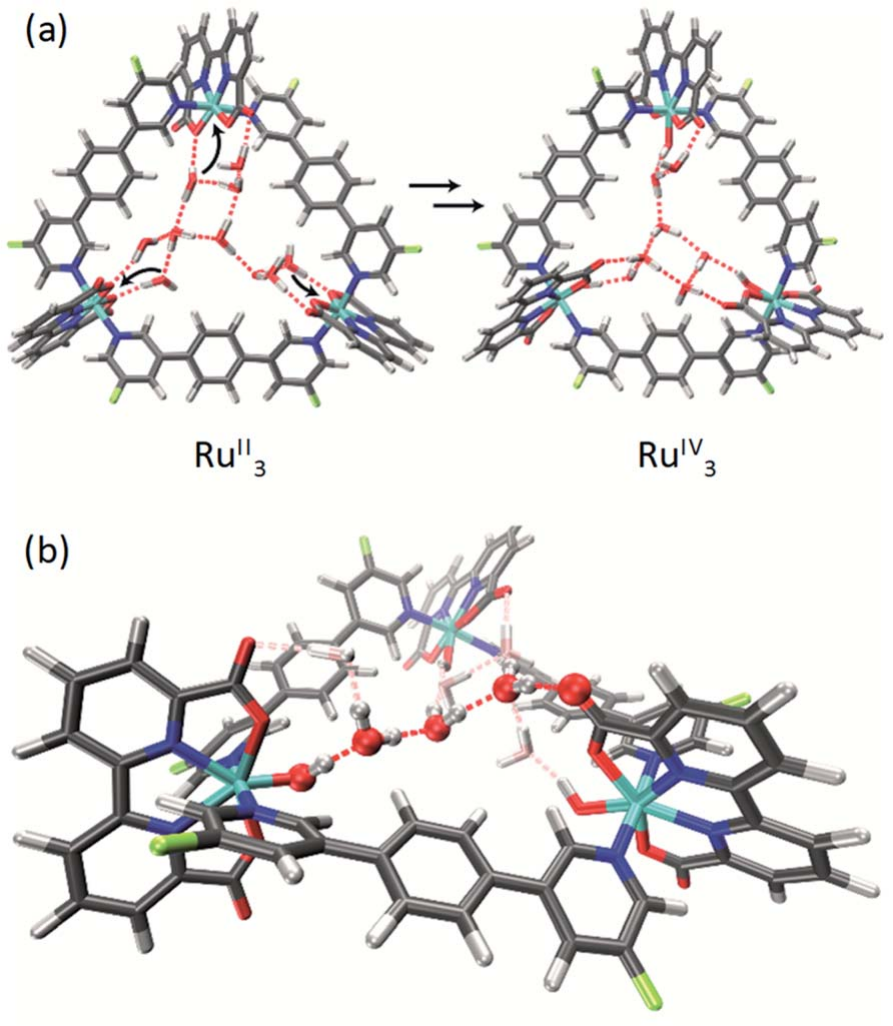
(a) DFT-refined structure of the water network inside the cavity of **m-F-MC3** in the Ru^II^_3_ and Ru^IV^_3_ oxidation states derived from the crystal structure depicted in Fig. 2. The coordination of water molecules needed to form higher oxidation states is indicated by black arrows. (b) Zoomed perspective view on the three water molecules connecting two catalytic centers optimized in the Ru^IV^_3_ state. The other water molecules are grayed out for clarity.

For the unsubstituted **MC3**, our molecular dynamics simulations revealed that carboxy groups of the bda ligands can act as proton relays through a network of hydrogen-bonded water molecules. This facilitates proton-coupled electron transfer (PCET)^44^ processes during catalysis, such as the rate-determining oxidation of the Ru^IV^ species of the WOC (Fig. 6).^31^ Interestingly, the crystal structure of **m-F-MC3** provided the first experimental indication for a hydrogen-bonded water network in the inner cavity of trinuclear macrocycles (see Fig. 2a). We assume that this is only possible when the hydrophilic carboxy groups of all Ru(bda) moieties are rotated into the inside of the cavity, since no highly organized water network was found in the crystal structure of **p-F-MC3** where most of the carboxy groups of Ru(bda) are directed outwards. Since the positions of hydrogen atoms are not available from the crystal structure, in order to investigate the intermolecular interactions in the water network, the **m-F-MC3** structure obtained in the solid state was refined on the DFT level including additional implicit solvation (see ESI for details†). For this purpose, nine water molecules with shortest distance to Ru were selected and an additional water molecule was placed in the center of the macrocycle where some diffuse electron density could not be assigned unambiguously in the XRD data. The resulting locally optimized structure depicted in Fig. 7a shows that the three catalytic sites are connected by a network of nicely ordered water molecules with H-bonding distances below 1.9 Å. This clearly indicates that the water molecules, as located in the crystal structure, are appropriately arranged to form strong networks.

This also holds true upon oxidation of the macrocycle to the Ru^IV^_3_ oxidation state. A structure of **m-F-MC3** in this oxidation state was obtained by coordination of one water molecule to each Ru center accompanied by removal of three protons and re-optimization. The resulting [Ru^IV^–OH]_3_ species features a hydrogen-bonded water network that connects the hydroxo protons of each Ru^IV^ center with the carboxy groups of the remaining Ru(bda) moieties by only three water molecules. Stabilization of oxidized WOC intermediates by hydrogen bonds was also observed for other Ru catalysts such as Ru^IV^=O(damp)(bpy) (damp: bis((dimethylamino)methyl)pyridine)^45^ and recently for a Cu(ii)-based WOC in photocatalytic water oxidation.^46^ As illustrated in Fig. 7b, preorganization of water molecules in the cavity of our macrocyclic WOCs presumably allows for efficient proton transfer following a Grotthuss-type mechanism.^47^ Therefore, our studies suggest that proton abstraction in the rate-determining oxidation of Ru^IV^ to Ru^V^ (Fig. 6) could be facilitated if the macrocycles adopt in solution a conformation close to that found in the crystal structure of **m-F-MC3** which promotes the formation of a highly organized water network. Remarkably, in nature the function of several enzymes as well as of the oxygen-evolving complex Mn_4_CaO_5_ of photosystem II also relies on such preorganized water networks to facilitate PCET processes and accelerate the rates of catalysis.^48–50^

### Conformational effects of axial ligand substituents

**Fig. 8:**
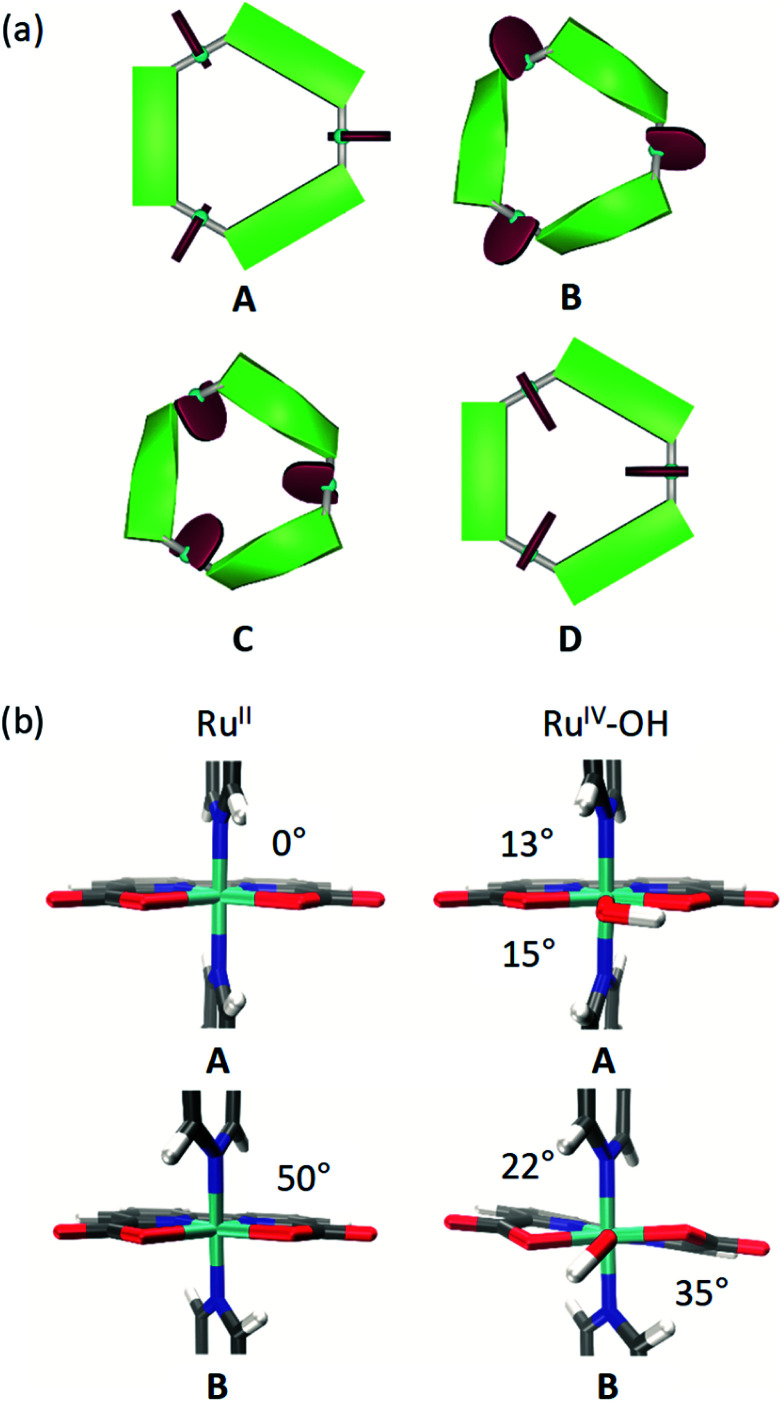
(a) Schematic illustration of conformational equilibrium of **MC3** derivatives in solution displaying four possible conformations **A–D**; turquoise: ruthenium, red: bda ligands, green: bridging ligands. (b) Study of C–N_ax_–N_ax_–C (left) and C–N_ax_–Ru–O (right) torsion angles between the axial ligands as well as axial and hydroxo ligand in DFT optimized structures of **MC3** in the Ru^II^_3_ or Ru^IV^_3_ oxidation states, respectively.

Several different conformations of the newly designed Ru macrocycles are likely to be present in solution as a result of twisting of the bridging ligands or by rotation of the Ru(bda) units. Representative examples of symmetric conformers bearing either “flat” or “twisted” bridging ligands and Ru(bda) moieties with the open coordination site pointing to the in- or outside of the macrocyclic cavity are depicted in Fig. 8a. Structures of mixed character between these extremes are omitted for simplicity. To gain insight into these conformations of the macrocycles in solution, which might provide some rational for the reserved superior catalytic activities of *meta*-substituted derivatives compared to *para*-macrocycles, theoretical studies were performed on the basis of semi-empirical calculations (see ESI for details†). For this purpose, the methylated **MC3** derivatives **m-Me-MC3** and **p-Me-MC3** at the Ru^II^_3_ oxidation state were chosen as model compounds due to their particularly distinct catalytic activities in higher oxidation states (see Tables 2 and 3). We assume that the steric influence of the substituents on the macrocyclic structure should be independent of the oxidation state of Ru. Calculations on both methylated **MC3** macrocycles showed that structures bearing three Ru(bda) moieties with the open coordination sites rotated to the outside of the cavity (*cf.* structures **C** and **D** in Fig. 8a) are very unlikely (Table S5†). More interestingly, the most stable conformation of **m-Me-MC3** and reference compound **MC3** was found to be the structure **B** with calculated energy of −22.5 kJ mol^−1^ relative to **A**. Notably, this is also the solid-state conformation of **m-F-MC3**. In the case of **p-Me-MC3**, the structures **A** and **B** are nearly isoenergetic. Metadynamics simulations in the conformational space of the bridging ligands (see ESI for details†) revealed that functionalization at the *para*-position indeed induces a more drastic twisting of these ligands with a concomitant raise of macrocyclic ring strain in **B**. As a result of the destabilization of **B**, **A** and **B** conformers of the *para*-macrocycles are likely to be present in similar concentrations in solution. In contrast, the *meta*-derivatives most probably exist in the **B** conformation as this conformation is energetically favored compared to **A** (Table S5†). The significance of this conformational effect on catalytic activity will be discussed in the following.

To assess the rotation of the Ru(bda) units around the N_ax_–Ru–N_ax_ bond in solution additional metadynamics simulations were conducted. The resulting free energy profiles (see Fig. S53 and S54†) show that in structure **A** rotation of one of the bda ligands to the inside of the cavity requires 11 kJ mol^−1^, while 22 kJ mol^−1^ are needed in the case of **B**. Thus, it can be concluded that conformations similar to the solid state structure of **p-F-MC3** with one open coordination site of Ru pointing to the exterior of the cavity could indeed be found in solutions of the *para*-substituted macrocycles as these exist in an equilibrium between the **A** and **B** structures. In the case of *meta*-WOCs, such a rotation of the Ru(bda) units is rather unlikely since these macrocycles mostly adapt the **B** conformation in solution which does not allow for such a rotation. However, it should be mentioned that conformers of the *para*-macrocycles bearing rotated Ru(bda) moieties which would not support an inner hydrogen-bonded water network represent most probably only a minor fraction in solution. Our studies indicate that such structures exist in equilibrium with more abundant structures containing Ru(bda) units with the open coordination sites oriented to the inside of the macrocyclic cavity.

DFT optimizations (see ESI for details†) of both **A** and **B** conformations of **MC3** were performed to get a more accurate insight into the direct ligand environment around Ru, which presumably defines the accessibility of the catalytic centers for water molecules. As depicted in Fig. 8b, **A** exhibits a C–N_ax_–N_ax_–C torsion of 0°, whereas an opening angle of 50° is observed in **B**. Further, in the Ru^IV^_3_ oxidation state where a hydroxo ligand is attached to Ru on the equatorial plane, the C–N_ax_–Ru–O torsion angles are distinctly larger in **B** than in **A** which is reflected in a stabilization energy of 35 kJ mol^−1^ of **B** over **A** (Table S6†). Therefore, we conclude that the conformation **B** of the Ru macrocycles plays a crucial role in their catalytic efficiency since the critical access of water molecules to the 7^th^ coordination site of Ru(bda) WOCs in only possible in this conformation. Accordingly, the *meta*-substituted **MC3** macrocycles which are predominantly present in **B**-type structures in solution show higher catalytic reactivities than the *para*-derivatives. For the latter, the observed lower catalytic activities in both chemical and photocatalytic water oxidation might be due to the presence of an equilibrium between unfavorable and favorable conformations as predicted by our calculations. In unfavorable conformations, the access of water molecules to the 7^th^ coordination site of the Ru centers of the *para*-macrocycles is restricted, thus resulting in a reduced efficiency for water oxidation catalysis by a WNA mechanism.

## Conclusions

A series of Ru(bda) macrocycles bearing different substituents at both axial and equatorial ligands were synthesized and their molecular properties were studied. More importantly, we have explored here the catalytic activities of the newly synthesized series of Ru macrocycles (**MC3** derivatives) in chemical and photocatalytic water oxidation to gain insight into the electronic and steric impact of the substituents on their catalytic efficiencies. For the first time, we have obtained crystallographic evidence for the **MC3** macrocyclic structure. Interestingly, one of the crystal structures provided the first experimental indication for a hydrogen-bonded water network in the macrocyclic cavity that was considered to be essential for the high catalytic efficiency for a WNA mechanism according to theoretical calculations reported previously.^30,31^ This strengthens our hypothesis that the high catalytic activity of the **MC3** macrocycles is closely related to the presence of such a network of preorganized water molecules which induce efficient cooperative proton abstraction processes during water oxidation.

Contrary to our expectations, neither the modification of the axial nor the bda ligands resulted in the desired tuning of the oxidation potentials of the Ru macrocycles to modulate the formation of catalytically active Ru^V^ oxidation state. However, our studies clearly revealed that the catalytic performance of the **MC3** macrocycles is dependent on the position at which the substituents are introduced. Thus, the macrocyclic nature of our catalysts provided the unique opportunity to perform to date unprecedented studies on the role of steric effects on the catalytic activity of regioisomeric *meta*- and *para*-substituted Ru(bda) WOCs. Accordingly, our detailed investigation by X-ray crystal structure analysis as well as metadynamics and DFT simulations has convincingly shown that functionalization of the macrocycles at the *para*-position induces conformational changes with partial rotation of one of the Ru(bda) active centers to the outside of the macrocyclic cavity. The reduced catalytic performance of these *para*-WOCs in both chemical and photochemical water oxidation cannot be ascribed to this rotation as we could demonstrate that it only plays a minor role in solution. Instead, we propose that the more restricted access of water molecules to the 7^th^ coordination site of Ru of these *para*-macrocycles resulting from parallel orientation of the axial pyridyl ligands leads to their diminished water oxidation efficiency, presumably, due to loss of contact with the inner network of preorganized water molecules. In contrast, in *meta*-substituted macrocycles and parent **MC3** WOC the twisted orientation of the axial ligands results in available Ru centers for coordination of water molecules which reflects in their significantly higher catalytic performance both in chemical and photocatalytic water oxidation. Therefore, we conclude that rigidification of supramolecular catalysts by a strategic modification of the ligands that kept the Ru active centers permanently accessible and in contact with each other *via* a hydrogen-bonded water network would lead to high performance water oxidation catalysts.

## Conflicts of interest

There are no conflicts to declare.

## Supplementary Material

SC-011-D0SC01097A-s001

SC-011-D0SC01097A-s002

## References

[cit1] The Intergovernmental Panel on Climate Change (IPCC), Climate Change 2013: The Physical Science Basis, Cambridge Univ. Press, 2013

[cit2] The Intergovernmental Panel on Climate Change (IPCC), Climate Change 2014: Mitigation of Climate Change, Cambridge Univ. Press, 2014

[cit3] Frischmann P. D., Mahata K., Würthner F. (2013). Chem. Soc. Rev..

[cit4] Berardi S., Drouet S., Francàs L., Gimbert-Suriñach C., Guttentag M., Richmond C., Stoll T., Llobet A. (2014). Chem. Soc. Rev..

[cit5] Zhang B., Sun L. (2019). Chem. Soc. Rev..

[cit6] Kärkäs M. D., Verho O., Johnston E. V., Åkermark B. (2014). Chem. Rev..

[cit7] Schilling M. and Luber S., Advances in Inorganic Chemistry, Academic Press, 2019, vol. 74, pp. 61–114

[cit8] Hessels J., Detz R. J., Koper M. T. M., Reek J. N. H. (2017). Chem.–Eur. J..

[cit9] Matheu R., Garrido-Barros P., Gil-Sepulcre M., Ertem M. Z., Sala X., Gimbert-Suriñach C., Llobet A. (2019). Nat. Rev. Chem..

[cit10] Blakemore J. D., Crabtree R. H., Brudvig G. W. (2015). Chem. Rev..

[cit11] Kärkäs M. D., Åkermark B. (2016). Dalton Trans..

[cit12] Kunz V., Schmidt D., Röhr M. I. S., Mitrić R., Würthner F. (2017). Adv. Energy Mater..

[cit13] Matheu R., Ertem M. Z., Gimbert-Suriñach C., Sala X., Llobet A. (2019). Chem. Rev..

[cit14] Staehle R., Tong L., Wang L., Duan L., Fischer A., Ahlquist M. S. G., Sun L., Rau S. (2014). Inorg. Chem..

[cit15] Zhang B., Sun L. (2019). J. Am. Chem. Soc..

[cit16] Duan L., Bozoglian F., Mandal S., Stewart B., Privalov T., Llobet A., Sun L. (2012). Nat. Chem..

[cit17] Duan L., Araujo C. M., Ahlquist M. S. G., Sun L. (2012). Proc. Natl. Acad. Sci. U. S. A..

[cit18] Jiang Y., Li F., Huang F., Zhang B., Sun L. (2013). Chin. J. Catal..

[cit19] Duan L., Wang L., Inge A. K., Fischer A., Zou X., Sun L. (2013). Inorg. Chem..

[cit20] Wang L., Duan L., Wang Y., Ahlquist M. S. G., Sun L. (2014). Chem. Commun..

[cit21] Richmond C. J., Matheu R., Poater A., Falivene L., Benet-Buchholz J., Sala X., Cavallo L., Llobet A. (2014). Chem.–Eur. J..

[cit22] Sato Y., Takizawa S.-Y., Murata S. (2015). Eur. J. Inorg. Chem..

[cit23] Xie Y., Shaffer D. W., Concepcion J. J. (2018). Inorg. Chem..

[cit24] Yu F., Poole D., Mathew S., Yan N., Hessels J., Orth N., Ivanović-Burmazović I., Reek J. N. H. (2018). Angew. Chem., Int. Ed..

[cit25] Richmond C. J., Escayola S., Poater A. (2019). Eur. J. Inorg. Chem..

[cit26] Li T.-T., Zhao W.-L., Chen Y., Li F.-M., Wang C.-J., Tian Y.-H., Fu W.-F. (2014). Chem.–Eur. J..

[cit27] Liu Z., Gao Y., Yu Z., Zhang M., Liu J. (2015). Chin. J. Catal..

[cit28] Shaffer D. W., Xie Y., Szalda D. J., Concepcion J. J. (2016). Inorg. Chem..

[cit29] Scherrer D., Schilling M., Luber S., Fox T., Spingler B., Alberto R., Richmond C. J. (2016). Dalton Trans..

[cit30] Schulze M., Kunz V., Frischmann P. D., Würthner F. (2016). Nat. Chem..

[cit31] Kunz V., Lindner J. O., Schulze M., Röhr M. I. S., Schmidt D., Mitrić R., Würthner F. (2017). Energy Environ. Sci..

[cit32] Duan L., Fischer A., Xu Y., Sun L. (2009). J. Am. Chem. Soc..

[cit33] Garrido-Barros P., Gimbert-Suriñach C., Matheu R., Sala X., Llobet A. (2017). Chem. Soc. Rev..

[cit34] Concepcion J. J., Zhong D. K., Szalda D. J., Muckerman J. T., Fujita E. (2015). Chem. Commun..

[cit35] Sheridan M. V., Sherman B. D., Fang Z., Wee K.-R., Coggins M. K., Meyer T. J. (2015). ACS Catal..

[cit36] Kunz V., Schulze M., Schmidt D., Würthner F. (2017). ACS Energy Lett..

[cit37] Juris A., Balzani V., Barigelletti F., Campagna S., Belser P., von Zelewsky A. (1988). Coord. Chem. Rev..

[cit38] Parent A. R., Crabtree R. H., Brudvig G. W. (2013). Chem. Soc. Rev..

[cit39] Berardi S., Francàs L., Neudeck S., Maji S., Benet-Buchholz J., Meyer F., Llobet A. (2015). ChemSusChem.

[cit40] Limburg B., Bouwman E., Bonnet S. (2016). ACS Catal..

[cit41] Francàs L., Matheu R., Pastor E., Reynal A., Berardi S., Sala X., Llobet A., Durrant J. R. (2017). ACS Catal..

[cit42] Natali M., Nastasi F., Puntoriero F., Sartorel A. (2019). Eur. J. Inorg. Chem..

[cit43] Xue L.-X., Meng T.-T., Yang W., Wang K.-Z. (2015). J. Photochem. Photobiol., B.

[cit44] Weinberg D. R., Gagliardi C. J., Hull J. F., Murphy C. F., Kent C. A., Westlake B. C., Paul A., Ess D. H., McCafferty D. G., Meyer T. J. (2012). Chem. Rev..

[cit45] Vigara L., Ertem M. Z., Planas N., Bozoglian F., Leidel N., Dau H., Haumann M., Gagliardi L., Cramer C. J., Llobet A. (2012). Chem. Sci..

[cit46] Lin J., Liang X., Cao X., Wei N., Ding Y. (2018). Chem. Commun..

[cit47] Agmon N. (1995). Chem. Phys. Lett..

[cit48] Kern J., Chatterjee R., Young I. D., Fuller F. D., Lassalle L., Ibrahim M., Gul S., Fransson T., Brewster A. S., Alonso-Mori R., Hussein R., Zhang M., Douthit L., de Lichtenberg C., Cheah M. H., Shevela D., Wersig J., Seuffert I., Sokaras D., Pastor E., Weninger C., Kroll T., Sierra R. G., Aller P., Butryn A., Orville A. M., Liang M., Batyuk A., Koglin J. E., Carbajo S., Boutet S., Moriarty N. W., Holton J. M., Dobbek H., Adams P. D., Bergmann U., Sauter N. K., Zouni A., Messinger J., Yano J., Yachandra V. K. (2018). Nature.

[cit49] Bommer M., Bondar A.-N., Zouni A., Dobbek H., Dau H. (2016). Biochemistry.

[cit50] Singh H., Vasa S. K., Jangra H., Rovó P., Päslack C., Das C. K., Zipse H., Schäfer L. V., Linser R. (2019). J. Am. Chem. Soc..

