# Supplementary material for: Impact of substituents on molecular properties and catalytic activities of trinuclear Ru macrocycles in water oxidation
Source: Chem Sci. 2020 Apr 29;11(29):7654–64. doi: 10.1039/d0sc01097a (PMC8159484; doi:10.1039/d0sc01097a)
Supplement: SC-011-D0SC01097A-s001 [file SC-011-D0SC01097A-s001.pdf]

Electronic Supplementary Material (ESI) for Chemical Science.
This journal is © The Royal Society of Chemistry 2020

Electronic Supplementary Information

Impact of substituents on molecular properties and catalytic activities of supramolecular Ru macrocycles in water oxidation

Ana-Lucia Meza-Chincha, Joachim O. Lindner, Dorothee Schindler, David Schmidt, Ana-Maria Krause, Merle I. S. Röhr, Roland Mitrić and Frank Würthner*

Table of contents

1. Materials and methods S2
2. Synthetic procedures S6
3. X-ray crystal structure analysis S16
4. Redox properties S17
5. UV/Vis absorption spectroscopy and spectroelectrochemistry S23
6. Chemical water oxidation S25
7. Photocatalytic water oxidation S31
8. Theoretical studies S36
9. PXRD S37
10. NMR spectra S38
11. HR mass spectra S54

References S58

1. Materials and methods

General

Reagents and solvents were purchased from commercial sources and used as received. The compounds 2,2'-bipyridine-6,6'-dicarboxylic acid,[1, 2] [$RuCl_2$(dmso)$_4$],[3-5] [Ru(bda)(dmso)$_2$],[6, 7] 6,6'-dibromo-4,4'-dimethoxy-2,2'-bipyridine[8] and 3-bromo-4-methoxy-pyridine[9, 10] were synthesized according to literature procedures. The ligand 1,4-bis(pyridin-3-yl)benzene (bpb)[11] and the Ru macrocycle **MC3**[11] were prepared as described in preceding publications of our group. Anhydrous THF and methanol were prepared using a Pure Solv MD-5 solvent purification system (Innovative Technology). All experiments in aqueous solutions were performed in either phosphate buffer pH 7 (Honeywell) or deionized water obtained from a Purelab Classic water purification system (ELGA). Analytical TLC was carried out on sheets pre-coated with silica gel (Alugram Xtra Sil G/UV$_{254}$, Macherey-Nagel) or aluminum oxide (Polygram Alox N/UV254, Macherey-Nagel). Compounds were purified, where specified, by column chromatography using silica gel (60M, 0.04-0.063 mm, Macherey-Nagel) or neutral aluminum oxide (EcoChrom MP Alumina N Act. V, MP Biomedicals). For size exclusion chromatography BioBeads particles (S-X3, Bio-Rad) suspended in a 9:1 mixture of DCM/MeOH were used. ^{1}H-NMR spectra were recorded at 25 °C, unless otherwise noted, at 400 MHz or 600 MHz using a Bruker Avance III HD 400 or Bruker Avance III HD 600 spectrometer, respectively. Proton decoupled ^{13}C-NMR spectra were recorded at 100 MHz or 150 MHz using the same spectrometers, respectively. Chemical shifts δ are indicated in parts per million (ppm) relative to residual undeuterated solvent signals[12] and coupling constants J in Hz. Signal multiplicity is described using the following abbreviations: s = singlet, d = doublet, t = triplet and m = multiplet. 2D-NMR spectra (COSY, NOESY, HSQC and HMBC) were recorded to allow for a correct interpretation of the 1D-NMR spectra of novel compounds. HR-ESI mass spectra were recorded on an ESI micrOTOF focus mass spectrometer (Bruker Daltonics). HR-MALDI spectra were measured on an Autoflex II spectrometer (Bruker Daltonics). All spectra were recorded in positive ion mode. DCTB was used as matrix for MALDI measurements. Elemental analysis was performed using a Vario MICRO cube (Elementar Analysensysteme). For elemental analysis samples were dried under high vacuum at 60 °C for 12 h. Samples were then prepared in the Glovebox under inert atmosphere. Melting points were determined using a B-545 melting point apparatus (Büchi) or a BX41 optical microscope (Olympus) and are uncorrected.

X-ray crystal structure analysis

Single crystals of ***m*-F-MC3** and ***p*-F-MC3** were obtained by slow evaporation of a DCM/MeOH solution of the macrocycles under an argon atmosphere. X-ray data was recorded at 100 K on a Bruker D8

Quest Kappa diffractometer using Cu K_α radiation (λ = 1.54178 Å) from an Incoatec IµS microsource with Montel multi layered mirror with a Photon II CPAD detector. The structures were solved using direct methods, expanded with Fourier techniques and refined with the software package SHELX.[13] Non-hydrogen atoms were refined anisotropically. Hydrogen atoms were included in the structure factor calculation on geometrically idealized positions.

***m*-F-MC3**: The macrocyclic structure was refined as a two-component twin. Diffuse electron density originating from solvent molecules was located inside the macrocyclic cavity. Water molecules were assigned to the maxima of electron density, although the origin of these Q-peaks could not be elucidated unambiguously. However, the resulting hydrogen bonding network is in good agreement with molecular dynamics simulations. Remaining Q-peaks below 1 have not been assigned to water molecules anymore which could have been acceptor sides for H42A and H42B.
Crystal data for ***m*-F-MC3**: *M*r = 2086.81, trigonal space group R-3, a = 23.4927(5) Å, α = 90°, b = 23.4927(5) Å, β = 90°, c = 32.5166(7) Å, γ = 120°, V = 15541.8(7) Å^3, Z = 6, ρ = 1.338 g cm^{-3}, μ = 4.194 mm^{-1}, $F_{(000)}$ = 6352.0, $GooF(F^2)$ = 1.157, R = 0.0385, wR^2 = 0.1181, 6705 unique reflections [$\vartheta \leq$ 72.421°] with a completeness of 100% and 452 parameters.

***p*-F-MC3**: The macrocyclic structure was refined as a two-component twin. Residual electron density resulting solvent molecules could not be modeled satisfactorily. Therefore, the PLATON squeeze routine was applied to remove the respective electron density.[14] The remaining structure could be refined nicely.
Crystal data for ***p*-F-MC3**: *M*r = 1834.56, triclinic space group P-1, a = 15.9552(3) Å, α = 65.145(1)°, b = 18.9386(4) Å, β = 87.292(1)°, c = 21.0317(4) Å, γ = 76.631(1)°, V = 5601.2(2) Å^3, Z = 2, ρ = 1.088 g cm^{-3}, μ = 3.742 mm^{-1}, $F_{(000)}$ = 1836.0, $GooF(F^2)$ = 1.026, R = 0.0407, wR^2 = 0.0997, 18147 unique reflections [$\vartheta \leq$ 72.520°] with a completeness of 99.5% and 1054 parameters.

Crystallographic data have been deposited with the Cambridge Crystallographic Data Centre as supplementary publication no. CCDC 1985319 (***m*-F-MC3**) and 1985320 (***p*-F-MC3**). These data can be obtained free of charge from the Cambridge Crystallographic Data Centre via www.ccdc.ac.uk/data.request/cif.

Electrochemistry

A Cell Stand C3 (BAS Epsilon) with a standard three-electrode configuration was used to perform cyclic voltammetry (CV) and differential pulse voltammetry (DPV). For measurements in organic media,

samples were dissolved in anhydrous DCM (*c* = 0.25 mM) and tetrabutylammonium hexafluorophosphat was added as electrolyte (*c* = 0.1 M). A Platinum disk and wire as well as a Ag/AgCl electrode were used as working, auxiliary and pseudo-reference electrodes, respectively. Ferrocene (Fc) was added at the end of each experiment as an internal standard (Fc^+/Fc = +0.63 V *vs*. NHE).[15] For measurements in aqueous media, glassy carbon was used as working electrode, a Platinum wire as counter electrode and a Ag/AgCl (3 M KCl) electrode as reference electrode (Ag^+/Ag = +0.21 V *vs*. NHE).[16] Samples were dissolved in aqueous mixtures with acetonitrile or TFE as organic cosolvents (*c* = 0.25 mM). If not otherwise stated, CV and DPV were recorded at a scan rate of 100 mV s^{-1} and 20 mV s^{-1}, respectively.

Spectroelectrochemistry

Spectroelectrochemistry in reflexion mode was perfomed using a Agilent Cary 5000 spectrometer in combination with a home-built sample compartment consisting of a cylindrical PTFE cell with a sapphire window and an adjustable three-in-one electrode (6 mm Platinum disk working electrode, 1 mm Platinum counter and pseudo-reference electrode). All experiments were carried out at a sample concentration of *c* = 0.24 mM in 1:1 acetonitrile/water (pH 7, phosphate buffer) with a layer thickness of about 100 µm. The potential was referenced to the first oxidation event as it was determined by DPV.

UV/Vis absorption and emission spectroscopy

UV/Vis absorption spectra were recorded at 25 °C using a Jasco V-670 spectrometer. Samples prepared with spectroscopic grade solvents were measured in 1 cm quartz cuvettes.

Chemical water oxidation

Chemical water oxidation experiments were performed at 20 °C in reaction vessels connected to SSCDANN030PAAA5 pressure sensors (Honeywell, absolute pressure, 0 to 30 psi). For each measurement, 1 g (1.82 mmol) ceric ammonium nitrate (CAN) was dissolved in 3 mL of aqueous mixtures (pH 1, triflic acid) with acetonitrile or TFE as organic cosolvent. 400 µL of the catalyst stock solution was then injected through a septum using a Hamilton syringe. To determine the gas composition at the end of gas evolution, 500 µL of the gas head space was injected into a gas chromatograph GC-2010 Plus (Shimadzu, thermal conductivity detector at 30 mA, argon as carrier gas) using a gas tight Hamilton syringe. TON was calculated based on the total amount of oxygen evolved during catalysis divided by the amount of catalyst injected. The amount of evolved oxygen was determined by the pressure increase in the reaction vessel using the ideal gas law: $\Delta p\ V = R\ T\ \Delta n$,

where T = 293.15 K, R = 8.314 J K^{-1} mol^{-1}, V = 20.6 mL. In concentration-dependent experiments a TON was calculated for each concentration and the highest TON is reported. For calculation of TOF, the initial rate of catalysis was determined again at each concentration by the linear regression of the oxygen evolution curve during the first two seconds of reaction. TOF was then determined from the slope of the plot of the initial rates *vs.* catalyst amount.

Photocatalytic water oxidation

An Oxygraph Plus Clark-electrode system (Hansatech Instruments) was used for oxygen detection in photocatalytic water oxidation experiments. Samples were irradiated using a 150 W xenon lamp (Newport) equipped with a 400 nm cutoff filter. Irradiation was calibrated to 100 mW cm^{-1}, unless otherwise stated, using a PM 200 optical power meter with a S121C sensor (Thorlabs) in combination with a CCS 200/M wide range spectrometer (Thorlabs). For each measurement, a stock solution of the respective PS and sodium persulfate in the indicated solvent mixture was prepared in the dark. An aliquot of this solution was then mixed with the catalyst at variable concentrations and transferred to the transparent reaction chamber while kept in the dark. Irradiation was started at 50 s to allow thermal equilibration of the sample in the temperature-controlled chamber at 20 °C. TON was calculated based on the maximum amount of oxygen evolved during catalysis divided by the amount of catalyst present. In concentration-dependent experiments a TON was calculated for each concentration and the highest TON is reported. For calculation of TOF, the initial rate of catalysis was determined at each concentration by linear regression of the oxygen evolution curve during the first five to ten seconds of reaction direct after the initial induction period (~1 s). TOF was then determined from the slope of the plot of the initial rates *vs.* catalyst amount.

Theoretical simulations

For the metadynamics simulations the electronic structure of the trinuclear Ru macrocycles has been described by the semiempirical PM6 method[17] using the MOPAC2016 program package[18] version 17.279L. The Newtonian equations of motion were integrated for a total of 1 ns in time steps of 2 fs using the velocity Verlet algorithm.[19] During the dynamics, the temperature was kept constant using a velocity-rescaling thermostat at 300 K allowing for canonical sampling.[20] For the evaluation of the torsional distortion of the axial bridging ligands, the two torsion angles between the aromatic rings in one of the bridging ligands have been used as collective variables. Gaussians of 20° width and 0.1 kcal mol^{-1} height were added to the metadynamics potential every 200 time steps. For the rotation of the Ru-bda moieties according to the collective variable visualized in Fig. S52, Gaussians with a width of 7.5° and a height of 0.015 kcal mol^{-1} were added every 1000 time steps. In order to account for the

slower energy deposition rate, total trajectory lengths of 2 ns have been realized in this case. All metadynamics simulations were performed using the metaFALCON python package.[21] Additionally, structure optimizations have been performed on the semiempirical level as described before, as well as using density functional theory (DFT) in Turbomole V7.0[22] employing the PBE exchange-correlation functional[23] together with the def2-SVP basis set[24] and the corresponding effective core potentials (ECP)[25] on ruthenium atoms. In all calculations, solvation was treated implicitly using the COSMO model for water.[26]

Powder X-ray diffraction (PXRD)

PXRD was performed in reflection mode on a Bruker D8 Discovery diffractometer with position-sensitive 1D-Lynxeye detector using Cu-Kα radiation. Crystalline samples of ***m*-F-MC3** were dried under high vacuum at 60 °C.

2. Synthetic procedures

Synthesis of Ru macrocycles functionalized at bridging ligand

The macrocycles ***m*-X-MC3** and ***p*-X-MC3** (X: MeO, Me or F) were prepared as depicted in Fig. 1 in main article. Compound **3** was synthesized according to literature procedures.[3-7]

1,4-bis(4,4,5,5-tetramethyl-1,3,2-dioxaborolan-2-yl)benzene (1)

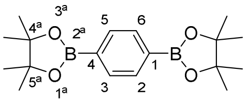

1,4-Dibromobenzene (3.00 g, 12.7 mmol, 1.0 equiv), bis(pinacolato)diboron (9.69 g, 38.2 mmol, 3.0 equiv) and potassium acetate (7.49 g, 76.3 mmol, 6.0 equiv) were dissolved in degassed dioxane (120 mL) under nitrogen. After addition of Pd(dppf)Cl_2 (0.93 g, 1.3 mmol, 0.1 equiv), the mixture was heated at 85 °C for 17.5 h. Afterwards, water (250 mL) was added and the organic phase extracted with ethyl acetate (4 x 100 mL). The combined organic phases were washed with brine and dried over anhydrous Na_2SO_4. The residue was purified by column chromatography (SiO_2, hexane/EtOAc 10:1) and washed with MeOH to yield **1** as a white solid (3.17 g, 9.61 mmol, 76%).

Melting point: 244 °C. ^{1}H-NMR (400 MHz, $CDCl_3$): δ [ppm] = 7.80 (s, 4H, *H*-2,3,5,6), 1.35 (s, 24H, C*H*$_3$).

Analytical data in accordance with the literature.[27]

***m*-MeO-bpb**

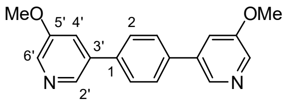

3-Bromo-5-methoxypyridine (239 mg, 1.27 mmol, 2.1 equiv) and **1** (200 mg, 606 µmol, 1.0 equiv) were dissolved in a degassed mixture of toluene (2.4 mL), ethanol (0.6 mL) and 2 M Na_2CO_3 (2.4 mL) under a nitrogen atmosphere. After addition of $Pd(PPh_3)_4$ (70 mg, 60 µmol, 0.1 equiv), the mixture was heated under reflux (105 °C) for 5 days. Afterwards, the solvent was removed under reduced pressure and the residue purified by column chromatography (SiO_2, DCM/EtOAc 5:1 to 1:1) to yield ***m*-MeO-bpb** as a white solid (147 mg, 503 µmol, 83%).

Melting point: 217 °C (decomposition). ^{1}H-NMR (400 MHz, CD_2Cl_2): δ [ppm] = 8.50 (d, $^4J_{H\text{-}H}$ = 1.6 Hz, 2H, *H*-2'), 8.30 (d, $^4J_{H\text{-}H}$ = 2.7 Hz, 2H, *H*-6'), 7.73 (s, 4H, *H*-2), 7.45 (dd, $^4J_{H\text{-}H}$ = 2.7 Hz, $^4J_{H\text{-}H}$ = 1.6 Hz, 2H, *H*-4'), 3.93 (s, 6H, O-C*H*$_3$). ^{13}C-NMR (100 MHz, CD_2Cl_2/CD_3OD): δ [ppm] = 156.3, 140.9, 137.9, 136.9, 136.8, 128.2, 119.0, 56.1. HR-MS (ESI$^+$, MeCN/$CHCl_3$ 1:1): *m/z* calculated for $[M+H]^+$ ($[C_{18}H_{17}N_2O_2]^+$): 293.1285, found: 293.1281 (error: 1.4 ppm). Elemental analysis (%): calculated for $C_{18}H_{16}N_2O_2$: C 73.95, H 5.52, N 9.58, found: C 73.55, H 5.56, N 9.70.

***m*-F-bpb**

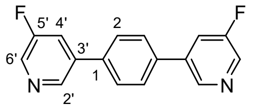

3-Bromo-5-fluoropyridine (660 mg, 3.74 mmol, 2.5 equiv) was dissolved in dry THF (40 mL) under nitrogen. 2 M Na_2CO_3 (7.6 mL) was then added. The mixture was degassed for 10 min before **1** (500 mg, 1.51 mmol, 1.0 equiv) and $Pd(PPh_3)_4$ (175 mg, 151 µmol, 0.1 equiv) were added. The resulting mixture was heated under reflux (70 °C) for 24 h. After cooling to rt, the white crystalline product was collected by vacuum filtration and washed with water to remove Na_2CO_3 traces. The remaining residue was purified by column chromatography (SiO_2, DCM/EtOAc 10:1 to 1:1) to yield ***m*-F-MC3** as a white solid (362 mg, 1.35 mmol, 89%).

Melting point: 193 °C (decomposition). ^{1}H-NMR (400 MHz, CD_2Cl_2): δ [ppm] = 8.73 (t, $^4J_{H\text{-}H}$ = 1.8 Hz, 2H, *H*-2'), 8.48 (d, $^4J_{H\text{-}H}$ = 2.7 Hz, 2H, *H*-6'), 7.75 (s, 4H, *H*-2), 7.68 (ddd, $^3J_{H\text{-}F}$ = 9.7 Hz, $^4J_{H\text{-}H}$ = 2.7 Hz, $^4J_{H\text{-}H}$ = 1.8 Hz, 2H, *H*-4'). ^{13}C-NMR (100 MHz, CD_2Cl_2/CD_3OD): δ [ppm] = 160.2 (d, $^1J_{C\text{-}F}$ = 254.0 Hz), 144.5 (d, $^4J_{C\text{-}F}$ = 3.8 Hz), 137.6 (d, $^3J_{C\text{-}F}$ = 4.0 Hz), 137.3 (d, $^2J_{C\text{-}F}$ = 23.1 Hz), 137.1 (d, $^4J_{C\text{-}F}$ = 1.0 Hz), 128.4, 121.2 (d, $^2J_{C\text{-}F}$ = 18.7 Hz). HR-MS (ESI$^+$, MeCN/$CHCl_3$ 1:1): *m/z* calculated for $[M+H]^+$ ($[C_{16}H_{11}F_2N_2]^+$): 269.0885, found: 269.0885 (error: -0.1 ppm). Elemental analysis (%): calculated for $C_{16}H_{10}F_2N_2$: C 71.64, H 3.76, N 10.44, found: C 71.14, H 4.03, N 9.94.

***m*-Me-bpb**

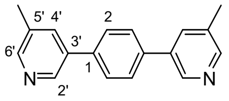

3-Bromo-5-methylpyridine (1.09 g, 6.36 mmol, 2.1 equiv) and **1** (1.00 g, 3.03 mmol, 1.0 equiv) were dissolved in a degassed mixture of toluene (12 mL), ethanol (3 mL) and 2 M Na_2CO_3 (12 mL) under nitrogen. After addition of $Pd(PPh_3)_4$ (350 mg, 303 µmol, 0.1 equiv), the mixture was heated under reflux (105 °C) for 5 days. Afterwards, the solvent was removed *in vacuo* and the residue purified by column chromatography (SiO_2, DCM/EtOAc 5:1 to 1:1) to yield ***m*-Me-bpb** as a white solid (560 mg, 2.15 mmol, 71%).

Melting point: 206 °C. ^{1}H-NMR (400 MHz, CD_2Cl_2): δ [ppm] = 8.69 (d, $^4J_{H-H}$ = 2.1 Hz, 2H, *H*-2'), 8.42 (dd, $^4J_{H-H}$ = 2.1 Hz, $^4J_{H-H}$ = 0.7 Hz, 2H, *H*-6'), 7.76 (ddd, $^4J_{H-H}$ = 2.1 Hz, $^4J_{H-H}$ = 2.1 Hz, $^4J_{H-H}$ = 0.7 Hz, 2H, *H*-4'), 7.72 (s, 4H, *H*-2), 2.41 (d, $^4J_{H-H}$ = 0.7 Hz, 2H, C*H*$_3$). ^{13}C-NMR (100 MHz, CD_2Cl_2): δ [ppm] = 149.2, 145.7, 138.0, 135.6, 135.0, 133.6, 128.0, 18.6. HR-MS (ESI$^+$, MeOH/$CHCl_3$ 1:1): *m/z* calculated for $[M+H]^+$ ($[C_{18}H_{17}N_2]^+$): 261.1386, found: 261.1390 (error: -1.6 ppm). Elemental analysis (%): calculated for $C_{18}H_{16}N_2$: C 83.04, H 6.19, N 10.76, found: C 82.60, H 6.30, N 10.90.

***p*-MeO-bpb**

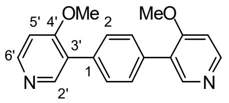

3-Bromo-4-methoxypyridine (1.2 g, 6.36 mmol, 2.1 equiv) and **1** (1.00 g, 3.03 µmol, 1.0 equiv) were dissolved in a degassed mixture of toluene (12 mL), ethanol (3 mL) and 2 M Na_2CO_3 (12 mL) under a nitrogen atmosphere. After addition of $Pd(PPh_3)_4$ (350 mg, 303 µmol, 0.1 equiv), the mixture was heated under reflux (105 °C) for 5 days. Afterwards, the solvent was removed under reduced pressure and the residue purified by column chromatography (SiO_2, EtOAc/MeOH 100:0 to 98:2) to yield ***p*-MeO-bpb** as a white solid (561 mg, 1.92 mmol, 63%).

Melting point: 225 °C (decomposition). ^{1}H-NMR (400 MHz, CD_2Cl_2): δ [ppm] = 8.47 (d, $^3J_{H-H}$ = 5.7 Hz, 2H, *H*-6'), 8.45 (s, 2H, *H*-2'), 7.58 (s, 4H, *H*-2), 6.94 (d, $^3J_{H-H}$ = 5.7 Hz, 2H, *H*-5'), 3.90 (s, 6H, O-C*H*$_3$). ^{13}C-NMR (100 MHz, CD_2Cl_2): δ [ppm] = 162.9, 151.0, 151.0, 134.7, 129.7, 126.3, 106.9, 55.8. HR-MS (ESI$^+$, MeCN/$CHCl_3$ 1:1): *m/z* calculated for $[M+H]^+$ ($[C_{18}H_{17}N_2O_2]^+$): 293.1285, found: 293.1287 (error: -1.0 ppm). Elemental analysis (%): calculated for $C_{18}H_{16}N_2O_2$: C 73.95, H 5.52, N 9.58, found: C 73.97, H 5.63, N 9.56.

p-F-bpb

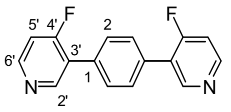

3-Bromo-4-fluoropyridine (1.00 g, 5.68 mmol, 2.5 equiv) was dissolved in dry THF (60 mL) under nitrogen. 2 M Na_2CO_3 (11.4 mL) was then added. The mixture was degassed for 15 min before **1** (750 mg, 2.27 mmol, 1.0 equiv) and $Pd(PPh_3)_4$ (262 mg, 227 µmol, 0.1 equiv) were added. The resulting mixture was heated under reflux (70 °C) for 24 h. After cooling to rt, the residue was purified by column chromatography (SiO_2, DCM/EtOAc 5:1 to 1:1) to yield ***p*-F-MC3** as a white solid (372 mg, 1.39 mmol, 61%).

Melting point: 177 °C (decomposition). ^{1}H-NMR (400 MHz, CD_2Cl_2): δ [ppm] = 8.74 (d, $^4J_{H\text{-}F}$ = 10.4 Hz, 2H, *H*-2'), 8.57 (dd, $^4J_{H\text{-}F}$ = 7.4 Hz, $^3J_{H\text{-}H}$ = 5.7 Hz, 2H, *H*-6'), 7.70 (s, 4H, *H*-2), 7.18 (dd, $^4J_{H\text{-}F}$ = 10.4 Hz, $^3J_{H\text{-}H}$ = 5.7 Hz, 2H, *H*-5'). ^{13}C-NMR (100 MHz, CD_2Cl_2): δ [ppm] = 165.9 (d, $^1J_{C\text{-}F}$ = 262.0 Hz), 152.4 (d, $^3J_{C\text{-}F}$ = 2.8 Hz), 151.7 (d, $^3J_{C\text{-}F}$ = 7.3 Hz), 132.8, 129.8 (d, $^4J_{C\text{-}F}$ = 2.6 Hz), 125.1 (d, $^2J_{C\text{-}F}$ = 9.6 Hz), 112.1 (d, $^2J_{C\text{-}F}$ = 17.7 Hz). HR-MS (ESI$^+$, MeCN/$CHCl_3$ 1:1): *m/z* calculated for $[M+H]^+$ ($[C_{16}H_{11}F_2N_2]^+$): 269.0885, found: 269.0884 (error: 0.1 ppm). Elemental analysis (%): calculated for $C_{16}H_{10}F_2N_2$: C 71.64, H 3.76, N 10.44, found: C 71.57, H 3.71, N 10.71.

p-Me-bpb

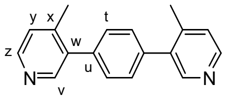

3-Bromo-4-methylpyridine (1.09 g, 6.36 mmol, 2.1 equiv) and **1** (1.00 g, 3.03 mmol, 1.0 equiv) were dissolved in a degassed mixture of toluene (12 mL), ethanol (3 mL) and 2 M Na_2CO_3 (12 mL) under a nitrogen atmosphere. After addition of $Pd(PPh_3)_4$ (350 mg, 303 µmol, 0.1 equiv), the mixture was heated under reflux (105 °C) for 5 days. Afterwards, the solvent was removed *in vacuo* and the residue purified by column chromatography (SiO_2, DCM/EtOAc 5:1 to 1:1) to yield ***p*-Me-bpb** as a white solid (500 mg, 1.92 mmol, 63%).

Melting point: 202 °C. ^{1}H-NMR (400 MHz, CD_2Cl_2): δ [ppm] = 8.46 (d, $^5J_{H\text{-}H}$ = 0.3 Hz, 2H, *H*-2'), 8.44 (d, $^3J_{H\text{-}H}$ = 5.0 Hz, $^5J_{H\text{-}H}$ = 0.3 Hz, 2H, *H*-6'), 7.44 (s, 4H, *H*-2), 7.23 (dq, $^3J_{H\text{-}H}$ = 5.0 Hz, $^4J_{H\text{-}H}$ = 0.7 Hz, 2H, *H*-5'), 2.35 (t, $^5J_{H\text{-}H}$ = 0.3 Hz, 2H, C*H*$_3$). ^{13}C-NMR (100 MHz, CD_2Cl_2): δ [ppm] = 150.3, 148.8, 144.9, 137.7, 137.6, 129.7, 125.6, 20.0. HR-MS (ESI$^+$, MeOH/$CHCl_3$ 1:1): *m/z* calculated for $[M+H]^+$ ($[C_{18}H_{17}N_2]^+$): 261.1386, found: 261.1389 (error: -1.2 ppm). Elemental analysis (%): calculated for $C_{18}H_{16}N_2$: C 83.04, H 6.19, N 10.76, found: C 82.77, H 6.18, N 10.95.

***m*-MeO-MC3**

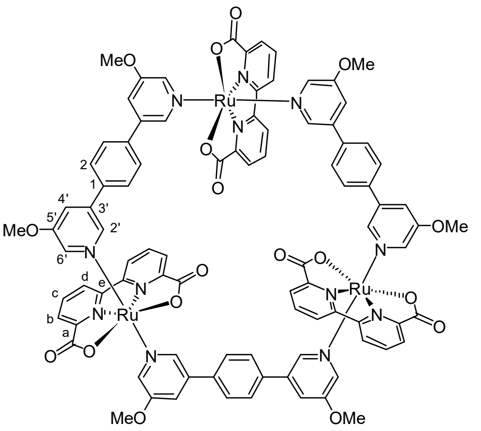

Ru(bda)(dmso)$_2$ **3** (100 mg, 200 µmol, 1.1 equiv) and ***m*-MeO-bpb** (53 mg, 182 µmol, 1.0 eq) were dissolved in a degassed mixture of chloroform (55 mL) and methanol (11 mL) and stirred for 16.5 h at 60 °C. After cooling to rt, the solvent was removed under reduced pressure and the residue purified by column chromatography (Al_2O_3 15% w/w H_2O, DCM/MeOH 6:1 and subsequently SiO_2, DCM/MeOH 10:1 to 2:1) and precipitation with hexane to yield ***m*-MeO-MC3** as a dark brown solid (41 mg, 22 µmol, 36%).

Melting point: >300 °C. ^{1}H-NMR (400 MHz, CD_2Cl_2/CD_3OD): δ [ppm] = 8.52 (dd, $^3J_{H\text{-}H}$ = 7.9 Hz, $^4J_{H\text{-}H}$ = 1.1 Hz, 6H, *H*-d), 8.10 (dd, $^3J_{H\text{-}H}$ = 7.9 Hz, $^4J_{H\text{-}H}$ = 1.1 Hz, 6H, *H*-b), 8.05 (d, $^4J_{H\text{-}H}$ = 1.6 Hz, 6H, *H*-2'), 7.92 (t, $^3J_{H\text{-}H}$ = 7.9 Hz, 6H, *H*-c), 7.50 (s, 12H, *H*-2), 7.27 (dd, $^4J_{H\text{-}H}$ = 2.5 Hz, $^4J_{H\text{-}H}$ = 1.6 Hz, 6H, *H*-6'), 7.08 (d, $^4J_{H\text{-}H}$ = 2.5 Hz, 6H, *H*-4'), 3.72 (s, 18H, O-C*H*$_3$). ^{13}C-NMR (100 MHz, CD_2Cl_2/CD_3OD): δ [ppm] = 173.8, 160.1, 157.7, 156.7, 144.0, 138.8, 137.9, 136.9, 132.3, 128.4, 126.5, 125.0, 119.6, 56.2. HR-MS (ESI$^+$, MeOH/$CHCl_3$ 1:1): *m/z* calculated for $[M]^{2+}$ ($[C_{90}H_{66}N_{12}O_{18}Ru_3]^{2+}$): 954.0869, found: 954.0920 (error: -2.7 ppm). Elemental analysis (%): calculated for $C_{90}H_{66}N_{12}O_{18}Ru_3 \cdot 3\ H_2O$: C 55.13, H 3.70, N 8.57, found: C 55.44, H 3.82, N 8.36.

***m*-F-MC3**

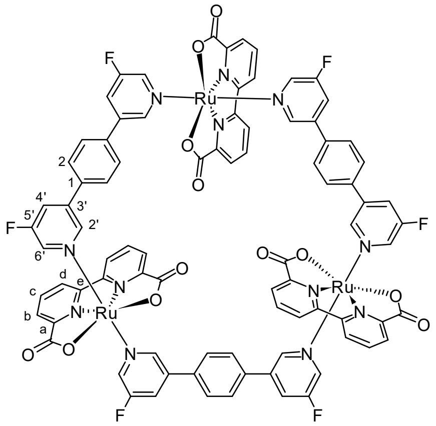

Ru(bda)(dmso)$_2$ (250 mg, 500 µmol, 1.1 equiv) and ***m*-F-bpb** (122 mg, 455 µmol, 1.0 equiv) were dissolved in a degassed mixture of chloroform (138 mL) and methanol (28 mL) and stirred for 16.5 h at 60 °C. After cooling to rt, the solvent was removed *in vacuo* and the residue purified by column chromatography (SiO_2, DCM/MeOH 10:1 to 3:1) and precipitation with hexane to yield ***m*-F-MC3** as a dark brown solid (104 mg, 57 µmol, 38%).

Melting point: >300 °C. ^{1}H-NMR (600 MHz, CD_2Cl_2/CD_3OD): δ [ppm] = 8.56 (dd, $^3J_{H\text{-}H}$ = 7.9 Hz, $^4J_{H\text{-}H}$ = 1.1 Hz, 6H, *H*-d), 8.41 (d, $^4J_{H\text{-}H}$ = 1.7 Hz, 6H, *H*-2'), 8.13 (dd, $^3J_{H\text{-}H}$ = 7.9 Hz, $^4J_{H\text{-}H}$ = 1.1 Hz, 6H, *H*-b), 7.98 (t, $^3J_{H\text{-}H}$ = 7.9 Hz, 6H, *H*-c), 7.59 (ddd, $^3J_{H\text{-}F}$ = 8.5 Hz, $^4J_{H\text{-}H}$ = 2.4 Hz, $^4J_{H\text{-}H}$ = 1.7 Hz, 6H, *H*-4'), 7.57 (s, 12H, *H*-2), 7.28 (t, $^4J_{H\text{-}H}$ = 2.4 Hz, 6H, *H*-6'). ^{13}C-NMR (151 MHz, CD_2Cl_2/CD_3OD): δ [ppm] = 173.7, 159.8 (d,

$^{1}J_{C\text{-}F}$ = 254.8 Hz), 159.8, 157.5, 148.6 (d, $^{4}J_{C\text{-}F}$ = 3.2 Hz), 139.4 (d, $^{2}J_{C\text{-}F}$ = 30.7 Hz), 138.8 (d, $^{3}J_{C\text{-}F}$ = 6.2 Hz), 136.1 (d, $^{4}J_{C\text{-}F}$ = 1.7 Hz), 133.2, 128.7, 126.8, 125.4, 122.5 (d, $^{2}J_{C\text{-}F}$ = 19.1 Hz). HR-MS (ESI^{+}, MeOH/$CHCl_3$ 1:1): *m/z* calculated for $[M+2Na]^{2+}$ ($[C_{84}H_{48}F_6N_{12}Na_2O_{12}Ru_3]^{2+}$): 941.0167, found: 941.0176 (error: -1.6 ppm). Elemental analysis (%): calculated for $C_{84}H_{48}F_6N_{12}O_{12}Ru_3 \cdot H_2O$: C 54.46, H 2.72, N 9.07, found: C 54.53, H 3.27, N 9.42.

***m*-Me-MC3**

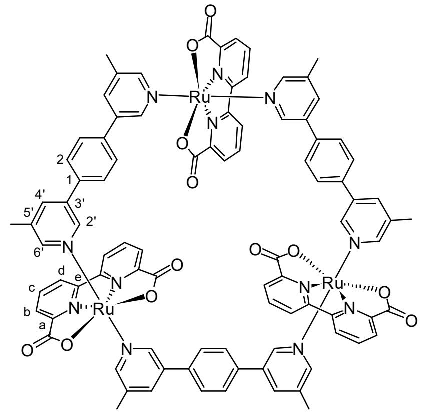

Ru(bda)(dmso)$_2$ (100 mg, 200 µmol, 1.1 equiv) and ***m*-Me-bpb** (47 mg, 182 µmol, 1.0 equiv) were dissolved in a degassed mixture of chloroform (55 mL) and methanol (11 mL) and stirred for 16.5 h at 60 °C. After cooling to rt, the solvent was removed under reduced pressure and the residue purified by column chromatography (Al_2O_3 15% w/w H_2O, DCM/MeOH 6:1, two consecutive times and subsequently SiO_2, DCM/MeOH 10:1 to 4:1) and precipitation with hexane to yield ***m*-Me-MC3** as a dark brown solid (31 mg, 17 µmol, 28%).

Melting point: >300 °C. ^{1}H-NMR (400 MHz, CD_2Cl_2/CD_3OD): δ [ppm] = 8.52 (dd, $^{3}J_{H\text{-}H}$ = 7.9 Hz, $^{4}J_{H\text{-}H}$ = 1.0 Hz, 6H, *H*-d), 8.27 (d, $^{4}J_{H\text{-}H}$ = 1.7 Hz, 6H, *H*-2'), 8.10 (dd, $^{3}J_{H\text{-}H}$ = 7.9 Hz, $^{4}J_{H\text{-}H}$ = 1.0 Hz, 6H, *H*-b), 7.91 (t, $^{3}J_{H\text{-}H}$ = 7.9 Hz, 6H, *H*-c), 7.58 (d, $^{4}J_{H\text{-}H}$ = 0.7 Hz, 6H, *H*-4'), 7.50 (s, 12H, *H*-2), 7.16 (s, 6H, *H*-6'), 2.13 (s, 18H, O-C*H*$_3$). ^{13}C-NMR (100 MHz, CD_2Cl_2/CD_3OD): δ [ppm] = 173.9, 160.2, 157.6, 150.3, 148.9, 137.0, 136.8, 135.9, 135.6, 132.0, 128.3, 126.5, 124.9, 18.5. HR-MS (ESI^{+}, MeOH/$CHCl_3$ 1:1): *m/z* calculated for $[M]^{+}$ ($[C_{90}H_{66}N_{12}O_{12}Ru_3]^{+}$): 1812.2048, found: 1812.2094 (error: -0.3 ppm). Elemental analysis (%): calculated for $C_{90}H_{66}N_{12}O_{18}Ru_3 \cdot 3\,H_2O$: C 57.97, H 3.89, N 9.01, found: C 58.45, H 4.54, N 9.12.

***p*-MeO-MC3:**

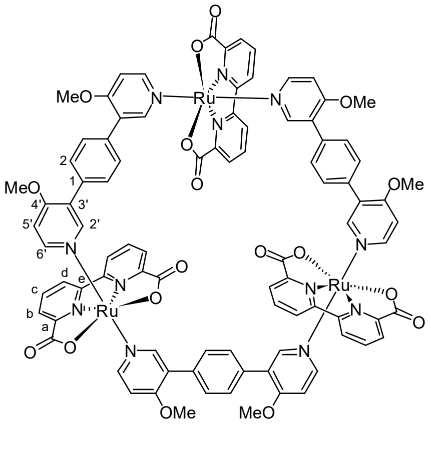

Ru(bda)(dmso)$_2$ (100 mg, 200 µmol, 1.1 equiv) and ***p*-MeO-bpb** (53 mg, 182 µmol, 1.0 equiv) were dissolved in a degassed mixture of chloroform (55 mL) and methanol (11 mL) and stirred for 16.5 h at 60 °C. After cooling to rt, the solvent was removed under reduced pressure and the residue purified by column chromatography (Al_2O_3 15% w/w H_2O, DCM/MeOH 6:1 and subsequently SiO_2, DCM/MeOH 10:1 to 2:1) and precipitation with hexane to yield ***p*-MeO-MC3** as a dark brown solid (37 mg, 19 µmol, 32%).

Melting point: >300 °C. ^{1}H-NMR (400 MHz, CD_2Cl_2/CD_3OD): δ [ppm] = 8.42 (dd, $^3J_{H-H}$ = 7.9 Hz, $^4J_{H-H}$ = 1.1 Hz, 6H, *H*-d), 8.11 (dd, $^3J_{H-H}$ = 7.9 Hz, $^4J_{H-H}$ = 1.1 Hz, 6H, *H*-b), 7.86 (t, $^3J_{H-H}$ = 7.9 Hz, 6H, *H*-c), 7.72 (d, $^4J_{H-H}$ = 1.0 Hz, 6H, *H*-2'), 7.41 (dd, $^4J_{H-H}$ = 6.7 Hz, $^4J_{H-H}$ = 1.0 Hz, 6H, *H*-6'), 7.27 (s, 12H, *H*-2), 6.72 (d, $^4J_{H-H}$ = 6.7 Hz, 6H, *H*-5'), 3.76 (s, 18H, O-C*H*$_3$). ^{13}C-NMR (100 MHz, CD_2Cl_2/CD_3OD): δ [ppm] = 174.0, 163.6, 160.5, 157.7, 152.2, 152.1, 133.5, 131.5, 129.6, 127.8, 126.5, 124.6, 108.7, 56.4. HR-MS (ESI$^+$, MeOH/$CHCl_3$ 1:1): *m/z* calculated for $[M]^{2+}$ ($[C_{90}H_{66}N_{12}O_{18}Ru_3]^{2+}$): 954.0869, found: 954.0906 (error: -1.2 ppm). Elemental analysis (%): calculated for $C_{90}H_{66}N_{12}O_{18}Ru_3 \cdot 3\ H_2O$: C 55.13, H 3.70, N 8.57, found: C 55.29, H 3.92, N 8.14.

***p*-F-MC3:**

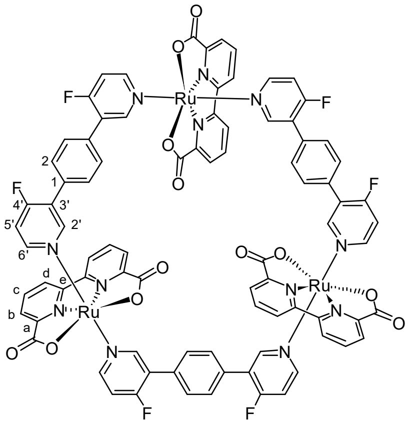

Ru(bda)(dmso)$_2$ (250 mg, 500 µmol, 1.1 equiv) and ***p*-F-bpb** (122 mg, 455 µmol, 1.0 equiv) were dissolved in a degassed mixture of chloroform (138 mL) and methanol (28 mL) and stirred for 16.5 h at 60 °C. After cooling to rt, the solvent was removed *in vacuo* and the residue purified by column chromatography (SiO_2, DCM/MeOH 10:1 to 3:1) and precipitation with hexane to yield ***p*-F-MC3** as a dark brown solid (154 mg, 84 µmol, 55%).

Melting point: >300 °C. ^{1}H-NMR (600 MHz, CD_2Cl_2/CD_3OD): δ [ppm] = 8.50 (dd, $^3J_{H-H}$ = 7.9 Hz, $^4J_{H-H}$ = 0.9 Hz, 6H, *H*-d), 8.28 (d, $^4J_{H-F}$ = 8.1 Hz, 6H, *H*-2'), 8.12 (dd, $^3J_{H-H}$ = 7.9 Hz, $^4J_{H-H}$ = 0.9 Hz, 6H, *H*-b), 7.93 (t, $^3J_{H-H}$ = 7.9 Hz, 6H, *H*-c), 7.48 (s, 12H, *H*-2), 7.41 (t, $^3J_{H-H}$ = 6.3 Hz, 6H, *H*-6'), 6.98 (dd, $^3J_{H-F}$ = 8.7 Hz, $^4J_{H-H}$ = 6.3 Hz, 6H, *H*-5'). ^{13}C-NMR (151 MHz, CD_2Cl_2/CD_3OD): δ [ppm] = 173.8, 166.0 (d, $^1J_{C-F}$ = 270.0 Hz), 160.1, 157.6, 155.3 (d, $^3J_{C-F}$ = 4.5 Hz), 153.1 (d, $^3J_{C-F}$ = 9.0 Hz), 132.6, 131.7, 129.9 (d, $^4J_{C-F}$ = 1.5 Hz), 127.0 (d, $^2J_{C-F}$ = 12.0 Hz), 126.7, 125.1, 114.2 (d, $^2J_{C-F}$ = 20.0 Hz). HR-MS (ESI$^+$, MeOH/$CHCl_3$ 1:1): *m/z* calculated for $[M]^{2+}$ ($[C_{84}H_{48}F_6N_{12}O_{12}Ru_3]^{2+}$): 918.0269, found: 918.0309 (error: -1.7 ppm). Elemental analysis (%): calculated for $C_{84}H_{48}F_6N_{12}O_{12}Ru_3$: C 54.99, H 2.64, N 9.16, found: C 55.36, H 2.91, N 9.16.

***p*-Me-MC3:**

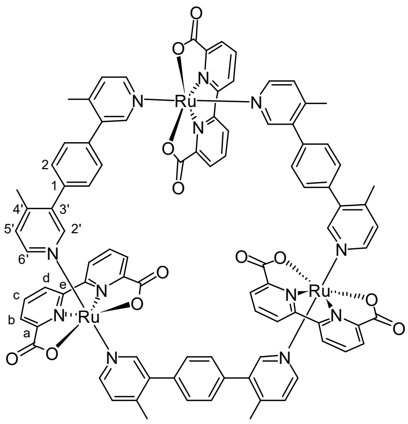

Ru(bda)(dmso)$_2$ (100 mg, 200 µmol, 1.1 equiv) and ***p*-Me-bpb** (47 mg, 182 µmol, 1.0 equiv) were dissolved in a degassed mixture of chloroform (55 mL) and methanol (11 mL) and stirred for 16.5 h at 60 °C. After cooling to rt, the solvent was removed under reduced pressure and the residue purified by column chromatography (Al_2O_3 15% w/w H_2O, DCM/MeOH 6:1, two consecutive times and subsequently SiO_2, DCM/MeOH 10:1 to 4:1) and precipitation with hexane to yield ***p*-Me-MC3** as a dark brown solid (23 mg, 13 µmol, 21%).

Melting point: >300 °C. ^{1}H-NMR (400 MHz, CD_2Cl_2/CD_3OD): δ [ppm] = 8.45 (dd, $^3J_{H\text{-}H}$ = 7.9 Hz, $^4J_{H\text{-}H}$ = 1.0 Hz, 6H, *H*-d), 8.08 (dd, $^3J_{H\text{-}H}$ = 7.9 Hz, $^4J_{H\text{-}H}$ = 1.0 Hz, 6H, *H*-b), 7.87 (t, $^3J_{H\text{-}H}$ = 7.9 Hz, 6H, *H*-c), 7.80 (s, 6H, *H*-2'), 7.38 (d, $^4J_{H\text{-}H}$ = 5.9 Hz, 6H, *H*-6'), 7.16 (s, 12H, *H*-2), 6.98 (d, $^4J_{H\text{-}H}$ = 5.9 Hz, 6H, *H*-5'), 2.18 (s, 18H, O-C*H*$_3$). ^{13}C-NMR (100 MHz, CD_2Cl_2/CD_3OD): δ [ppm] = 174.1, 160.4, 157.5, 152.1, 150.0, 146.9, 138.8, 136.4, 132.0, 129.8, 127.1, 126.5, 125.0, 19.8. HR-MS (ESI$^+$, MeOH/DCM 1:1): *m/z* calculated for $[M]^+$ ($[C_{90}H_{66}N_{12}O_{12}Ru_3]^+$): 1812.2048, found: 1812.2069 (error: -1.6 ppm). Elemental analysis (%): calculated for $C_{90}H_{66}N_{12}O_{18}Ru_3 \cdot H_2O$: C 59.11, H 3.75, N 9.19, found: C 59.05, H 3.38, N 9.00.

Synthesis of Ru macrocycle functionalized at equatorial ligand

The macrocycle **MeO-bda-MC3** containing methoxy groups in the equatorial bda ligand was prepared as depicted in Fig. S1. 6,6'-Dibromo-4,4'-dimethoxy-2,2'-bipyridine **4** was synthesized according to literature procedures.[8]

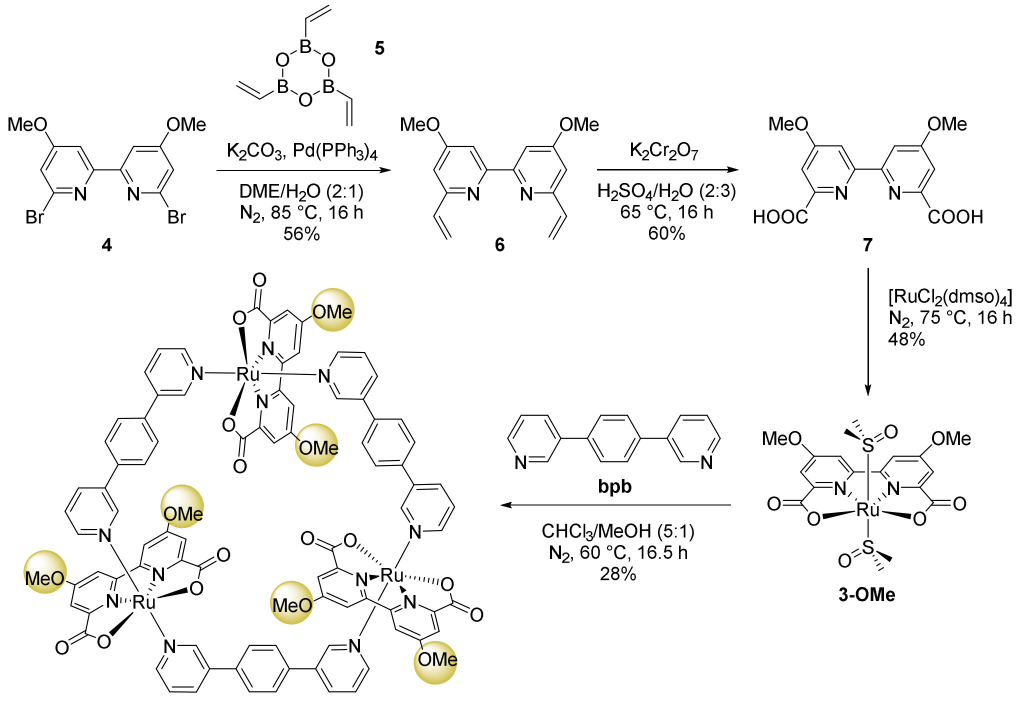

Fig. S1 Synthesis of **MeO-bda-MC3** macrocycle functionalized at the equatorial bda ligand.

4,4'-Dimethoxy-6,6'-divinyl-2,2'-bipyridine (6)

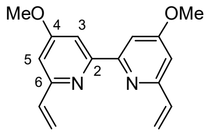

6,6'-Dibromo-4,4'-dimethoxy-2,2'-bipyridine **4** (780 mg, 2.09 mmol, 1.0 equiv) and 2,4,6-trivinylcyclotriboroxane **5** (602 mg, 2.50 mmol, 1.2 equiv) were suspended in a degassed mixture of DME (10 mL) and 2 M K_2CO_3 (5 mL) under nitrogen. After addition of $Pd(PPh_3)_4$ (483 mg, 418 µmol, 0.2 equiv), the mixture was heated under reflux (105 °C) for 16 h. Afterwards, the solvent was removed *in vacuo* and the residue redissolved in EtOAc, filtered and purified by column chromatography (SiO_2, cyclohexane/EtOAc 49:1) to yield **6** as a white solid (382 mg, 1.42 mmol, 68%).

Melting point: 84 °C. ^{1}H-NMR (400 MHz, $CDCl_3$): δ [ppm] = 7.99 (d, $^4J_{H\text{-}H}$ = 2.4 Hz, 2H, *H*-3), 6.87 (d, $^4J_{H\text{-}H}$ = 2.4 Hz, 2H, *H*-5), 6.89 (dd, $^3J_{H\text{-}H}$ = 17.4 Hz, $^3J_{H\text{-}H}$ = 10.7 Hz, 2H, Ar-*H*=CH_2), 6.33 (dd, $^3J_{H\text{-}H}$ = 17.4 Hz, $^4J_{H\text{-}H}$ = 1.5 Hz, 2H, CH=C*H*$_2$), 5.50 (dd, $^3J_{H\text{-}H}$ = 10.7 Hz, $^4J_{H\text{-}H}$ = 1.5 Hz, 2H, CH=C*H*$_2$), 3.96 (s, 6H, OC*H*$_3$). ^{13}C-NMR (100 MHz, $CDCl_3$): δ [ppm] = 167.2, 157.4, 156.3, 137.0, 118.2, 108.3, 105.4, 55.3. HR-MS (ESI$^+$, MeOH/$CHCl_3$ 1:1): *m*/*z* calculated for $[M+H]^+$ ($[C_{16}H_{17}N_2O_2]^+$): 269.1290, found: 269.1286 (error: 1.5 ppm). Elemental analysis (%): calculated for $C_{16}H_{16}N_2O_2$: C 71.62, H 6.01, N 10.44, found: C 71.38, H 6.23, N 10.31.

4,4'-dimethoxy-[2,2'-bipyridine]-6,6'-dicarboxylic acid (7)

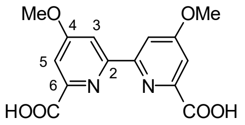

6 (380 mg, 1.42 mmol, 1.0 equiv) was dissolved in concentrated sulfuric acid (10 mL) and added dropwise to a solution of $K_2Cr_2O_7$ (1.25 g, 4.25 mmol, 3.0 equiv) in water (15 mL). The mixture was stirred at 65 °C for 16 h and then poured into ice. Afterwards, the solid was collected by centrifugation and washed with water to yield **7** as a white solid (258 mg, 0.85 mmol, 60%).

Melting point: 245 °C. ^{1}H-NMR (400 MHz, DMSO-d$_6$): δ [ppm] = 8.32 (d, $^4J_{H\text{-}H}$ = 2.5 Hz, 2H, *H*-3), 7.65 (d, $^4J_{H\text{-}H}$ = 2.5 Hz, 2H, *H*-5), 4.01 (s, 6H, OC*H*$_3$). ^{13}C-NMR (100 MHz, DMSO-d$_6$,): δ [ppm] = 167.9, 166.1, 156.3, 150.1, 111.7, 110.5, 56.6. HR-MS (ESI$^+$, MeOH/$CHCl_3$ 1:1): *m/z* calculated for $[M+Na]^+$ ($[C_{14}H_{12}N_2NaO_6]^+$): 327.0588, found: 327.0592 (error: -1.5 ppm).

Ru(MeO-bda)(dmso)$_2$ (3-OMe)

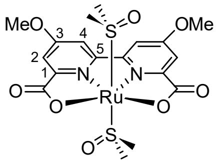

7 (109 mg, 358 µmol, 1.0 equiv) and $RuCl_2(dmso)_4$ (190 mg, 394 µmol, 1.1 equiv) were dissolved in a degassed mixture of anhydrous methanol (20 mL) and triethylamine (1 mL) under nitrogen. The mixture was heated under reflux (75 °C) for 16 h. Afterwards, the solid was collected by centrifugation and washed with methanol to yield **3-OMe** as a brown solid (112 mg, 200 µmol, 56%).

Melting point: 185-190 °C (decomposition). ^{1}H-NMR (400 MHz, DMSO-d$_6$, 399 K): δ [ppm] = 8.24 (s, 2H, *H*-4), 7.56 (d, $^4J_{H\text{-}H}$ = 2.3 Hz, 2H, *H*-2), 4.09 (s, 6H, OC*H*$_3$), 2.54 (s, 12H, O=S(C*H*$_3$)$_2$). HR-MS (ESI$^+$, MeCN/H_2O 1:1): *m/z* calculated for $[M\text{-}dmso+MeCN+H]^+$ ($[C_{18}H_{20}N_3O_7RuS]^+$): 524.0060, found: 524.0042 (error: 3.4 ppm).

MeO-bda-MC3

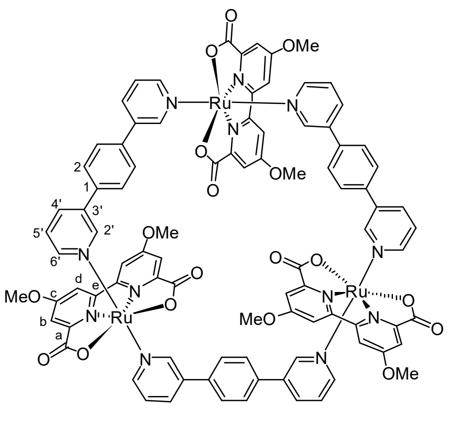

3-OMe (35 mg, 63 µmol, 1.0 equiv) and **bpb** (14.5 mg, 63 µmol, 1.0 eq) were dissolved in a degassed mixture of chloroform (37.5 mL) and methanol (7.5 mL) and stirred for 16.5 h at 60 °C. After cooling to rt, the solvent was removed under reduced pressure and the residue purified by column chromatography (Al_2O_3 15% w/w H_2O, DCM/MeOH 9:1) to yield **MeO-bda-MC3** as a dark brown solid (34 mg, 18 µmol, 28%).

Melting point: >300 °C. ^{1}H-NMR (400 MHz, CD_2Cl_2/CD_3OD + ascorbic acid): δ [ppm] = 8.58 (t, $^{4}J_{H\text{-}H}$ = 2.3 Hz, 6H, *H*-d), 8.21 (d, $^{4}J_{H\text{-}H}$ = 2.3 Hz, 6H, *H*-b), 7.81 (ddd, $^{3}J_{H\text{-}H}$ = 7.8 Hz, $^{4}J_{H\text{-}H}$ = 2.3 Hz, $^{5}J_{H\text{-}H}$ = 1.2 Hz, 6H, *H*-4'), 7.71 (d, $^{4}J_{H\text{-}H}$ = 2.3 Hz, 6H, *H*-2'), 7.61 (d, $^{3}J_{H\text{-}H}$ = 5.5 Hz, 6H, *H*-6'), 7.58 (d, $^{5}J_{H\text{-}H}$ = 1.2 Hz, 12H, *H*-2), 7.16 (dd, $^{3}J_{H\text{-}H}$ = 7.8 Hz, $^{3}J_{H\text{-}H}$ = 5.5 Hz, 6H, *H*-5'), 4.08 (s, 18H, OCH_3). ^{13}C-NMR (100 MHz, CD_2Cl_2/CD_3OD + ascorbic acid): δ [ppm] = 173.9, 165.5, 160.7, 160.5, 158.2, 153.0, 150.8, 137.4, 137.0, 134.8, 128.5, 125.4, 113.3, 57.2. HR-MS (MALDI$^+$, MeOH/$CHCl_3$ 1:1): *m/z* calculated for $[M]^+$ ($[C_{90}H_{66}N_{12}O_{18}Ru_3]^+$): 1908.1743, found: 1908.1790 (error: -2.5 ppm).

3. X-ray crystal structure analysis

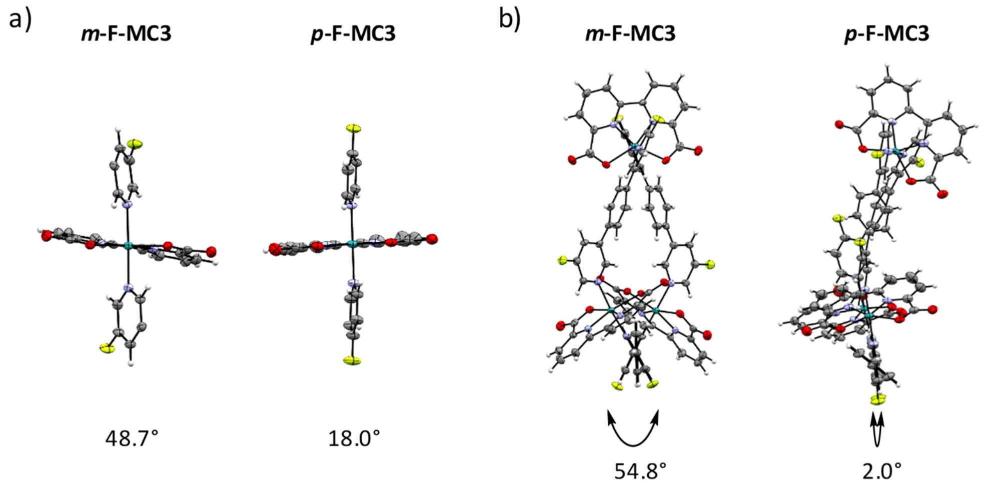

Fig. S2 Comparison of the X-ray crystal structures of ***m*-F-MC3** and ***p*-F-MC3** regarding the torsion angle between the terminal pyridyl rings within a single bridging ligand (a) and the torsion between two axial ligands coordinated to one Ru center (b). Only one Ru center with its coordinated pyridyl rings is shown for each macrocycle in b) for clarity (ORTEP diagram with thermal ellipsoids set at 50% probability; grey: carbon, white: hydrogen, red: oxygen, lavender: nitrogen, turquoise: ruthenium, green-yellow: fluorine).

Table S1 Selected bond lengths and angles of ***m*-F-MC3** and ***p*-F-MC3** in the solid state.

	***m*-F-MC3**	***p*-F-MC3**		
		Ru^1	Ru^2	Ru^3
O-Ru-O [°]	122.8(2)	123.1(1)	121.9(1)	123.0(1)
N_{ax}-Ru-N_{ax} [°]	172.5(2)	176.3(1)	175.1(1)	171.1(1)
d (Ru-N_{ax}) [Å]	2.081(5)	2.089(3)	2.080(2)	2.075(3)
	2.084(4)	2.118(3)	2.099(2)	2.067(3)
Torsion py_{ax}-Ru-py_{ax} [°]	48.7	47.5	29.1	18.0
Torsion py_{ax}-ph-py_{ax} [°]	54.8	71.8	5.3	2.0

4. Redox properties

Table S2 Redox properties of the macrocyclic **MC3** derivatives under neutral conditions.[a]

Macrocycle	*E vs.* NHE [V]		
	Ru^{III}_3/Ru^{II}_3	Ru^{IV}_3/Ru^{III}_3	Ru^{V}_3/Ru^{IV}_3
**MC3**	+0.66	+0.82	+1.00
***m*-MeO-MC3**	+0.66	+0.83	+1.02
***m*-F-MC3**	+0.68	+0.83	+1.06
***m*-Me-MC3**	+0.66	+0.84	+1.02
***p*-F-MC3**	+0.65	+0.84	+1.04
***p*-MeO-MC3**	+0.61	+0.81	+0.98
***p*-Me-MC3**	+0.63	+0.81	+1.00
**MeO-bda-MC3**	+0.57	+0.80	+0.97

[a] CV and DPV measurements were performed in TFE/H_2O 1:1 (phosphate buffer, pH 7), *c* = 0.2 mM.

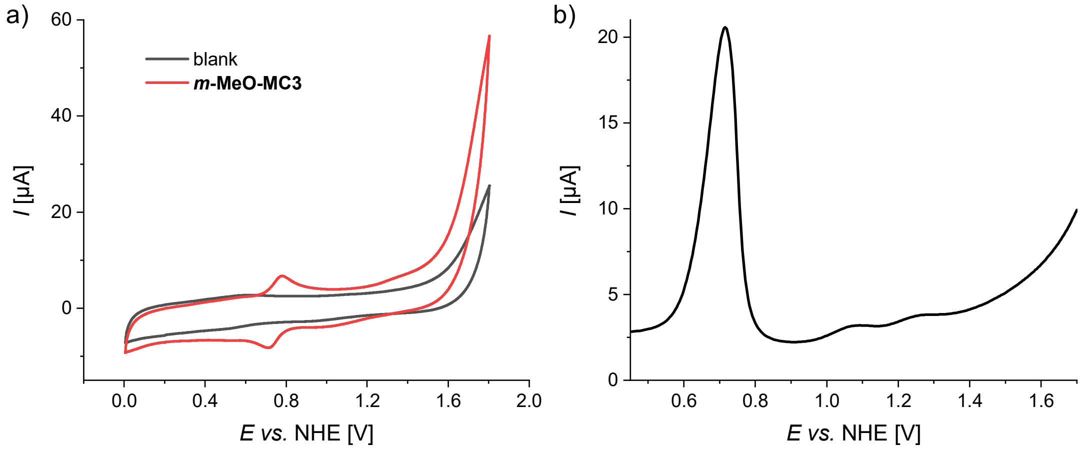

Fig. S3 CV (a) and DPV (b) of ***m*-MeO-MC3** in TFE/H_2O 1:1 (pH 1, triflic acid), *c* = 0.25 mM.

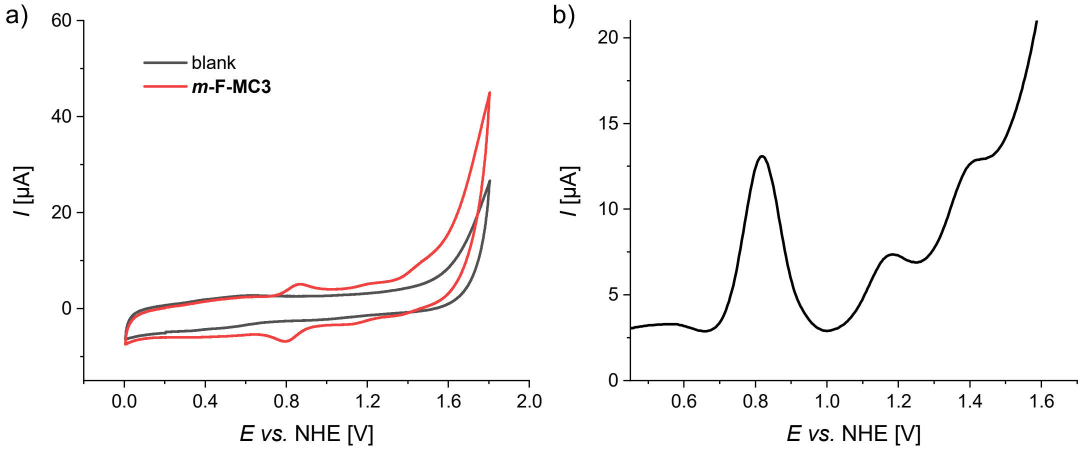

Fig. S4 CV (a) and DPV (b) of ***m*-F-MC3** in TFE/H_2O 1:1 (pH 1, triflic acid), *c* = 0.25 mM.

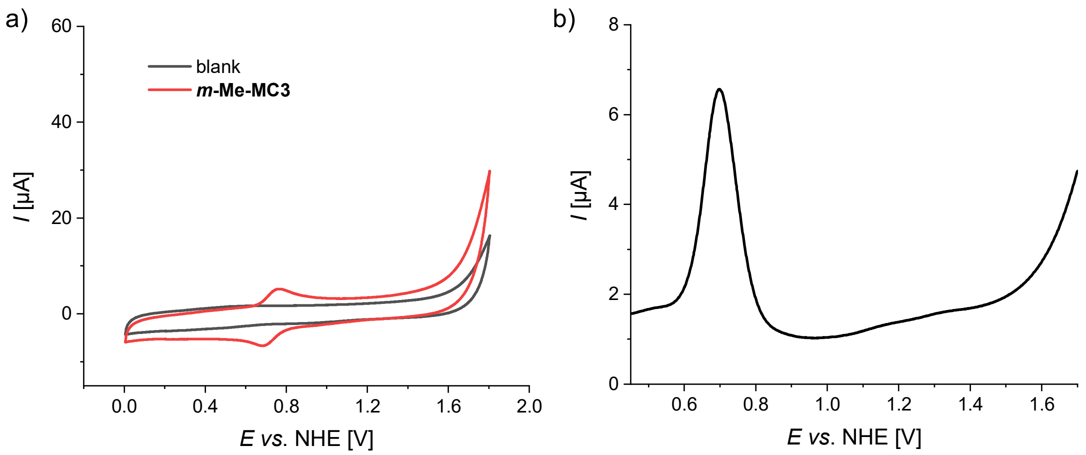

Fig. S5 CV (a) and DPV (b) of ***m*-Me-MC3** in TFE/H_2O 1:1 (pH 1, triflic acid), *c* = 0.25 mM.

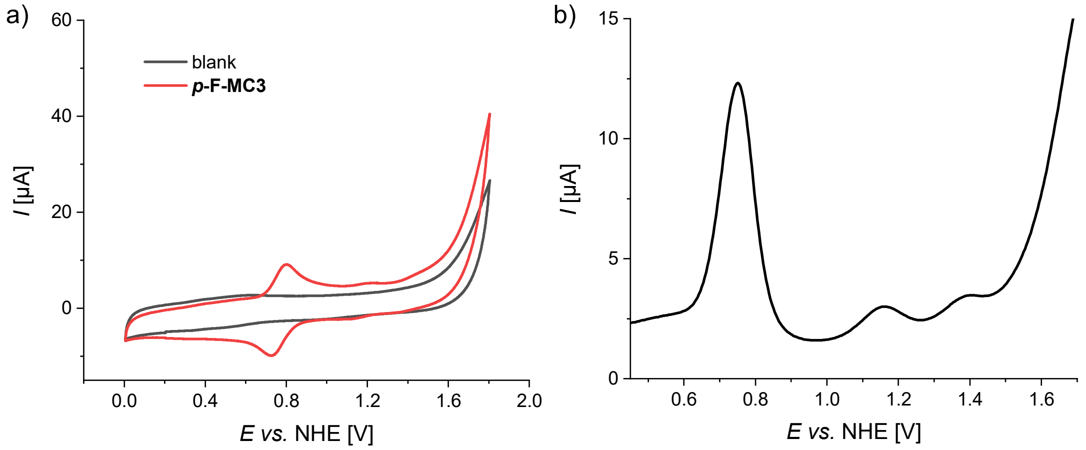

Fig. S6 CV (a) and DPV (b) of ***p*-F-MC3** in TFE/H_2O 1:1 (pH 1, triflic acid), *c* = 0.25 mM.

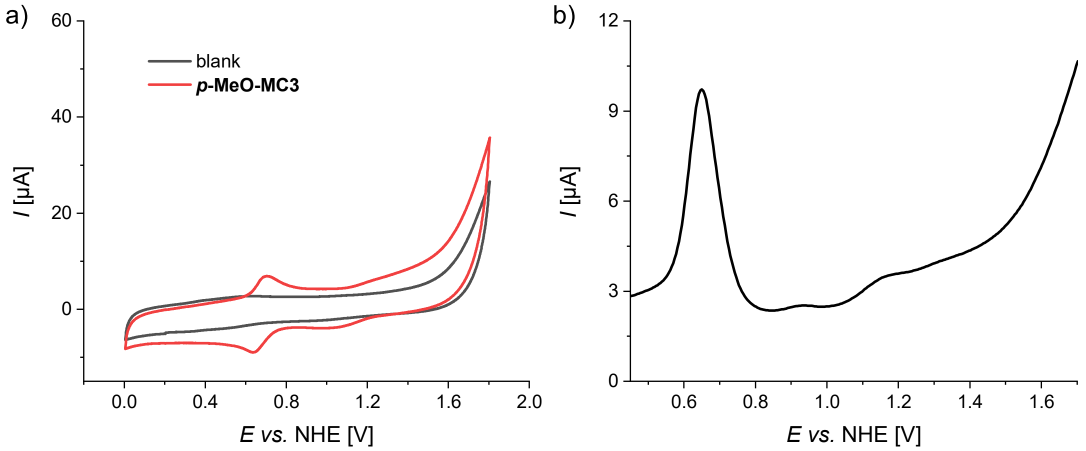

Fig. S7 CV (a) and DPV (b) of ***p*-MeO-MC3** in TFE/H_2O 1:1 (pH 1, triflic acid), *c* = 0.25 mM.

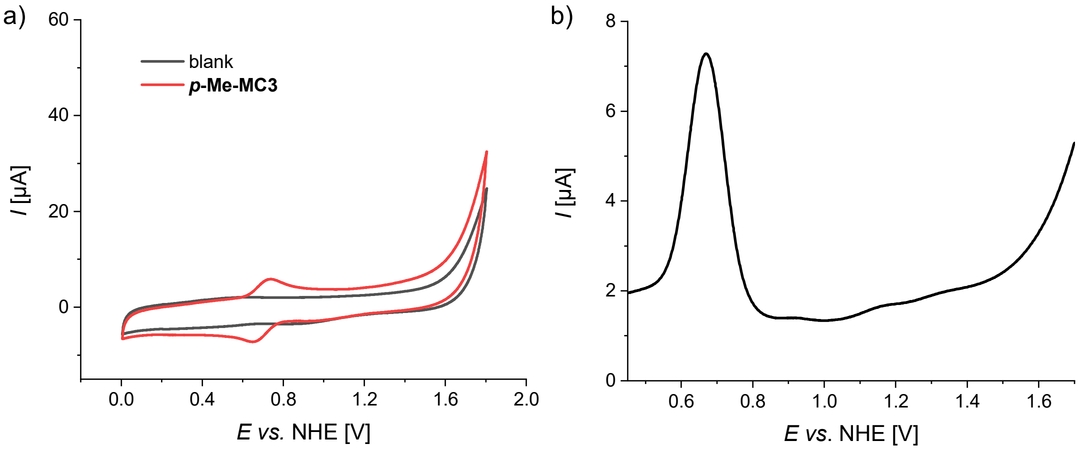

Fig. S8 CV (a) and DPV (b) of ***p*-Me-MC3** in TFE/H_2O 1:1 (pH 1, triflic acid), *c* = 0.25 mM.

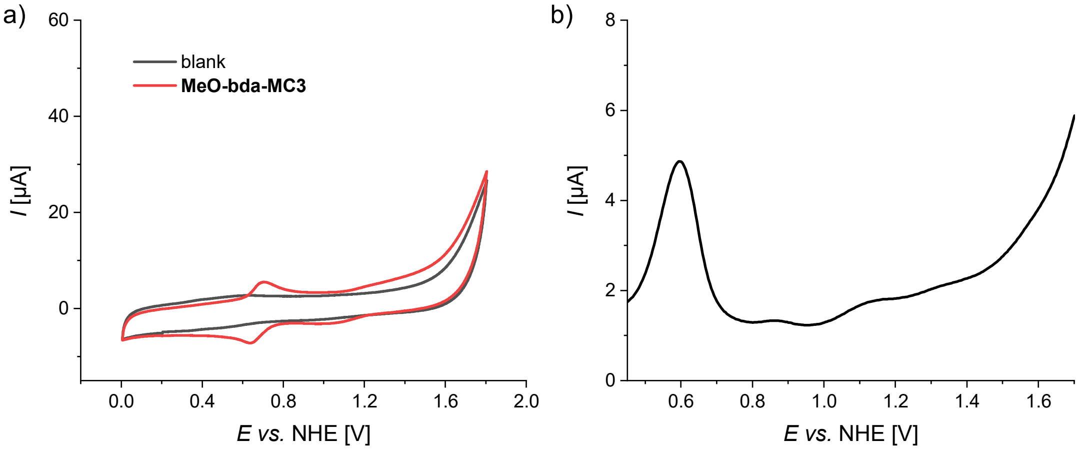

Fig. S9 CV (a) and DPV (b) of **MeO-bda-MC3** in TFE/H_2O 1:1 (pH 1, triflic acid), *c* = 0.25 mM.

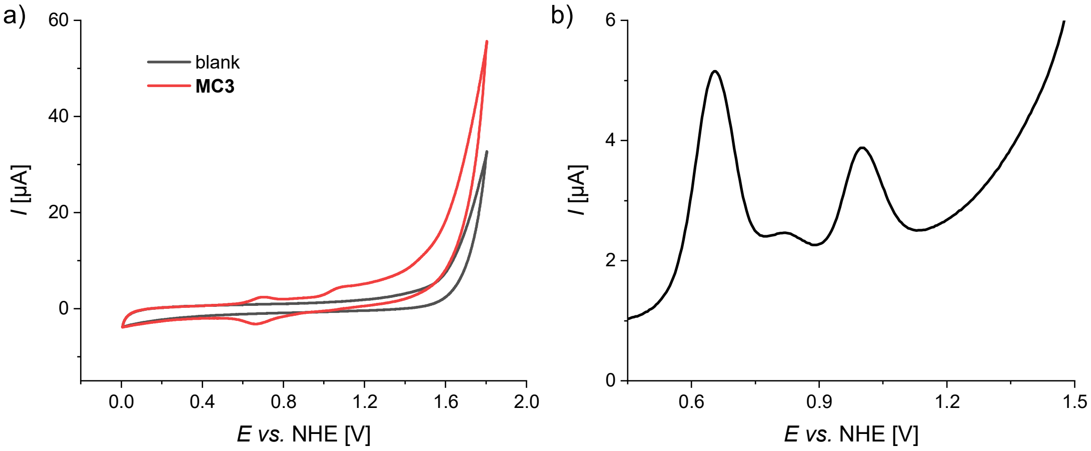

Fig. S10 CV (a) and DPV (b) of **MC3** in TFE/H_2O 1:1 (pH 7, phosphate buffer), *c* = 0.25 mM.

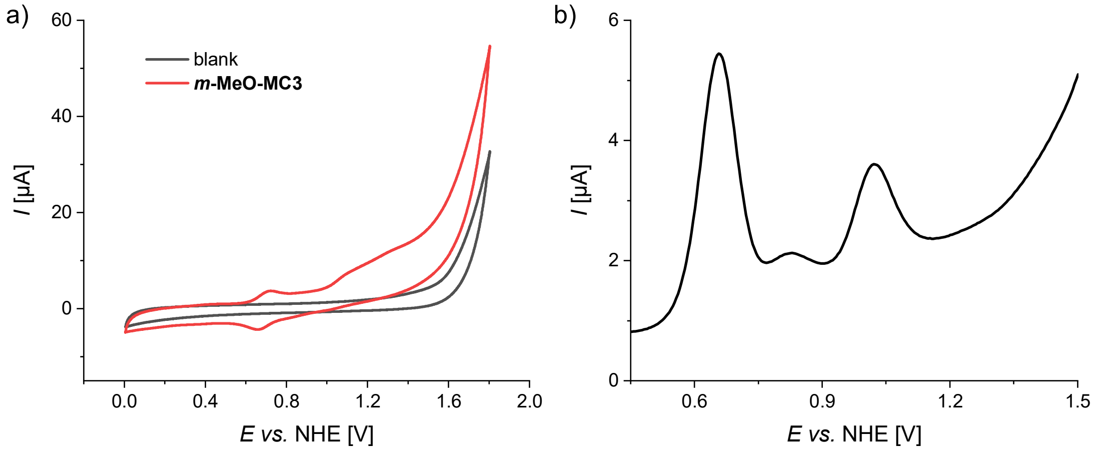

Fig. S11 CV (a) and DPV (b) of ***m*-MeO-MC3** in TFE/H_2O 1:1 (pH 7, phosphate buffer), *c* = 0.25 mM.

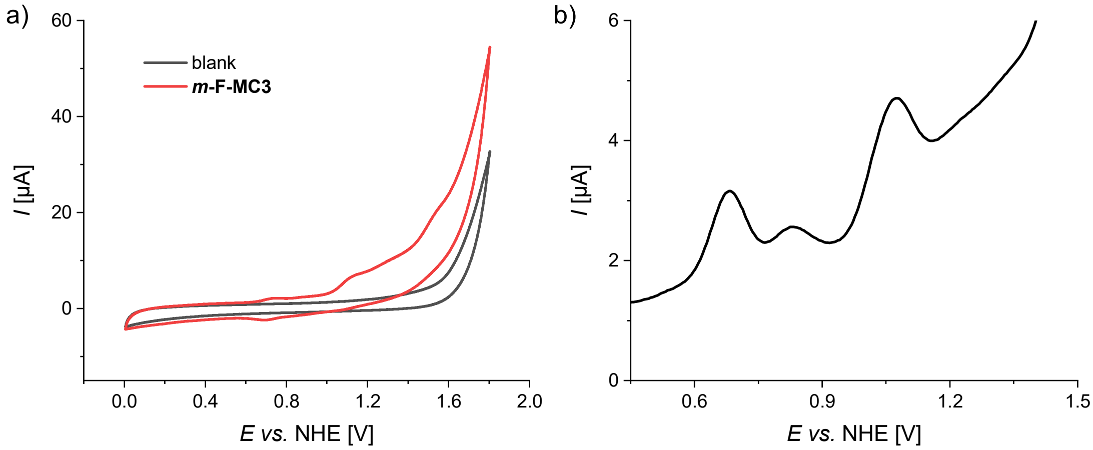

Fig. S12 CV (a) and DPV (b) of ***m*-F-MC3** in TFE/H_2O 1:1 (pH 7, phosphate buffer), *c* = 0.25 mM.

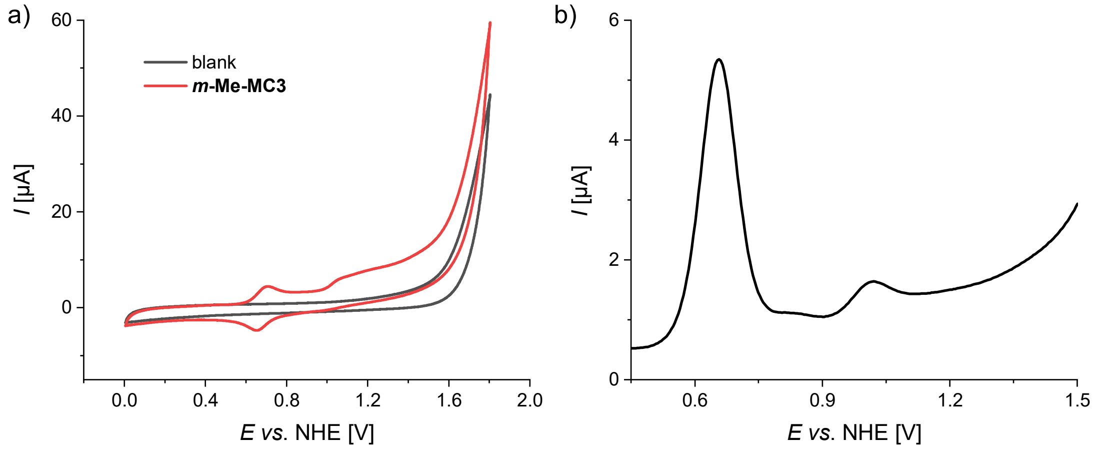

Fig. S13 CV (a) and DPV (b) of ***m*-Me-MC3** in TFE/H_2O 1:1 (pH 7, phosphate buffer), *c* = 0.25 mM.

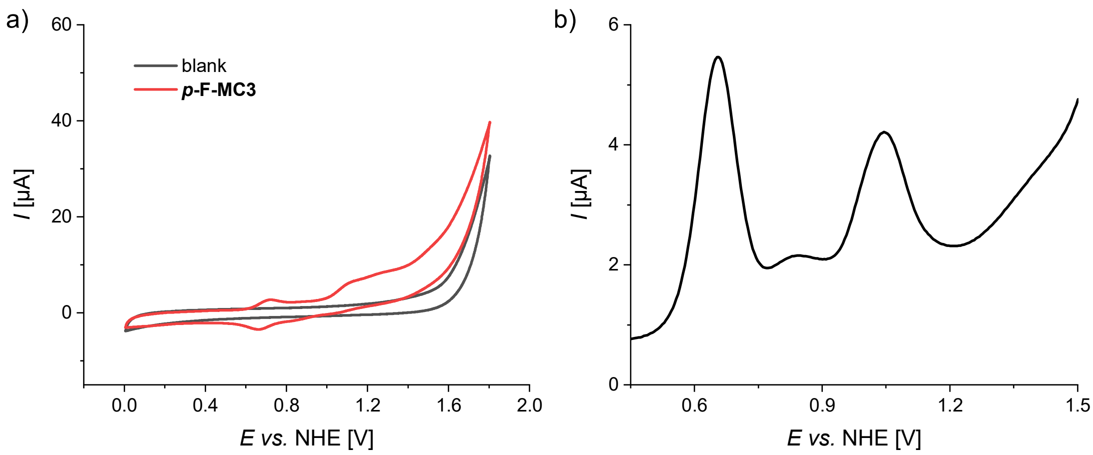

Fig. S14 CV (a) and DPV (b) of ***p*-F-MC3** in TFE/H_2O 1:1 (pH 7, phosphate buffer), *c* = 0.25 mM.

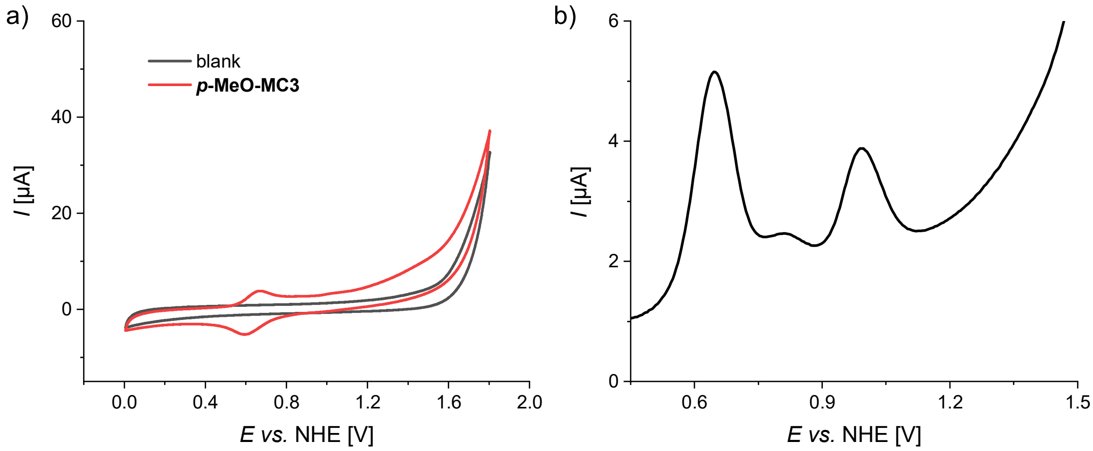

Fig. S15 CV (a) and DPV (b) of ***p*-MeO-MC3** in TFE/H_2O 1:1 (pH 7, phosphate buffer), *c* = 0.25 mM.

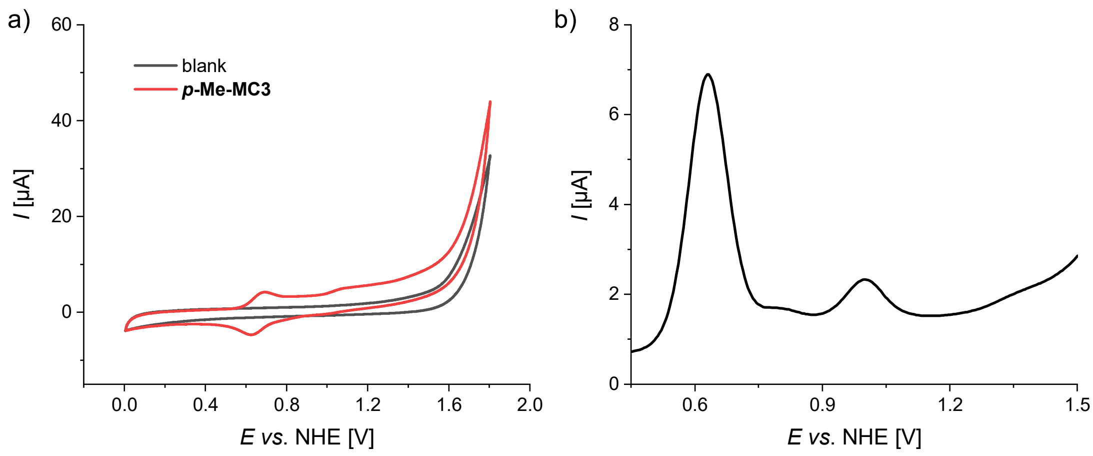

Fig. S16 CV (a) and DPV (b) of ***p*-Me-MC3** in TFE/H_2O 1:1 (pH 7, phosphate buffer), *c* = 0.25 mM.

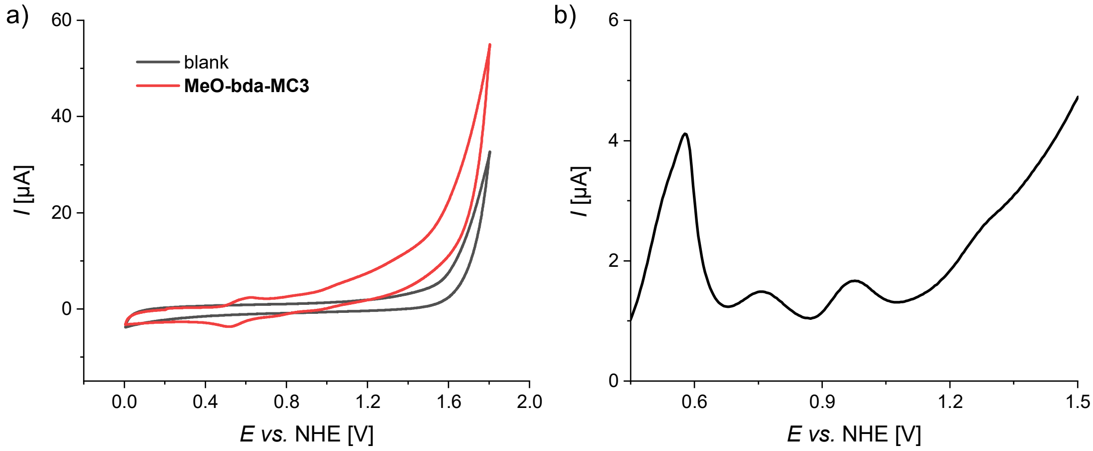

Fig. S17 CV (a) and DPV (b) of **MeO-bda-MC3** in TFE/H_2O 1:1 (pH 7, phosphate buffer), c = 0.25 mM.

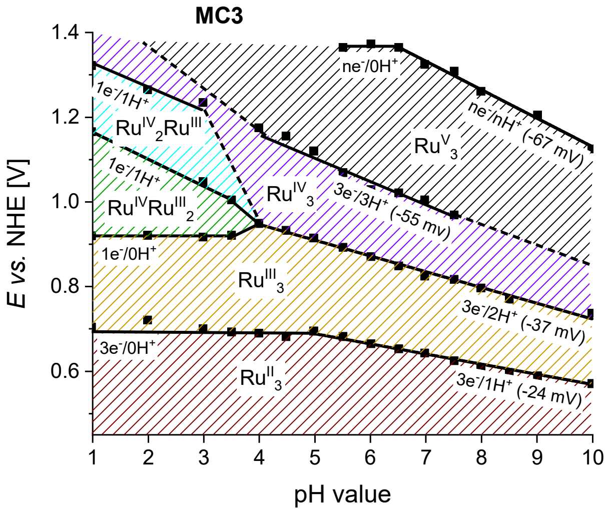

Fig. S18. Pourbaix diagram of **MC3**. DPV measurements were performed in TFE/H_2O 1:1 (phosphate buffer at different pH values). In a previous publication (ref. [S11]) a different Pourbaix diagram of **MC3** was published. The discrepancy is presumably due to some differences in experimental procedure. In ref. [S11], the pH value of a starting acidic solution of the macrocycle was adjusted by successive addition of sodium hydroxide disregarding any control over the ionic strength. Here, several solutions of **MC3** were prepared at a constant ionic strength of 0.1 M.

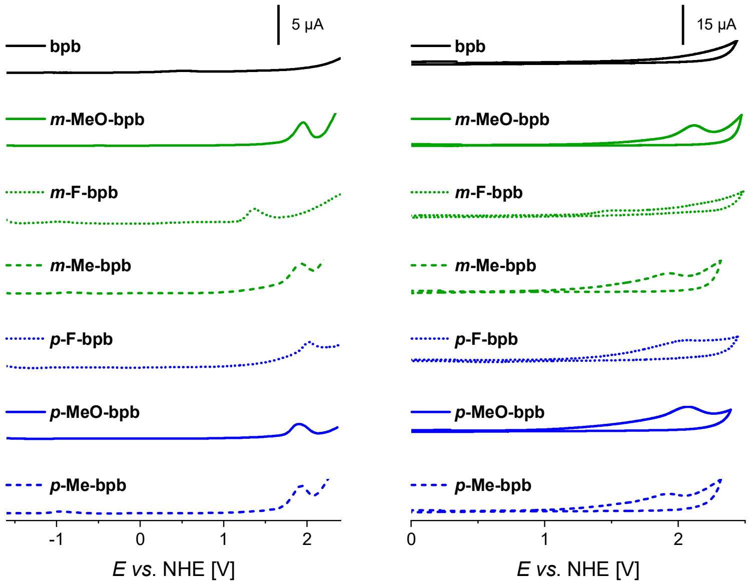

Fig. S19 CV (left) and DPV (right) of functionalized bpb bridging ligands in DCM, $^{n}Bu_4NPF_6$ (0.1 M), *c* = 0.25 mM.

5. UV/Vis absorption spectroscopy and spectroelectrochemistry

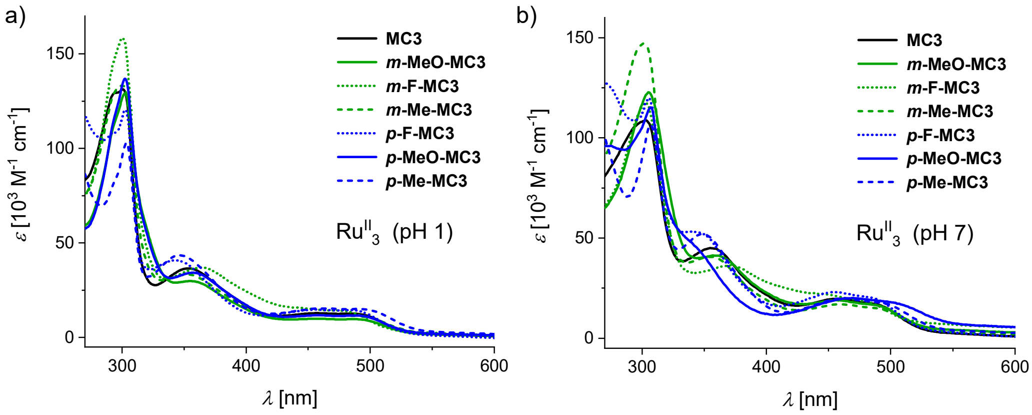

Fig. 20 UV/Vis absorption spectra of the macrocyclic **MC3** derivatives at the Ru^{II}_3 state in MeCN/H_2O 1:1 at pH 1 (a) and pH 7 (b), *c* = 10 µM.

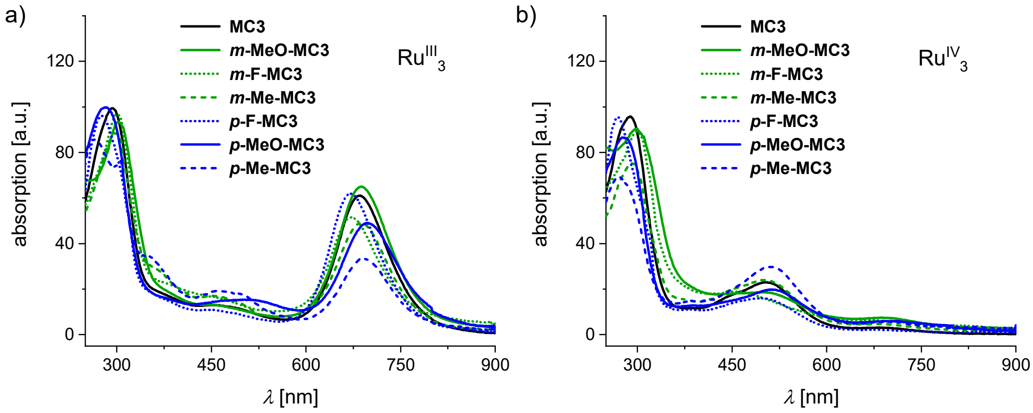

Fig. S21 UV/Vis absorption spectra of the macrocyclic **MC3** derivatives at the Ru^{III}_3 (a) and Ru^{IV}_3 (b) states in MeCN/H_2O 1:1 (phosphate buffer, pH 7), *c* = 0.24 mM. Oxidized Ru species were generated by application of an increasing voltage from 500 mV to 1100 mV.

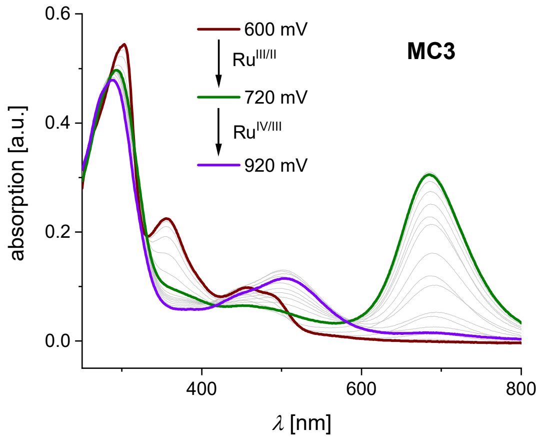

Fig S22 Spectroelectrochemistry of **MC3** in MeCN/H2O 1:1 (phosphate buffer, pH 7), *c* = 0.24 mM. Starting from $Ru^{II}{}_3$ (brown), $Ru^{III}{}_3$ (green) and $Ru^{IV}{}_3$ (purple) species were generated by application of an increasing voltage as indicated in the inset.

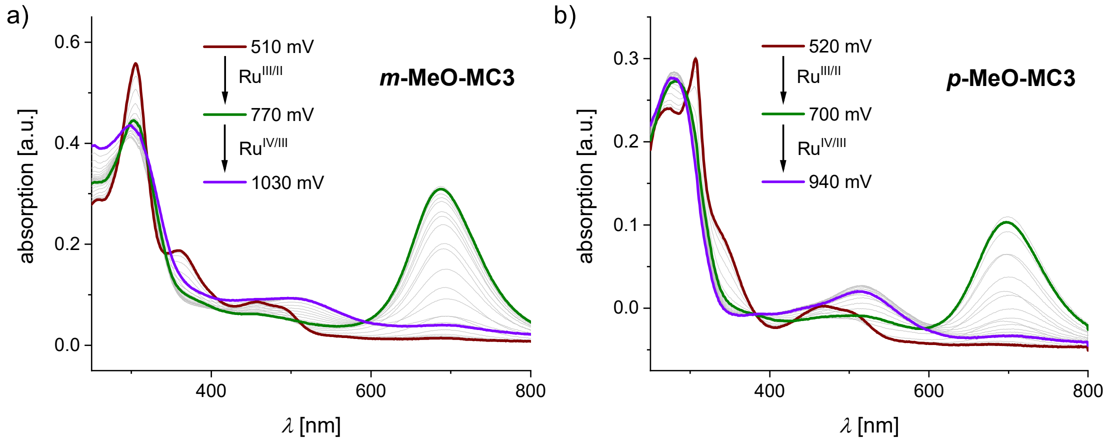

Fig. S23 Spectroelectrochemistry of (a) ***m*-MeO-MC3** and (b) ***p*-MeO-MC3** in MeCN/H2O 1:1 (phosphate buffer, pH 7), *c* = 0.24 mM. Starting from $Ru^{II}{}_3$ (brown), $Ru^{III}{}_3$ (green) and $Ru^{IV}{}_3$ (purple) species were generated by application of an increasing voltage as indicated in the insets.

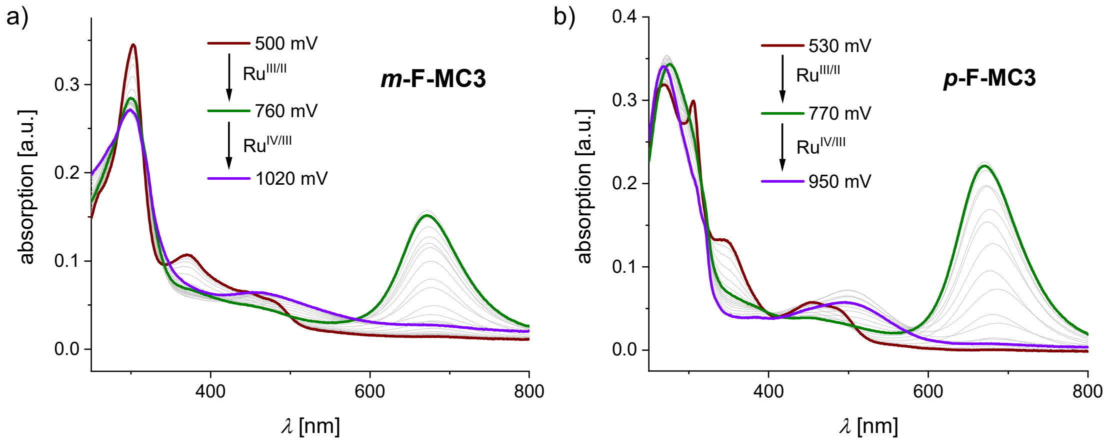

Fig. S24 Spectroelectrochemistry of (a) ***m*-F-MC3** and (b) ***p*-F-MC3** in MeCN/H2O 1:1 (phosphate buffer, pH 7), *c* = 0.24 mM. Starting from $Ru^{II}{}_3$ (brown), $Ru^{III}{}_3$ (green) and $Ru^{IV}{}_3$ (purple) species were generated by application of an increasing voltage as indicated in the insets.

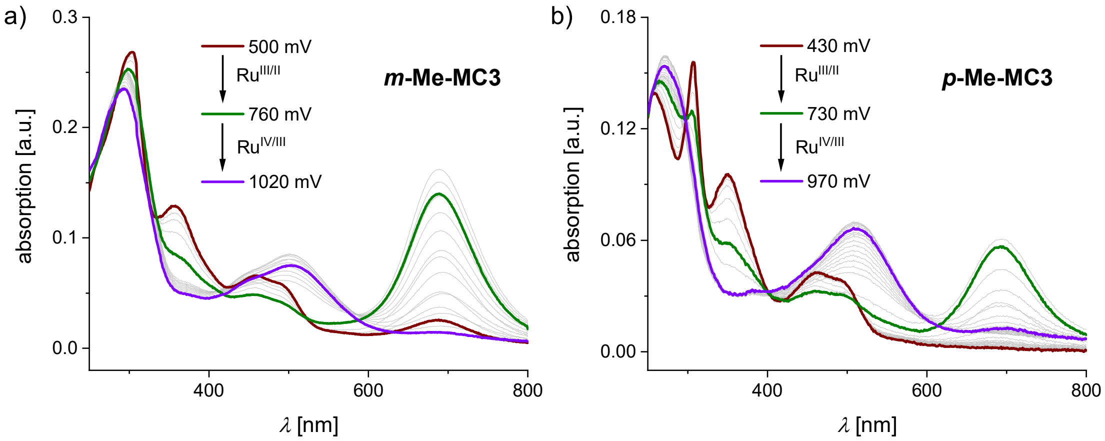

Fig. S25 Spectroelectrochemistry of (a) ***m*-Me-MC3** and (b) ***p*-Me-MC3** in MeCN/H_2O 1:1 (phosphate buffer, pH 7), c = 0.24 mM. Starting from Ru^{II}_3 (brown), Ru^{III}_3 (green) and Ru^{IV}_3 (purple) species were generated by application of an increasing voltage as indicated in the insets.

6. Chemical water oxidation

Table S3 Catalytic activity of **MC3** derivatives in chemical water oxidation with varying MeCN content.[a]

MeCN content	**MC3**		***m*-MeO-MC3**		***m*-F-MC3**		***m*-Me-MC3**	
	TOF [s^{-1}]	TON	TOF [s^{-1}]	TON	TOF [s^{-1}]	TON	TOF [s^{-1}]	TON
30%	23	1600	21	600	24	1500	10	400
40%	102	3100	127	750	87	2150	60	2300
50%	136	5300	138	1300	90	4700	90	3700
60%	150	7400	102	3900	84	4500	60	3000
70%	72	5200	104	1300	44	550	40	2600

MeCN content	***p*-F-MC3**		***p*-MeO-MC3**		***p*-Me-MC3**	
	TOF [s^{-1}]	TON	TOF [s^{-1}]	TON	TOF [s^{-1}]	TON
30%	18	250	22	450	26	650
40%	51	900	44	1200	37	1900
50%	80	2500	60	2200	60	2300
60%	64	3150	20	1800	20	1100
70%	38	2550	17	1000	6	600

[a] Experiments in MeCN/H_2O mixtures (pH 1, triflic acid), c(CAN) = 0.6 M, c(WOC) = 24 µM.

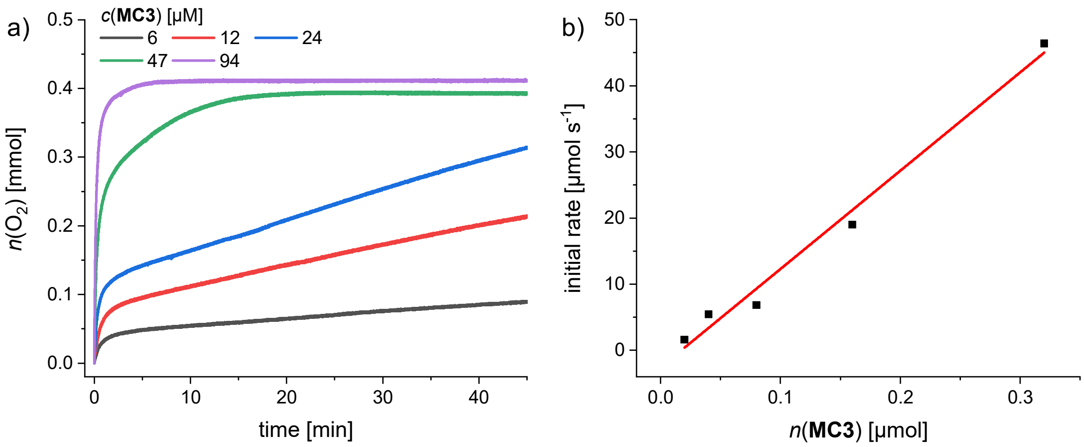

Fig. S26 a) Concentration-dependent experiments with **MC3** as WOC in MeCN/H_2O 1:1 (pH 1, triflic acid). b) Plot of initial rates *vs.* catalyst amount with linear regression for the determination of TOF.

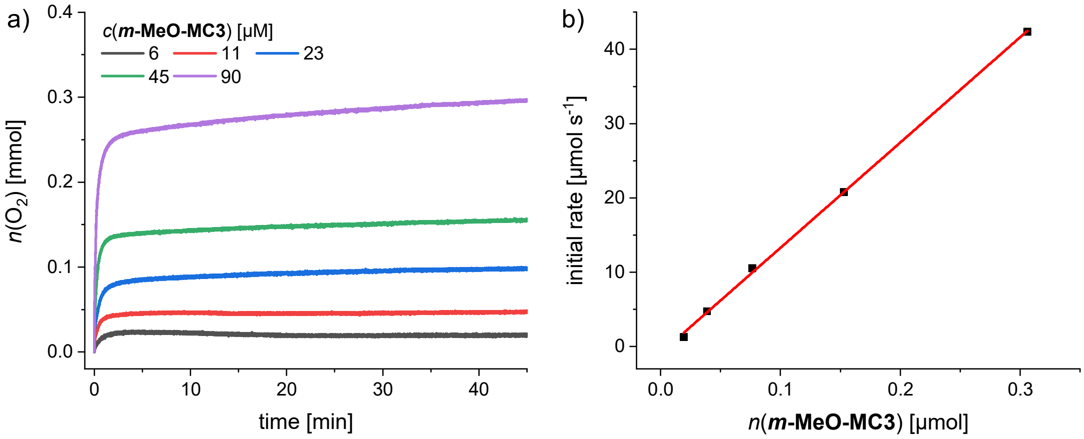

Fig. S27 a) Concentration-dependent experiments with ***m*-MeO-MC3** as WOC in MeCN/H_2O 1:1 (pH 1, triflic acid). b) Plot of initial rates *vs.* catalyst amount with linear regression for the determination of TOF.

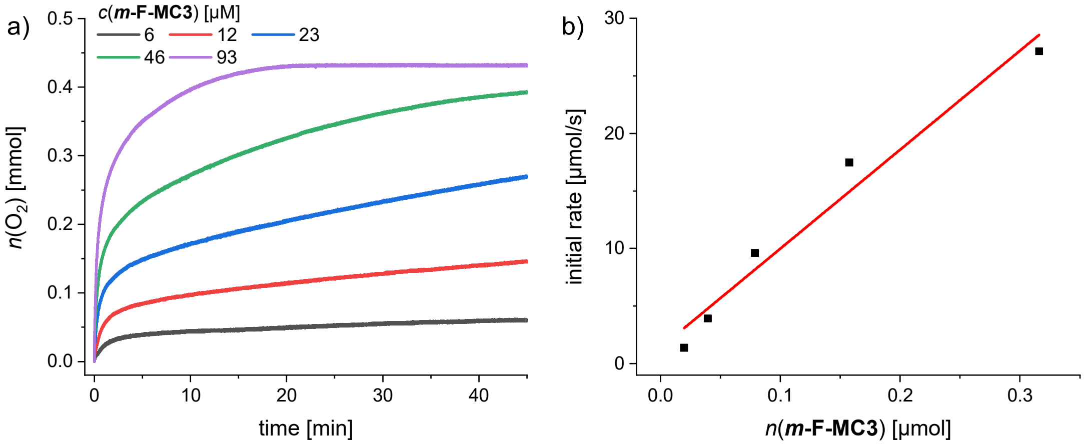

Fig. S28 a) Concentration-dependent experiments with ***m*-F-MC3** as WOC in MeCN/H_2O 1:1 (pH 1, triflic acid). b) Plot of initial rates *vs.* catalyst amount with linear regression for the determination of TOF.

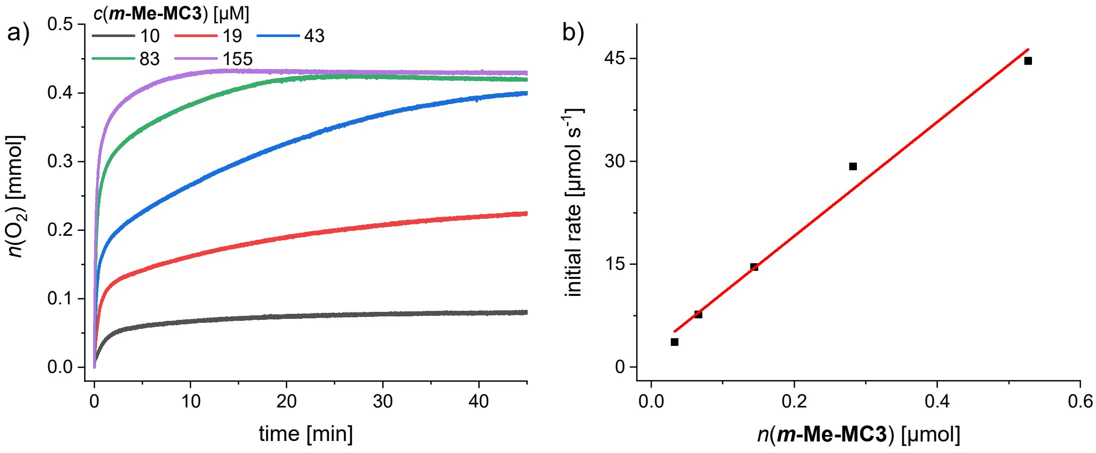

Fig. S29 a) Concentration-dependent experiments with ***m*-Me-MC3** as WOC in MeCN/H_2O 1:1 (pH 1, triflic acid). b) Plot of initial rates *vs.* catalyst amount with linear regression for the determination of TOF.

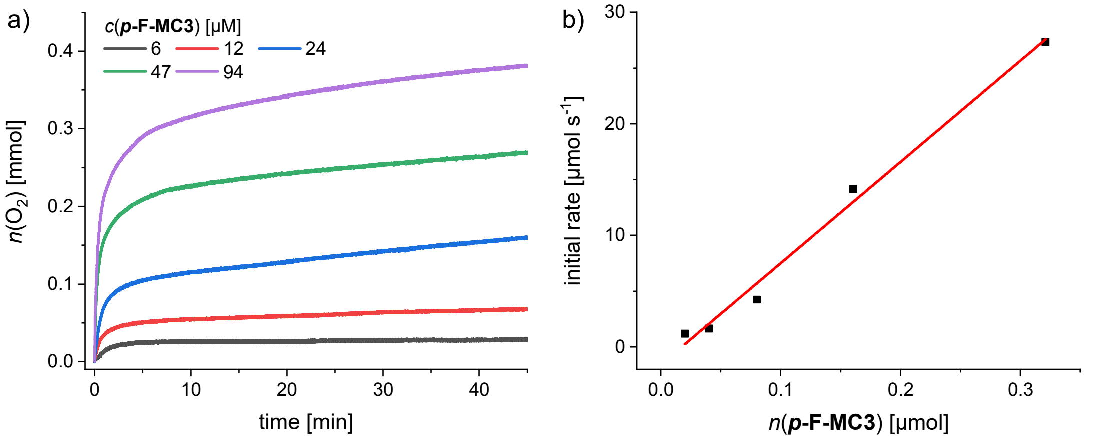

Fig. S30 a) Concentration-dependent experiments with ***p*-F-MC3** as WOC in MeCN/H_2O 1:1 (pH 1, triflic acid). b) Plot of initial rates *vs.* catalyst amount with linear regression for the determination of TOF.

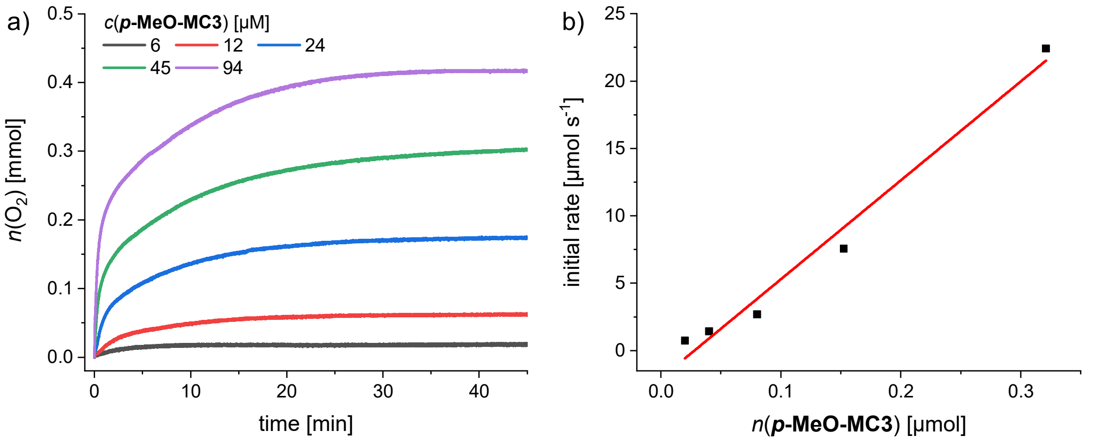

Fig. S31 a) Concentration-dependent experiments with ***p*-MeO-MC3** as WOC in MeCN/H_2O 1:1 (pH 1, triflic acid). b) Plot of initial rates *vs.* catalyst amount with linear regression for the determination of TOF.

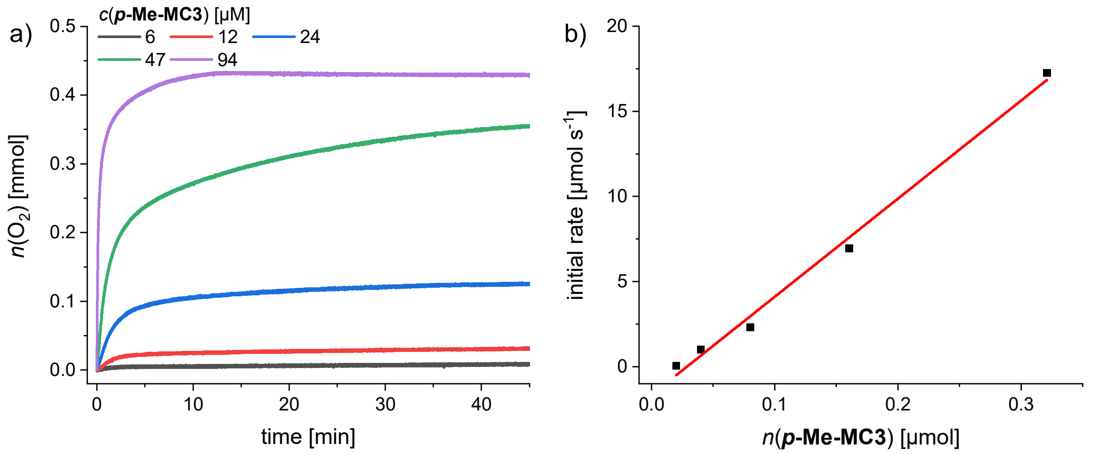

Fig. S32 a) Concentration-dependent experiments with ***p*-Me-MC3** as WOC in MeCN/H_2O 1:1 (pH 1, triflic acid). b) Plot of initial rates *vs.* catalyst amount with linear regression for the determination of TOF.

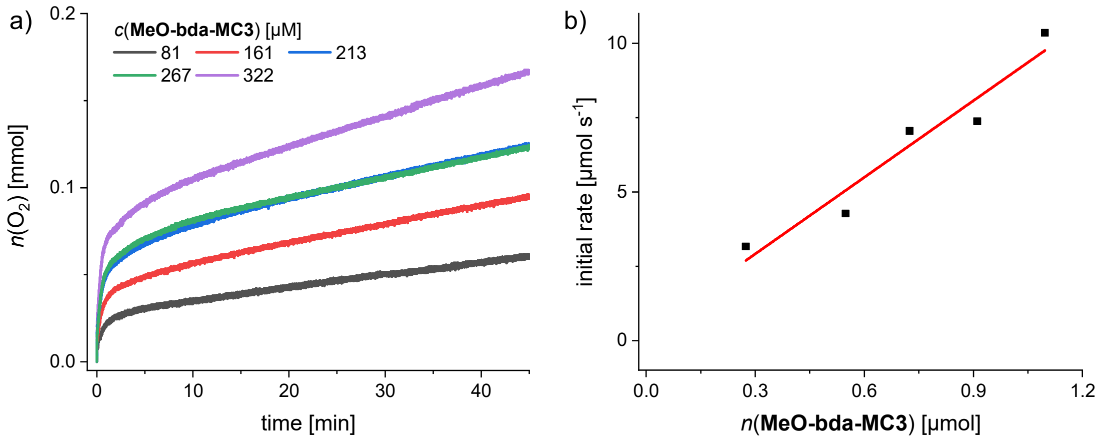

Fig. S33 a) Concentration-dependent experiments with **MeO-bda-MC3** as WOC in MeCN/H_2O 1:1 (pH 1, triflic acid). b) Plot of initial rates *vs.* catalyst amount with linear regression for the determination of TOF.

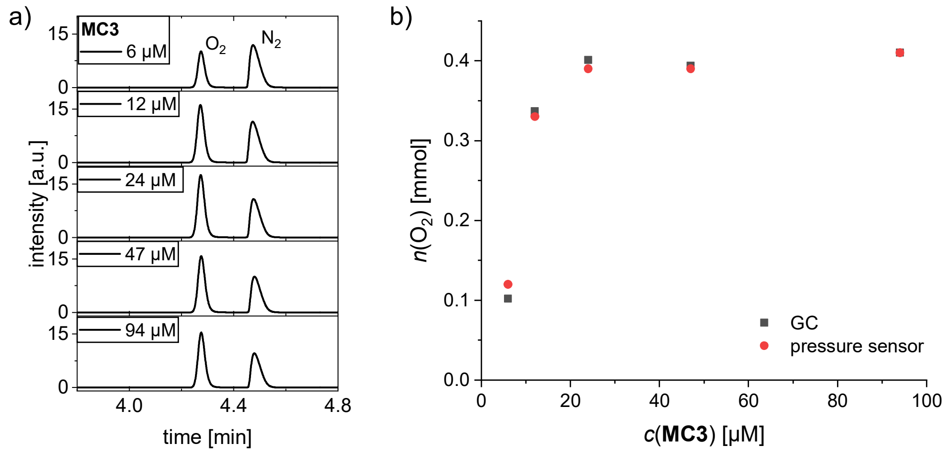

Fig. S34 a) Chromatogram of headspace at the end of water oxidation experiments with **MC3** as WOC. b) Comparison of amount of evolved oxygen determined by GC or with pressure sensors.

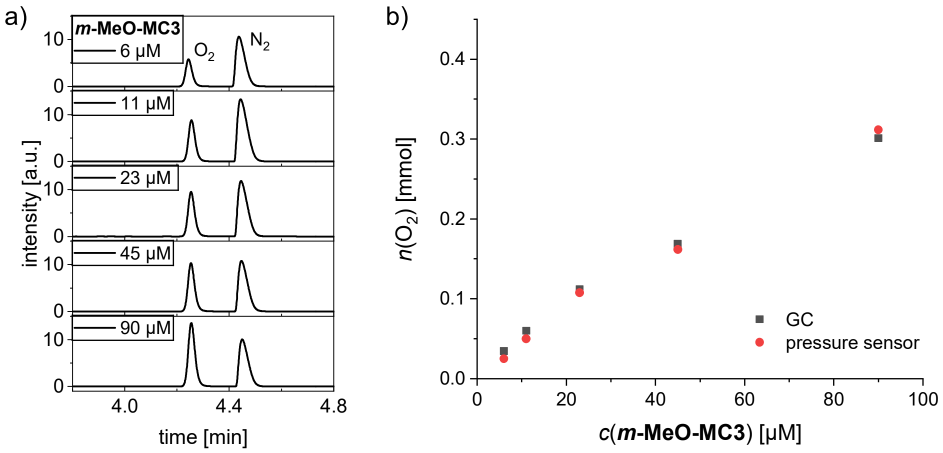

Fig. S35 a) Chromatogram of headspace at the end of water oxidation experiments with ***m*-MeO-MC3** as WOC. b) Comparison of amount of evolved oxygen determined by GC or with pressure sensors.

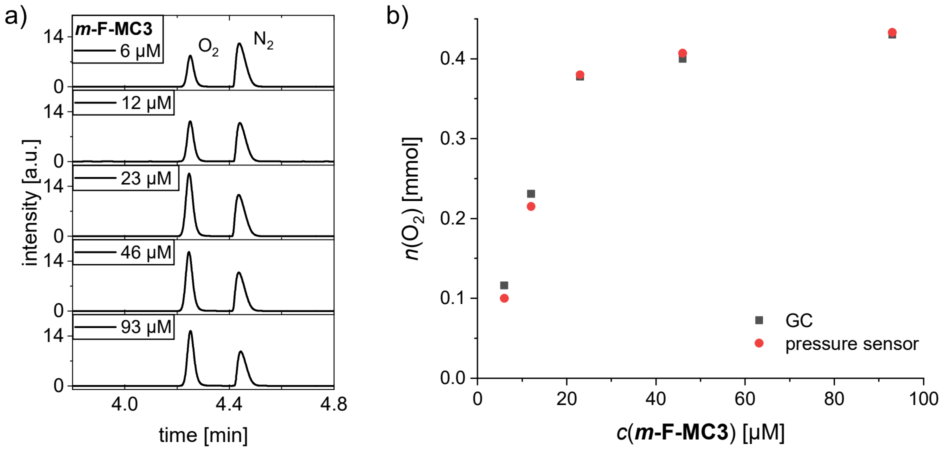

Fig. S36 a) Chromatogram of headspace at the end of water oxidation experiments with ***m*-F-MC3** as WOC. b) Comparison of amount of evolved oxygen determined by GC or with pressure sensors.

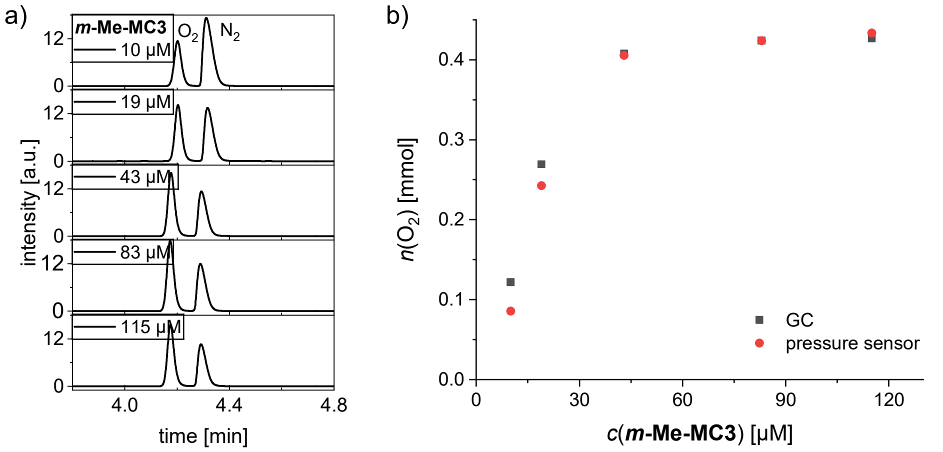

Fig. S37 a) Chromatogram of headspace at the end of water oxidation experiments with ***m*-Me-MC3** as WOC. b) Comparison of amount of evolved oxygen determined by GC or with pressure sensors.

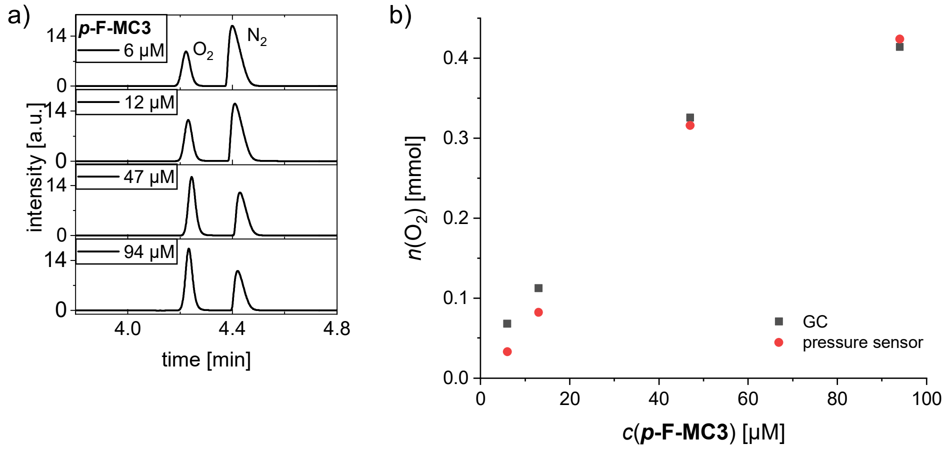

Fig. S38 a) Chromatogram of headspace at the end of water oxidation experiments with ***p*-F-MC3** as WOC. b) Comparison of amount of evolved oxygen determined by GC or with pressure sensors.

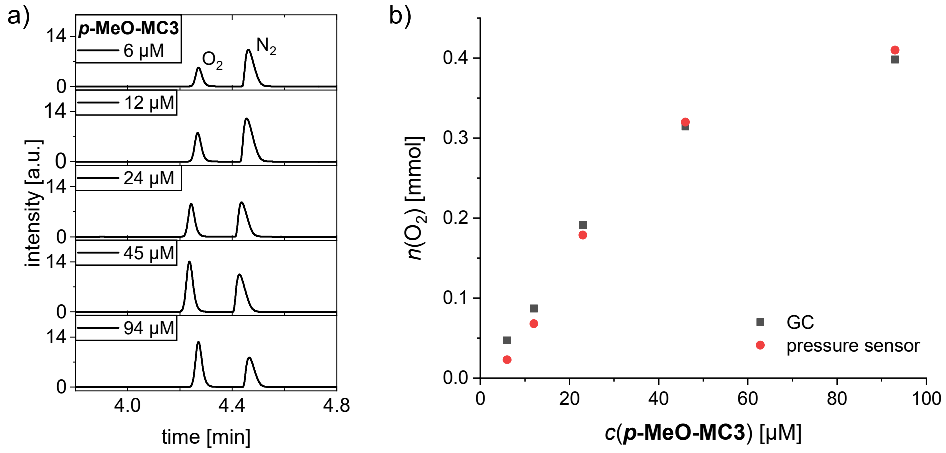

Fig. S39 a) Chromatogram of headspace at the end of water oxidation experiments with ***p*-MeO-MC3** as WOC. b) Comparison of amount of evolved oxygen determined by GC or with pressure sensors.

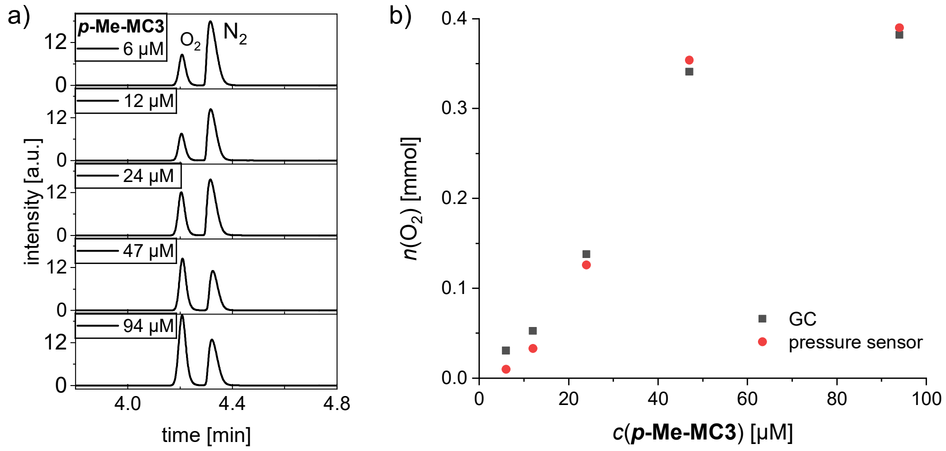

Fig. S40 a) Chromatogram of headspace at the end of water oxidation experiments with ***p*-Me-MC3** as WOC. b) Comparison of amount of evolved oxygen determined by GC or with pressure sensors.

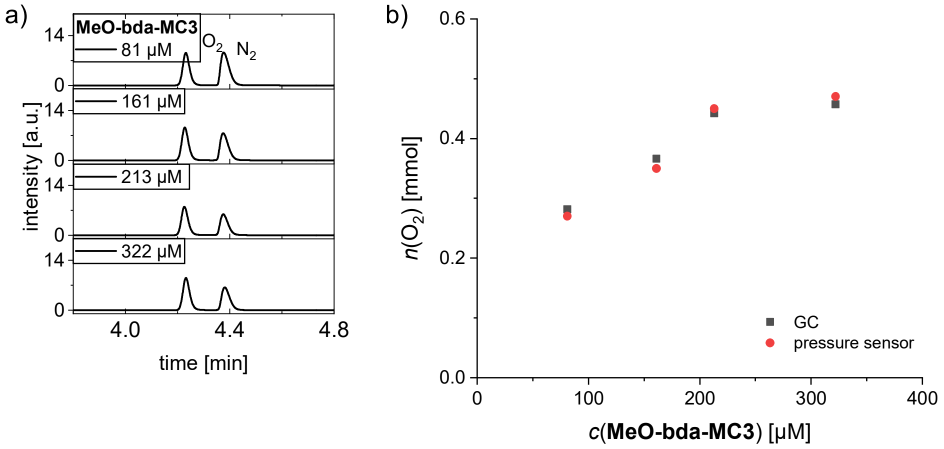

Fig. S41 a) Chromatogram of headspace at the end of water oxidation experiments with **MeO-bda-MC3** as WOC. b) Comparison of amount of evolved oxygen determined by GC or with pressure sensors.

7. Photocatalytic water oxidation

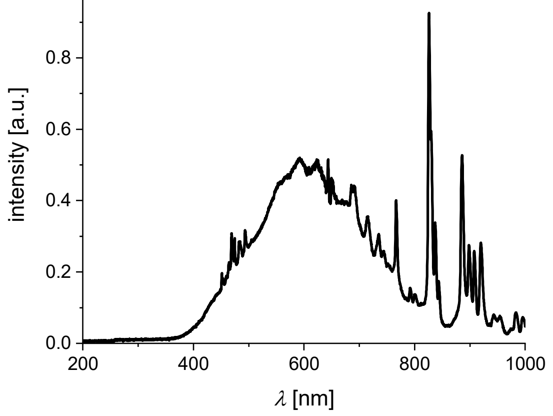

Fig. S42 Emission profile of xenon lamp used for photocatalytic water oxidation experiments.

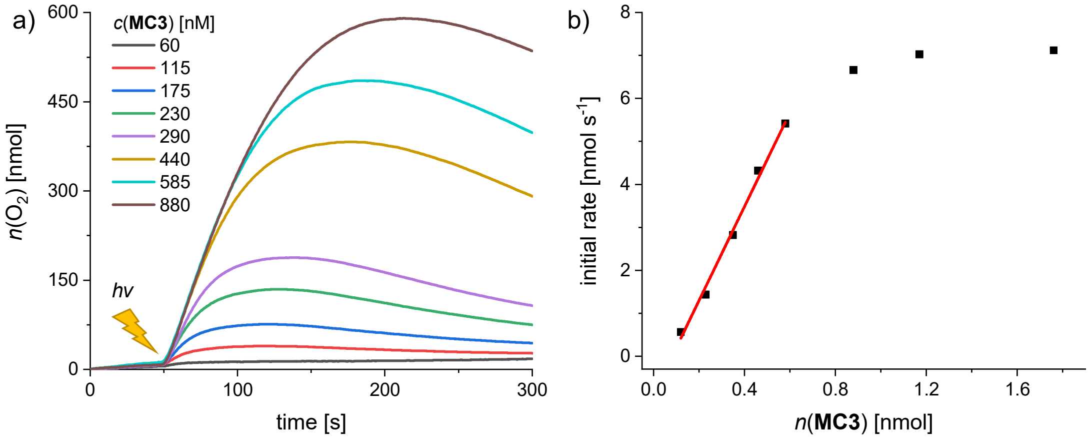

Fig. S43 a) Concentration-dependent experiments with **MC3** as WOC in MeCN/H_2O 1:1 (phosphate buffer, pH 7). b) Plot of initial rates *vs.* catalyst amount with linear regression for the determination of TOF.

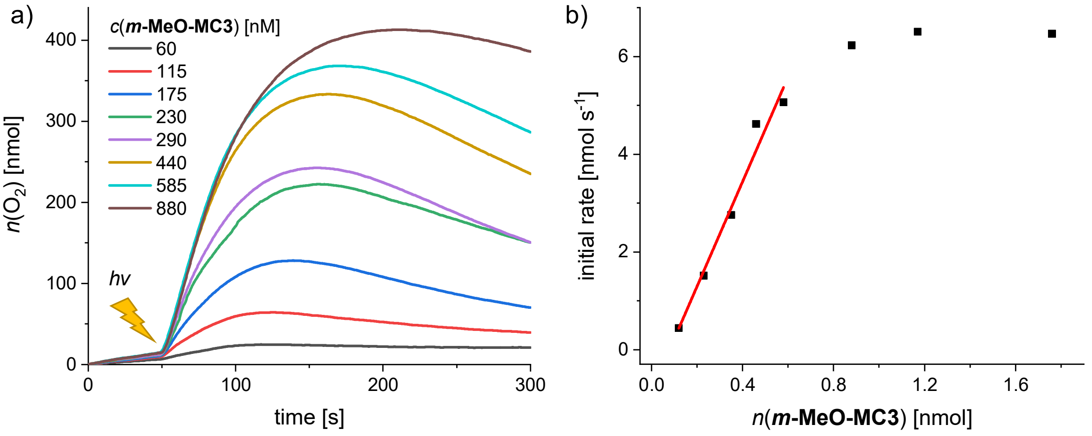

Fig. S44 a) Concentration-dependent experiments with ***m*-MeO-MC3** as WOC in MeCN/H_2O 1:1 (phosphate buffer, pH 7). b) Plot of initial rates *vs.* catalyst amount with linear regression for the determination of TOF.

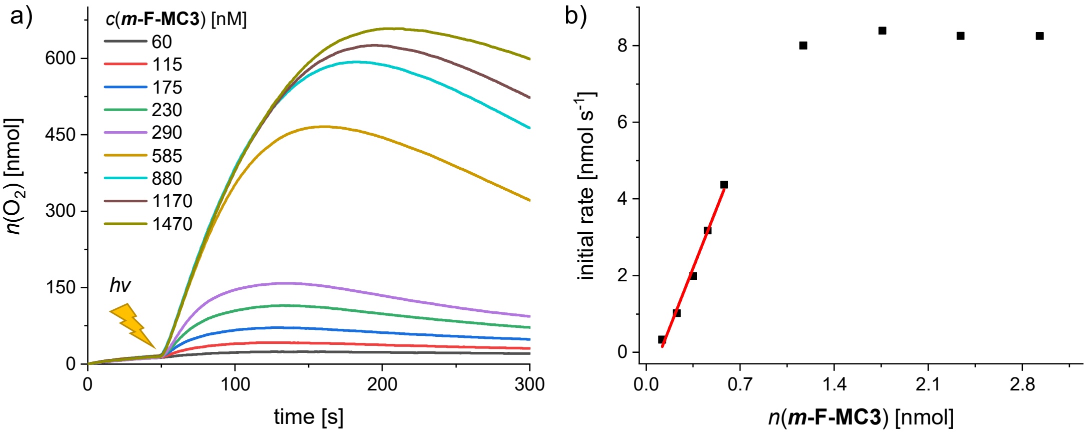

Fig. S45 a) Concentration-dependent experiments with ***m*-F-MC3** as WOC in MeCN/H_2O 1:1 (phosphate buffer, pH 7). b) Plot of initial rates *vs.* catalyst amount with linear regression for the determination of TOF.

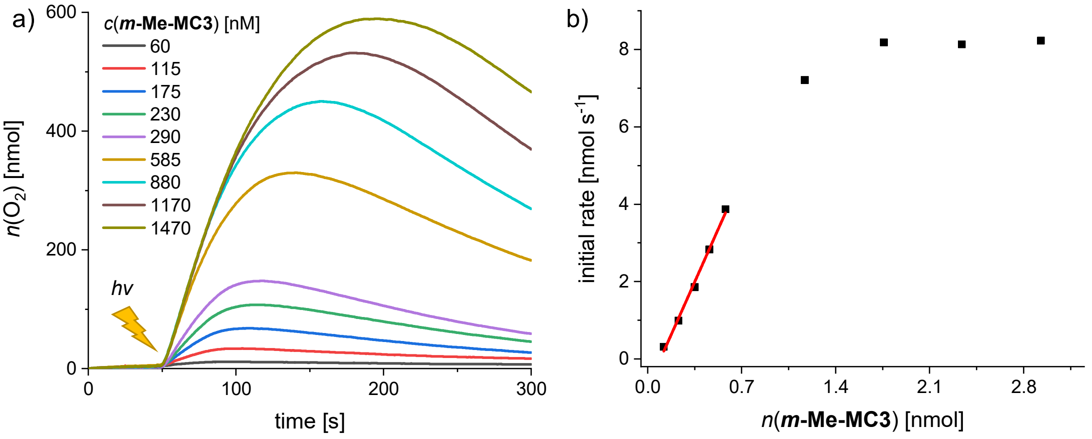

Fig. S46 a) Concentration-dependent experiments with ***m*-Me-MC3** as WOC in MeCN/H_2O 1:1 (phosphate buffer, pH 7). b) Plot of initial rates *vs.* catalyst amount with linear regression for the determination of TOF.

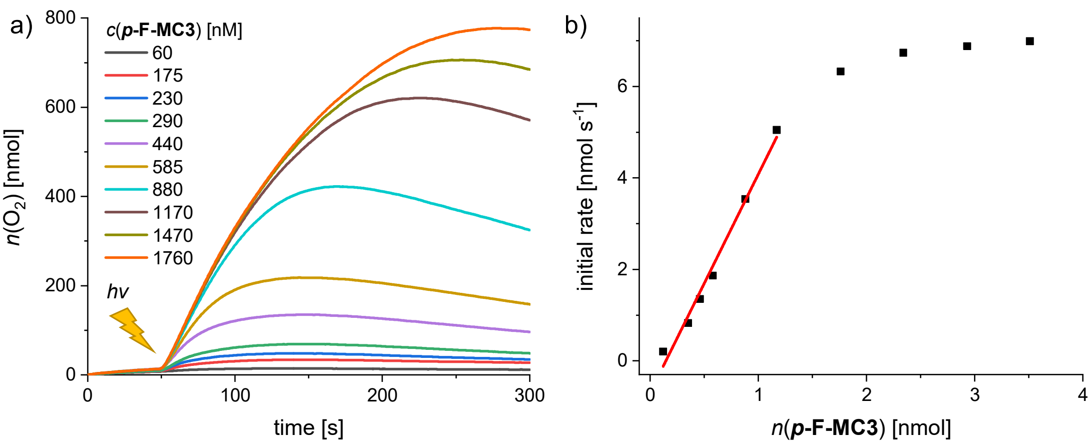

Fig. S47 a) Concentration-dependent experiments with ***p*-F-MC3** as WOC in MeCN/H_2O 1:1 (phosphate buffer, pH 7). b) Plot of initial rates *vs.* catalyst amount with linear regression for the determination of TOF.

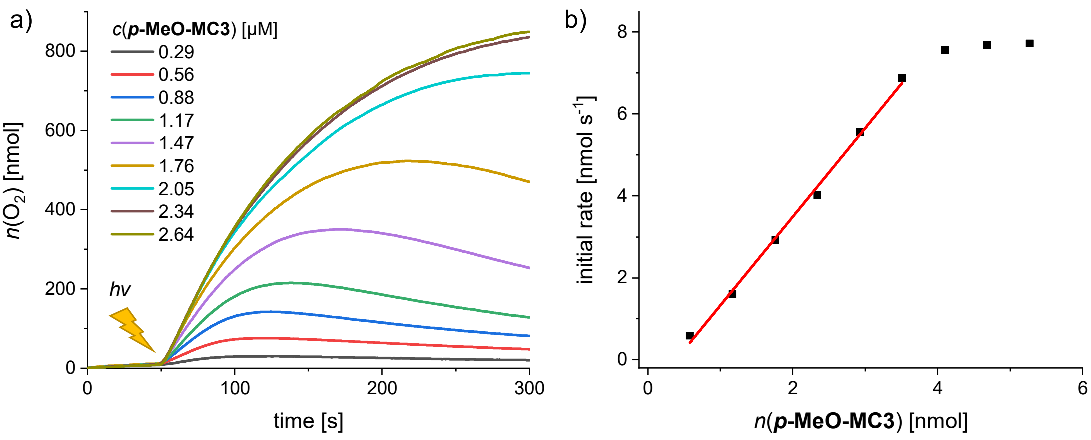

Fig. S48 a) Concentration-dependent experiments with ***p*-MeO-MC3** as WOC in MeCN/H_2O 1:1 (phosphate buffer, pH 7). b) Plot of initial rates *vs.* catalyst amount with linear regression for the determination of TOF.

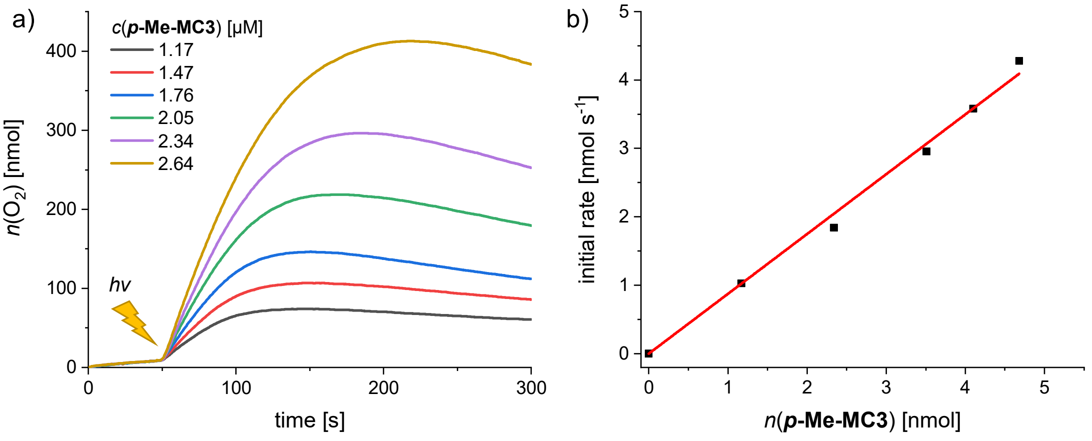

Fig. S49 a) Concentration-dependent experiments with ***p*-Me-MC3** as WOC in MeCN/H_2O 1:1 (phosphate buffer, pH 7). b) Plot of initial rates *vs.* catalyst amount with linear regression to determine TOF.

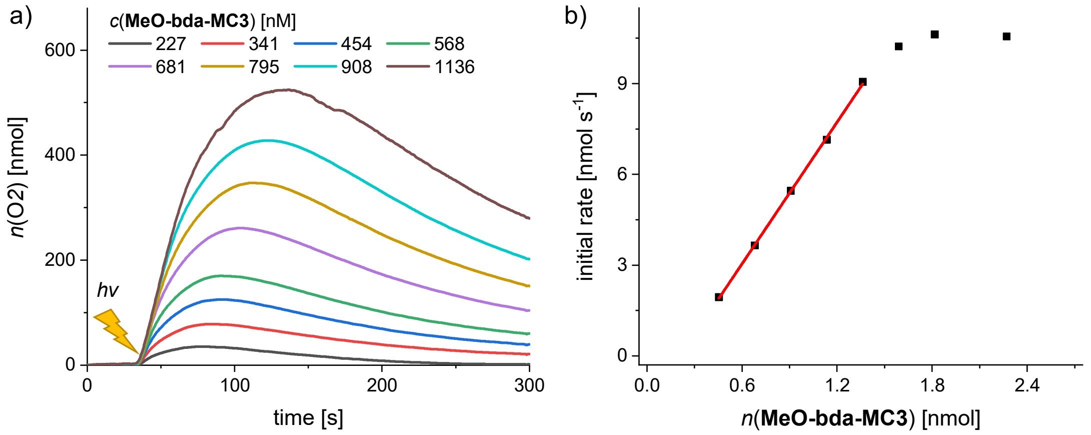

Fig. S50. Concentration-dependent experiments with **MeO-bda-MC3** as WOC in MeCN/H_2O 1:1 (phosphate buffer, pH 7). b) Plot of initial rates *vs.* catalyst amount with linear regression for the determination of TOF.

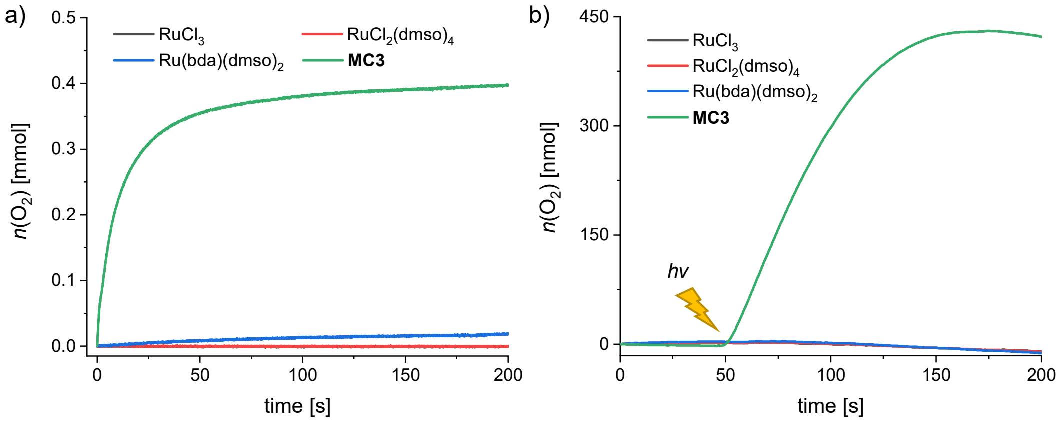

Fig S51. Catalytic activities of $RuCl_3$, $RuCl_2(dmso)_4$, $Ru(bda)(dmso)_2$ and **MC3** in a) chemical and b) photochemical water oxidation. Experimental conditions: a) MeCN/H_2O 1:1 (pH 1, triflic acid), *c*(CAN) = 0.6 M, *c*(**MC3**) = 94 µM, *c*($RuCl_3$) = *c*($RuCl_2(dmso)_4$) = *c*($Ru(bda)(dmso)_2$) = 282 µM. b) MeCN/H_2O (pH 7, phosphate buffer), *c*($[Ru(bpy)_3]^{2+}$) = 1.5 mM, *c*($Na_2S_2O_8$) = 37 mM, *c*(**MC3**) = 585 nM, *c*($RuCl_3$) = *c*($RuCl_2(dmso)_4$) = *c*($Ru(bda)(dmso)_2$) = 1755 nM. The lighting symbol indicates the start of sample irradiation at *t* = 50 s.

Table S4 Catalytic activities of selected Ru WOCs in chemical and photocatalytic water oxidation.

Catalyst	Chemical Water Oxidation (CAN)		Photocatalytic Water Oxidation ($[Ru(bpy)_3]^{2+}/Na_2S_2O_8$)	
	TOF_{max} [s^{-1}]	TON	TOF_{max} [s^{-1}]	TON
**MC3**[a]	136	5300	11	430
Ru(bda)(pic)$_2$ **1**[b] 28, 29	41	2000	0.35	10
Ru(bda)(isoq)$_2$ **2**[c] 30, 31	303	8360	0.24	140
[Ru(bda)(pic)]$_2$L^1 **3**[d] 32, 33	n.d.	20800	n.d.	640
**4**[e] 34, 35	0.068	211	11	5300
**5**[f] 36	inactive	inactive	50	1050
**6**[g] 37, 38	0.014	260	0.12	103
**7**[h] 39	0.001	5	n.d.	n.d.
**8**[i] 40	>0.001	320	n.d.	n.d.

Experimental conditions: [a] Chemical WO: 1:1 MeCN/H_2O (pH 1, triflic acid), *c*(CAN) = 0.6 M, *c*(**MC3**) = 5–322 µM; Photocatalytic WO: 1:1 MeCN/H_2O (pH 7, phosphate buffer), *c*($[Ru(bpy)_3]^{2+}$) = 1.5 mM, *c*($Na_2S_2O_8$) = 37 mM, *c*(**MC3**) = 60–600 nM. [b] Chemical WO:[28] H_2O (pH 1, triflic acid), *c*(CAN) = 0.4 M, *c*(**1**) = 12–216 µM; Photocatalytic WO:[29] H_2O (pH 7.2, phosphate buffer), *c*($[Ru(bpy)_3]^{2+}$) = 1 mM, *c*($Na_2S_2O_8$) = 10 mM, *c*(**1**) = 9.5 µM. [c] Chemical WO:[30] H_2O (pH 1, triflic acid), *c*(CAN) = 0.5 M, *c*(**2**) = 114–216 µM; Photocatalytic WO:[31] H_2O (pH 1, perchloric acid), *c*($[Ru(5\text{-}CF_3\text{-}bpy)_3]^{2+}$) = 0.2 mM, *c*($Na_2S_2O_8$) = 1 M, *c*(**2**) = 20 µM. [d] Chemical WO:[32] H_2O (pH 1, triflic acid), *c*(CAN) = 5 mM, *c*(**3**) = 50 nM; Photocatalytic WO:[33] 6:4 MeCN/H_2O (pH 6.8, phosphate buffer), *c*($[Ru(bpy)_3]^{2+}$) = 1 mM, *c*($Na_2S_2O_8$) = 45 mM, *c*(**3**) = 2 µM. [e] Chemical WO:[34] H_2O (pH 1, triflic acid), *c*(CAN) = 0.1 M, *c*(**4**) = 0.1–1 mM; Photocatalytic WO:[35] H_2O (pH 7, phosphate buffer), *c*($[Ru(4,4'\text{-}COOEt\text{-}bpy)_2bpy]^{2+}$) = 0.2 mM, *c*($Na_2S_2O_8$) = 20 mM, *c*(**4**) = 0.2 µM. [f] Photocatalytic WO:[36] H_2O (pH 7, phosphate buffer), *c*($[Ru(4,4'\text{-}COOEt\text{-}bpy)_2bpy]^{2+}$) = 0.2 mM, *c*($Na_2S_2O_8$) = 10 mM, *c*(**5**) = 1–16 µM. [g] Chemical WO:[37] H_2O (pH 1, triflic acid), *c*(CAN) = 0.33 M, *c*(**6**) = 0.2 µM; Photocatalytic WO:[38] H_2O (pH 7, phosphate buffer), *c*($[Ru(bpy)_3]^{2+}$) = 1 mM, *c*($Na_2S_2O_8$) = 30 mM, *c*(**6**) = 26 µM. [h] Chemical WO:[39] H_2O (pH 1, triflic acid), *c*(CAN) = 0.1 M, *c*(**7**) = 1 mM. [i] Chemical WO:[40] H_2O (pH 1, perchloric acid), *c*(CAN) = 0.33 M, *c*(**8**) = 11 mM. n.d.: not determined.

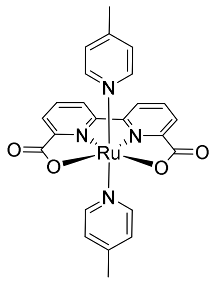
1

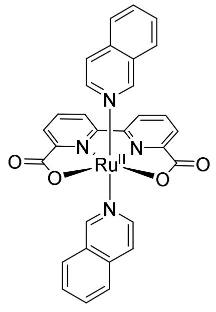
2

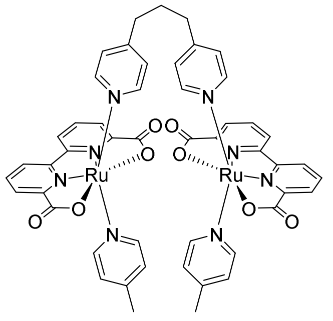
3

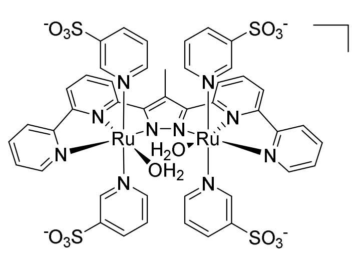
4

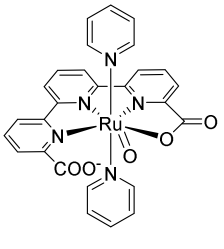
5

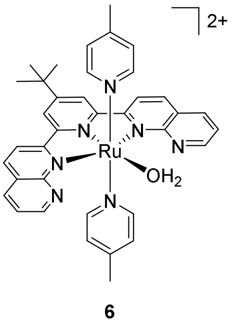
6

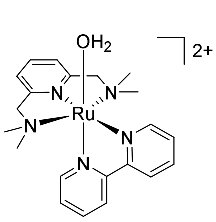
7

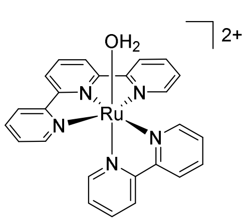
8

8. Theoretical studies

Table S5 Energy of optimized structures of **MC3** and methylated derivatives.[a]

Conformation	Optimized structure energy [kJ mol^{-1}]		
	***m*-Me-MC3**	**MC3**	***p*-Me-MC3**
**A**	0	0	0
**B**	-22.5	-18.5	0.8
**C**	---	---	136.5
**D**	55.1	63.2	53.4

[a] Optimized structures of **MC3** and methylated macrocycles in conformations **A-D** (as depicted in Fig. 8a in main article) at Ru^{II}_3 oxidation state were determined on the basis of PM6/COSMO model.

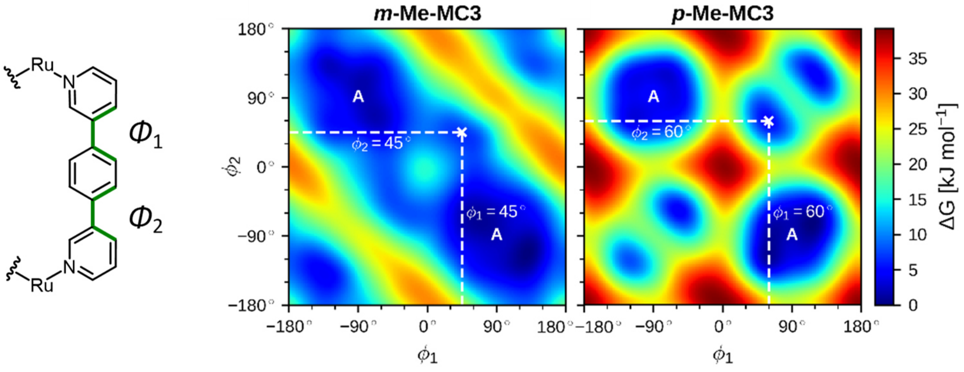

Fig. S52 Free energy surfaces of ***m*-Me-MC3** (left) and ***p*-Me-MC3** (right) obtained from metadynamic studies on the distortion of one of the bridging ligands starting from conformation **A** in aqueous solution. $\Phi_{1,2}$ are the torsion angles within the bridging ligand which were used as collective variables. The white cross indicates exemplary **A₂B** configuration of each macrocycle.

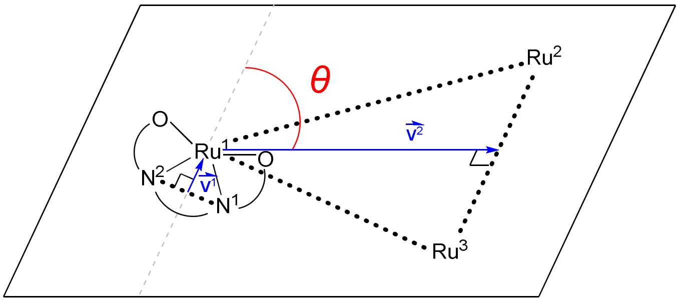

$$\vec{v^1} = \vec{Ru^1} + \frac{(\vec{N^1} + \vec{N^2})}{2}$$

$$\vec{v^2} = -\vec{Ru^1} + \frac{(\vec{Ru^1} + \vec{Ru^2})}{2}$$

$$\theta = \cos^{-1}\left(\frac{\vec{v^1} \cdot \vec{v^2}}{|\vec{v^1}||\vec{v^2}|}\right)$$

Fig. S53 Definition of the angle θ used as collective variable to assess the rotation of the Ru(bda) unit within the macrocyclic structure.

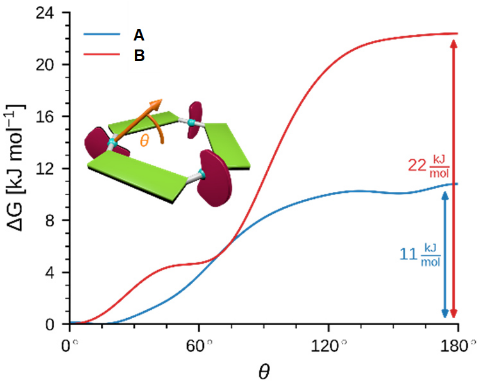

Fig. S54 Free energy profiles obtained from metadynamics studies of ***p*-Me-MC3** using the angle θ for the rotation of the Ru(bda) unit as collective variable. Quadratic wall potentials have been applied to the angles $\Phi_{1,2}$ in the bridging ligands in order to limit the sampling space corresponding to conformations **A** (blue curve) and **B** (red curve), respectively.

Table S6 Energy of optimized structures of **MC3** at Ru^{II}_3 and Ru^{IV}_3 oxidation states.[a]

Conformation	Optimized structure energy [kJ mol^{-1}]	
	**Ru^{II}_3**	**Ru^{IV}_3**
**A**	0	0
**B**	-19.7	-35.0
**C**	---	120.4
**D**	23.2	115.8

[a] Optimized structures of **MC3** in conformations **A-D** (as depicted in Fig. 8a in main article) at Ru^{II}_3 and Ru^{IV}_3 oxidation states determined by PBE/COSMO model.

9. PXRD

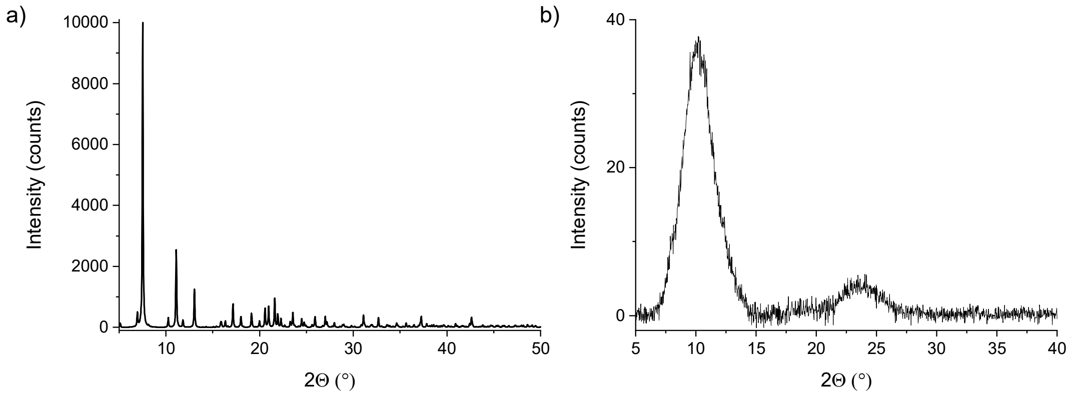

Fig. S55 a) Calculated powder diffraction pattern for single crystals of ***m*-F-MC3**. b) Experimental diffraction pattern obtained for vacuum-dried crystals of ***m*-F-MC3**.

To determine whether the solid-state structure of ***m*-F-MC3** remained porous after removal the solvent molecules, crystals of this macrocycle were dried under high vacuum prior to analysis by PXRD. As shown in Fig. S55, the crystal packing of ***m*-F-MC3** clearly depended on the presence of solvent molecules, since upon their removal a collapse of the ordered structure was observed by comparison with the calculated pattern from the X-ray structure of this compound.

10. NMR spectra

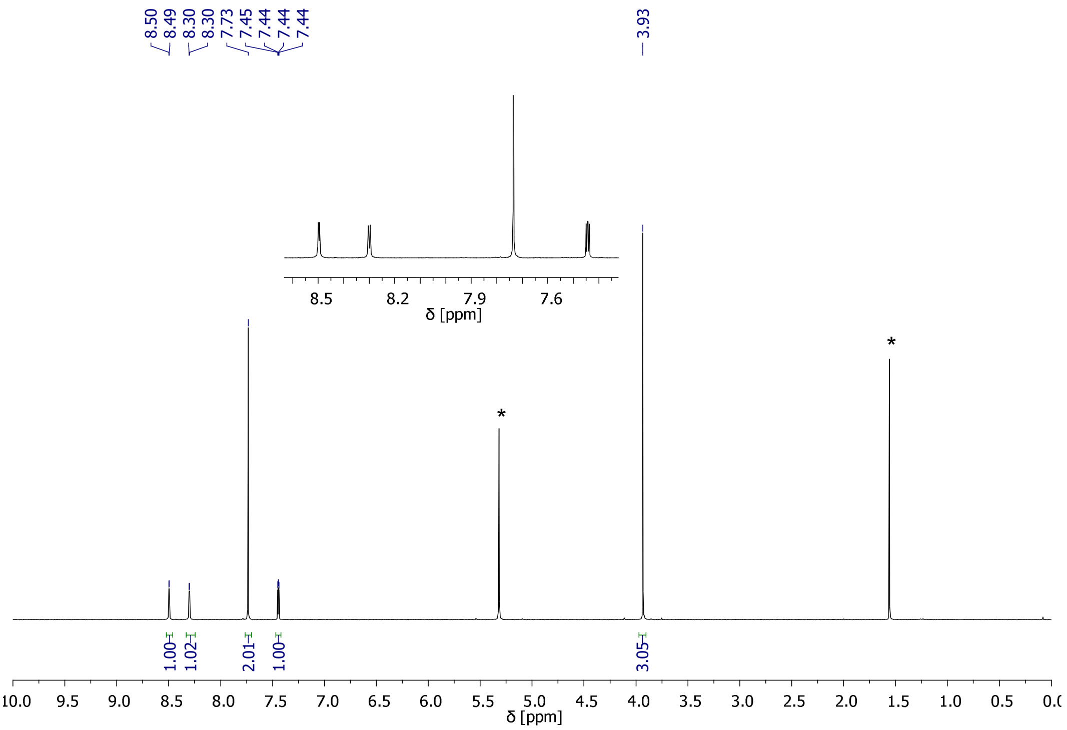

Fig. S56 ^{1}H NMR spectrum (400 MHz, CD_2Cl_2) of ***m*-MeO-bpb** (* residual solvent).

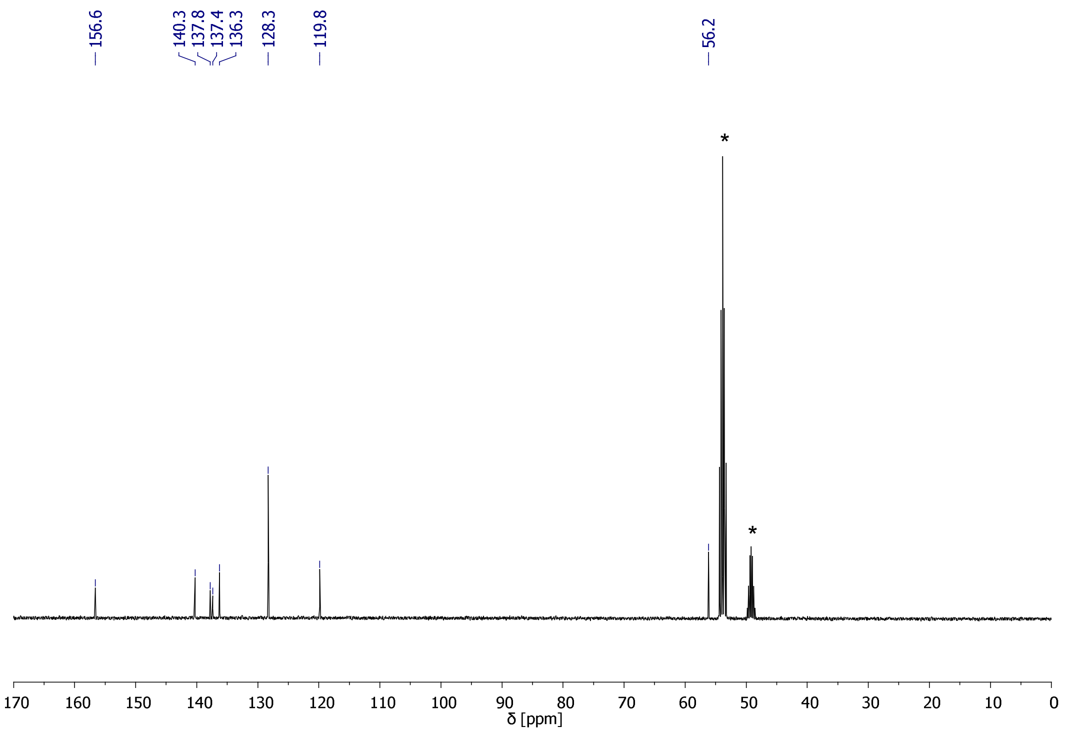

Fig. S57 ^{13}C NMR spectrum (100 MHz, CD_2Cl_2/CD_3OD) of ***m*-MeO-bpb** (* residual solvent).

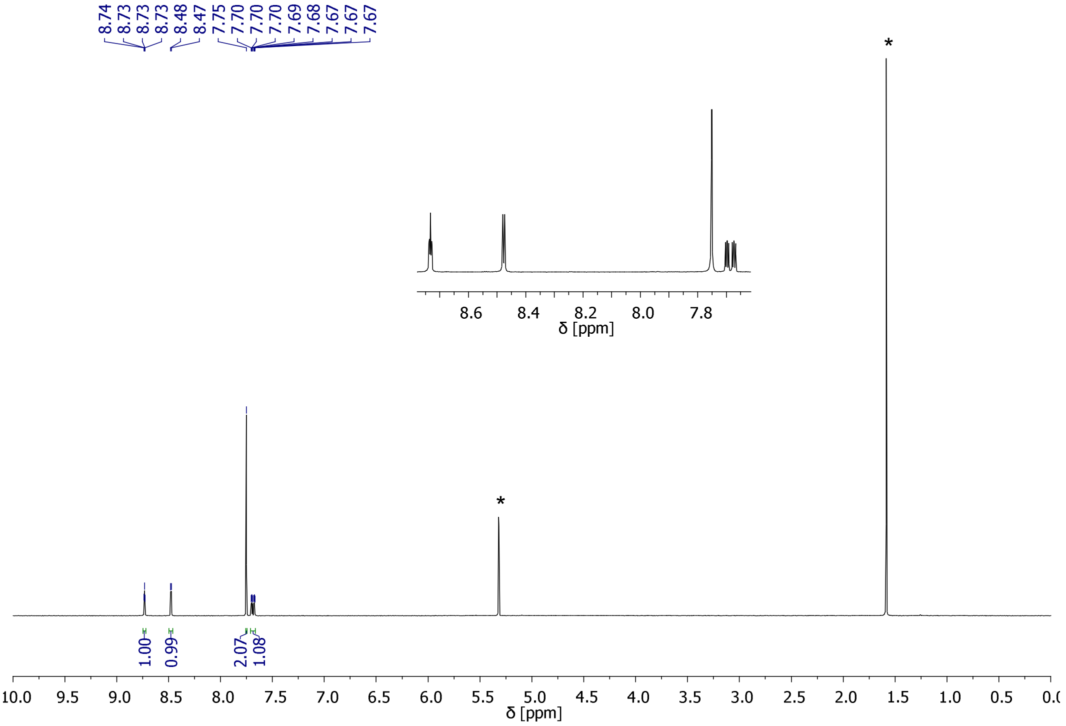

Fig. S58 ^{1}H NMR spectrum (400 MHz, CD_2Cl_2) of ***m*-F-bpb** (* residual solvent).

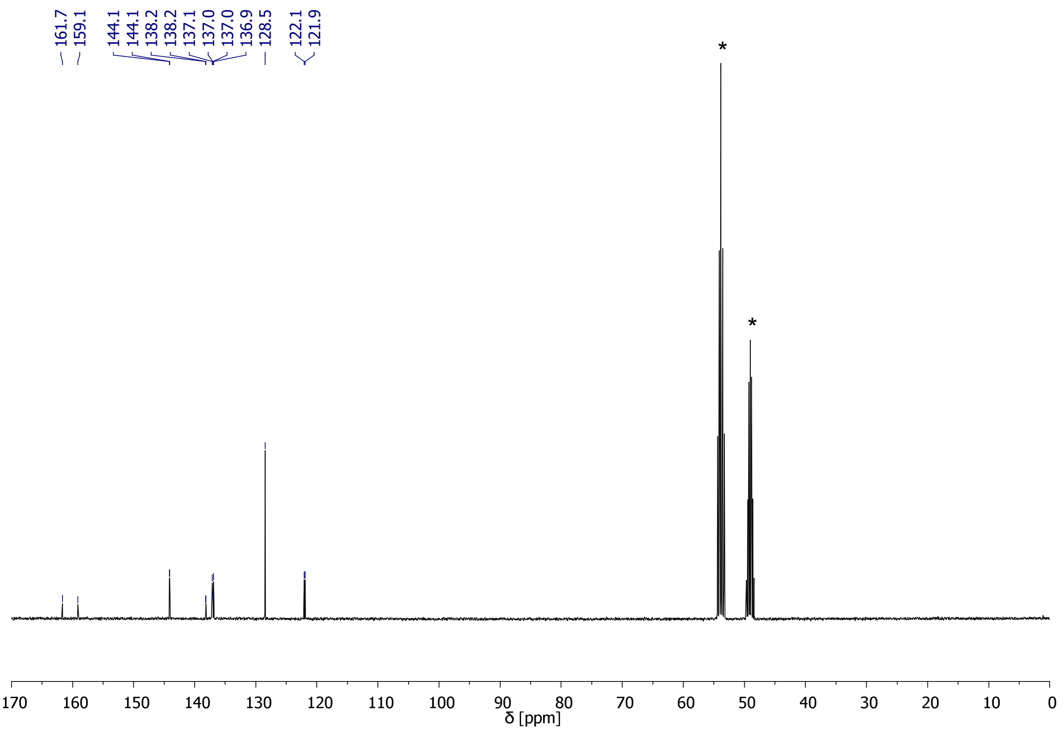

Fig. S59 ^{13}C NMR spectrum (100 MHz, CD_2Cl_2/CD_3OD) of ***m*-F-bpb** (* residual solvent).

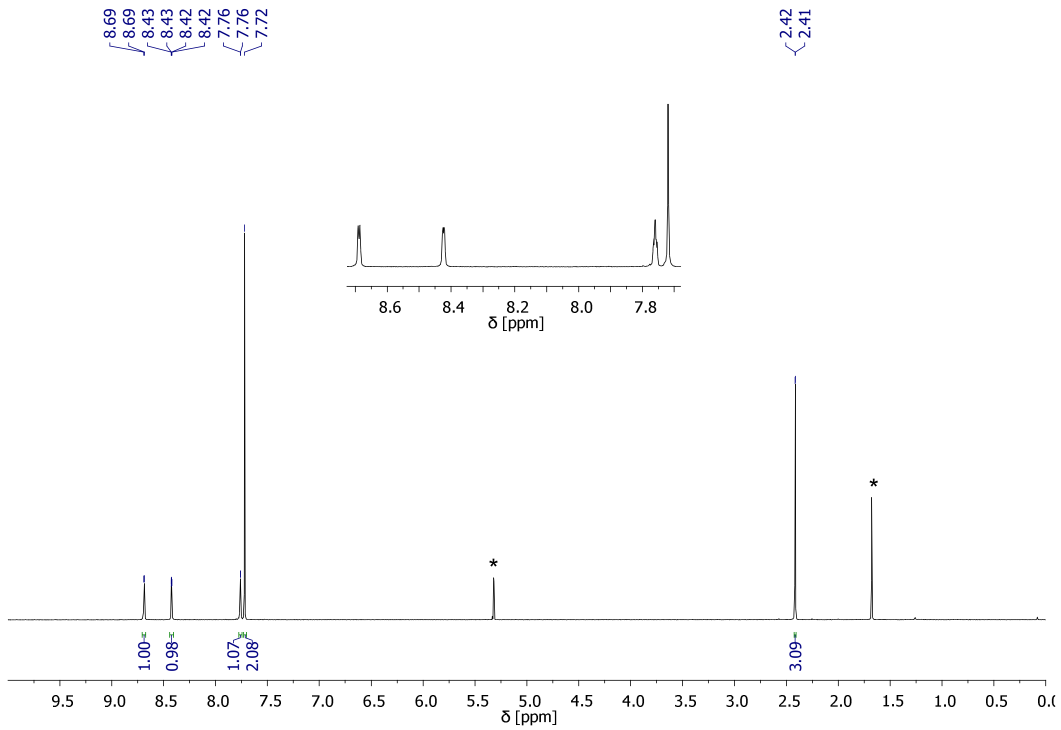

Fig. S60 ^{1}H NMR spectrum (400 MHz, CD_2Cl_2) of ***m*-Me-bpb** (* residual solvent).

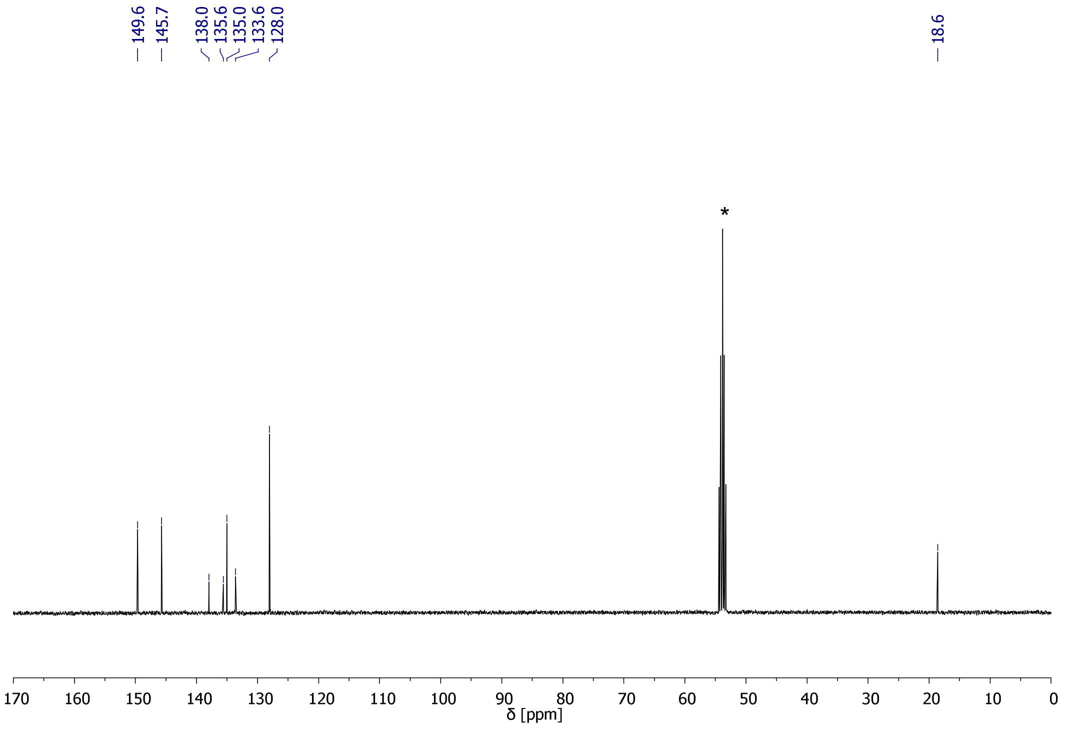

Fig. S61 ^{13}C NMR spectrum (100 MHz, CD_2Cl_2) of ***m*-Me-bpb** (* residual solvent).

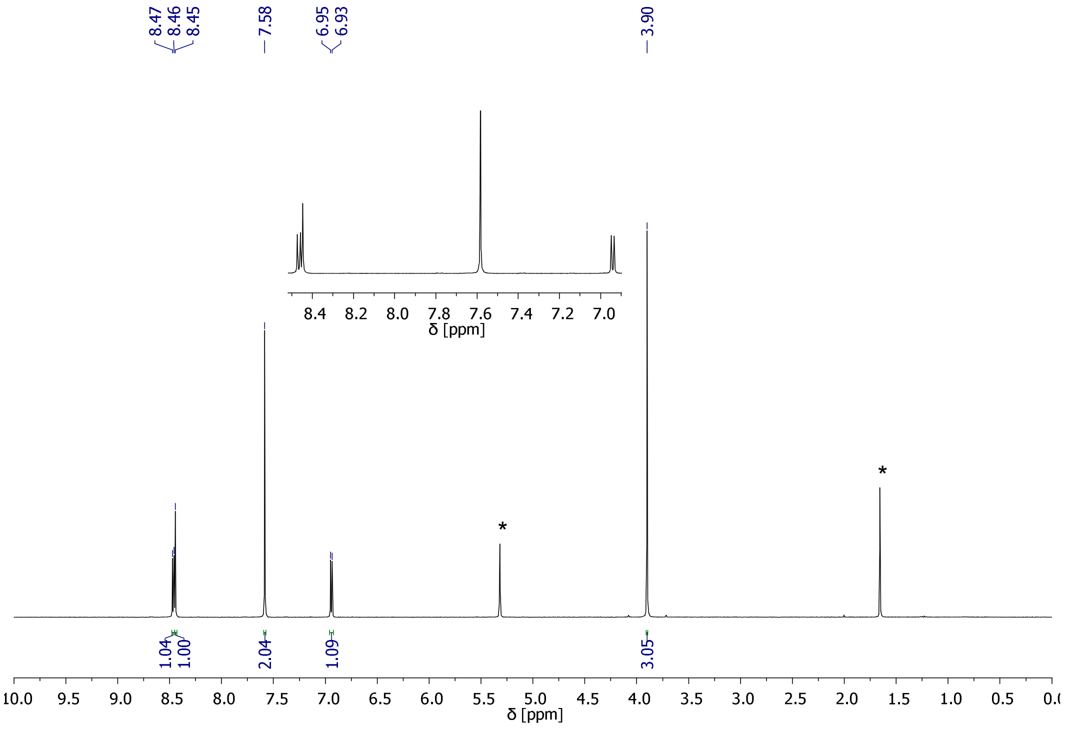

Fig. S62 ^{1}H NMR spectrum (400 MHz, CD_2Cl_2) of ***p*-MeO-bpb** (* residual solvent).

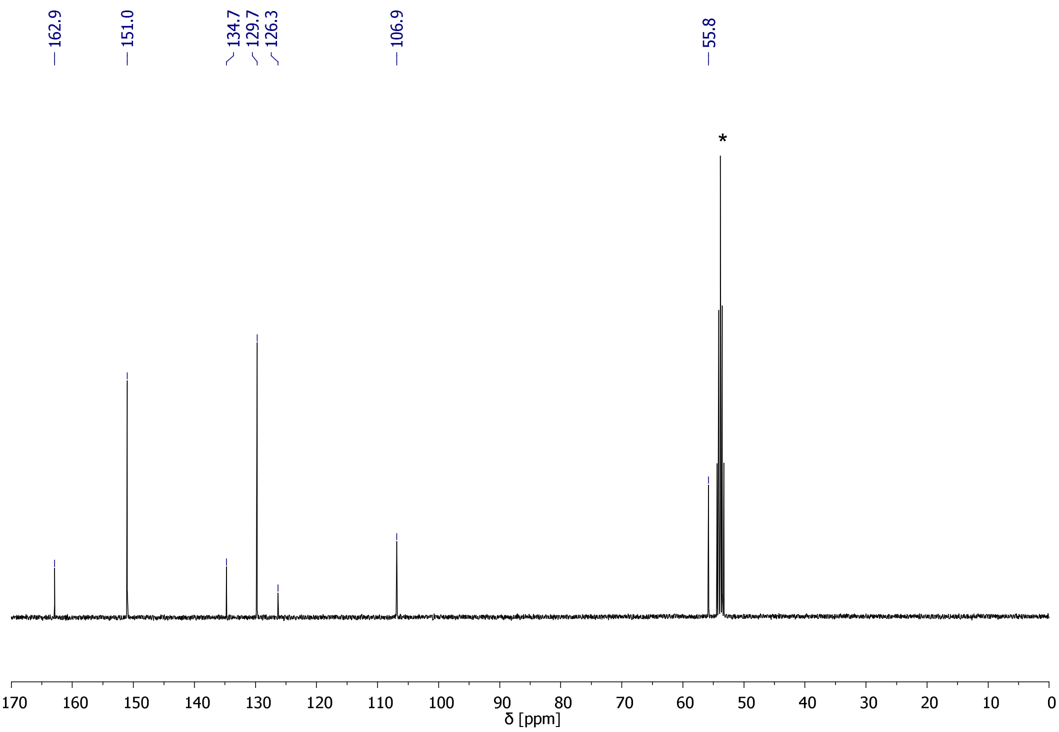

Fig. S63 ^{13}C NMR spectrum (100 MHz, CD_2Cl_2) of ***p*-MeO-bpb** (* residual solvent).

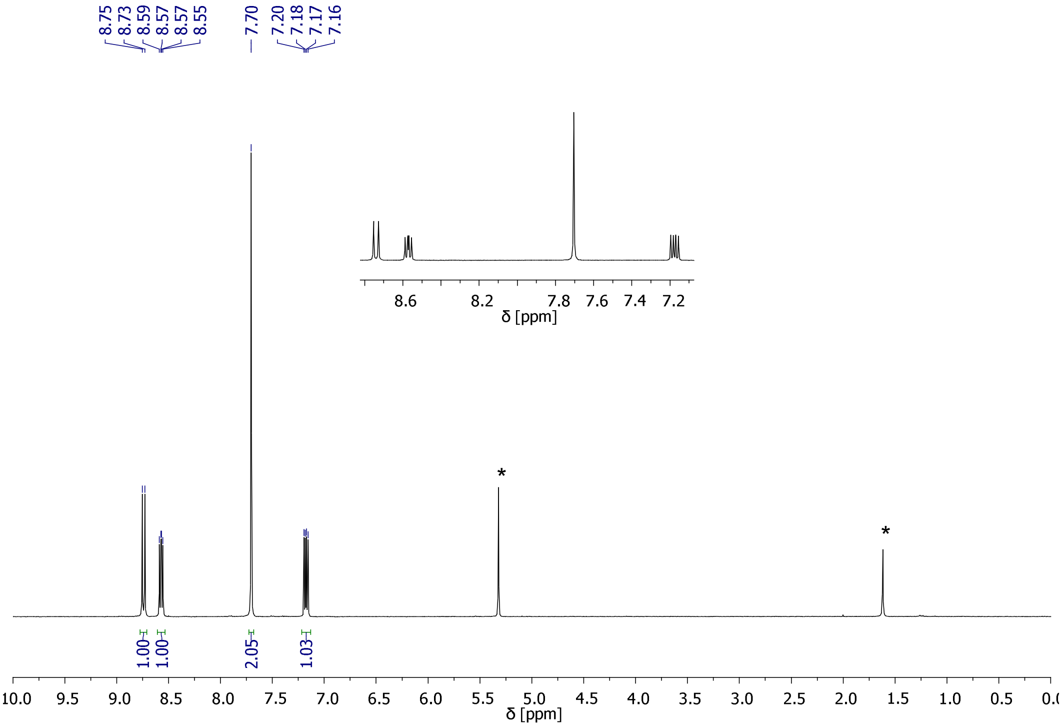

Fig. S64 ^{1}H NMR spectrum (400 MHz, CD_2Cl_2) of ***p*-F-bpb** (* residual solvent).

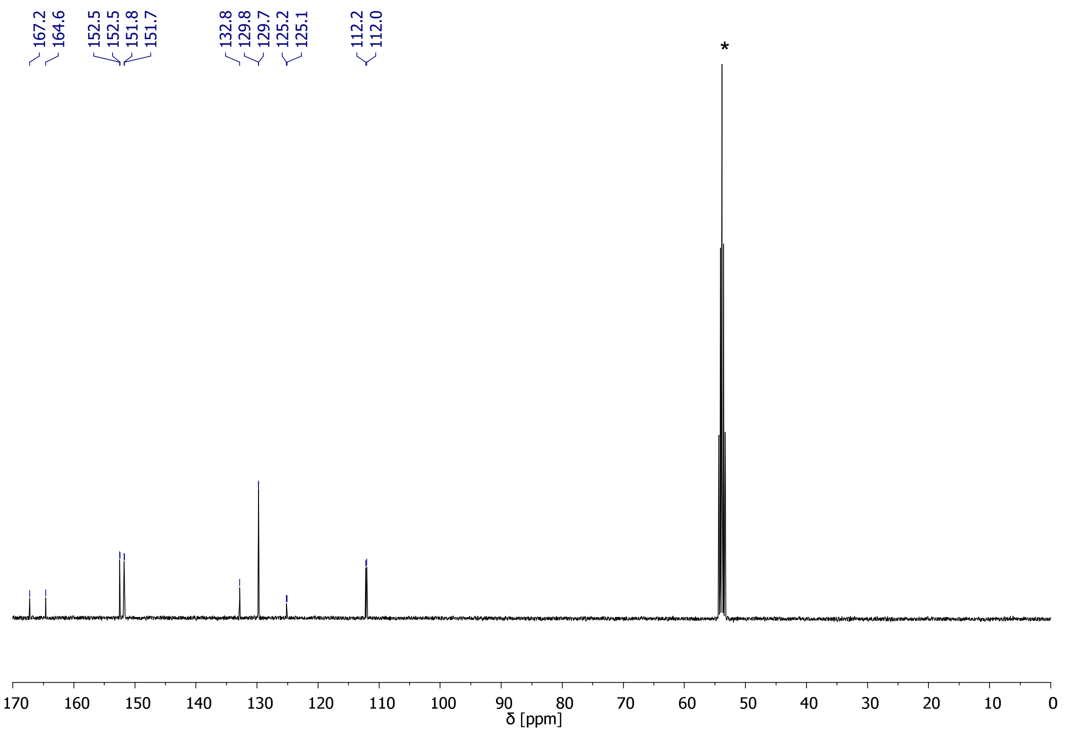

Fig. S65 ^{13}C NMR spectrum (100 MHz, CD_2Cl_2) of ***p*-F-bpb** (* residual solvent).

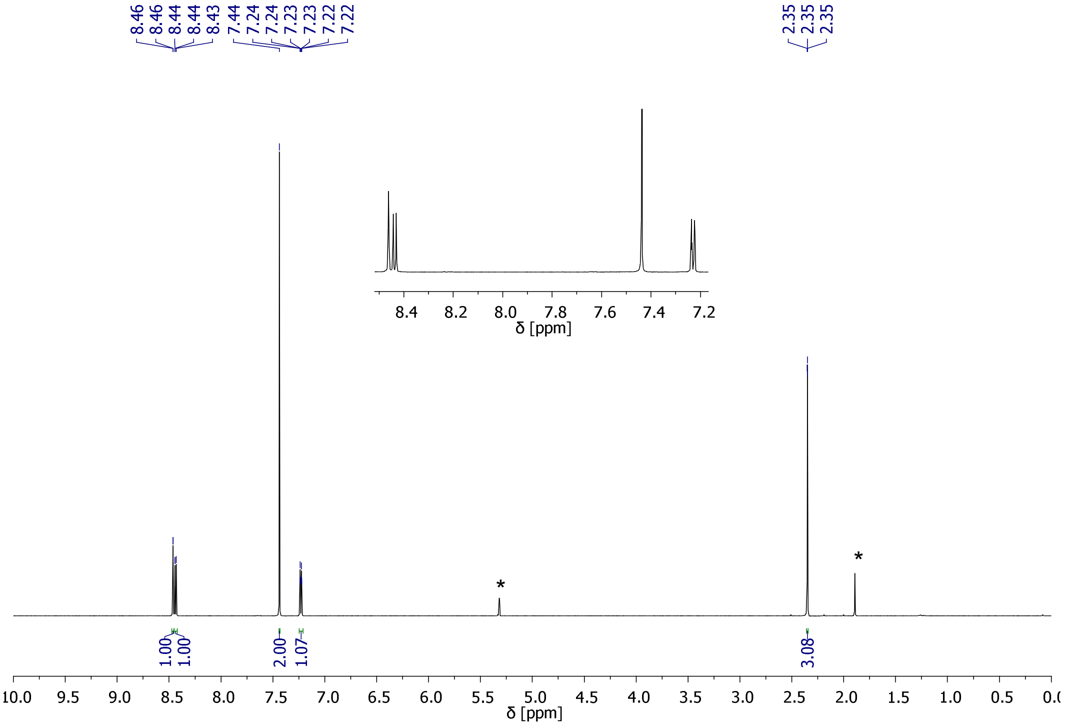

Fig. S66 ^{1}H NMR spectrum (400 MHz, CD_2Cl_2) of ***p*-Me-bpb** (* residual solvent).

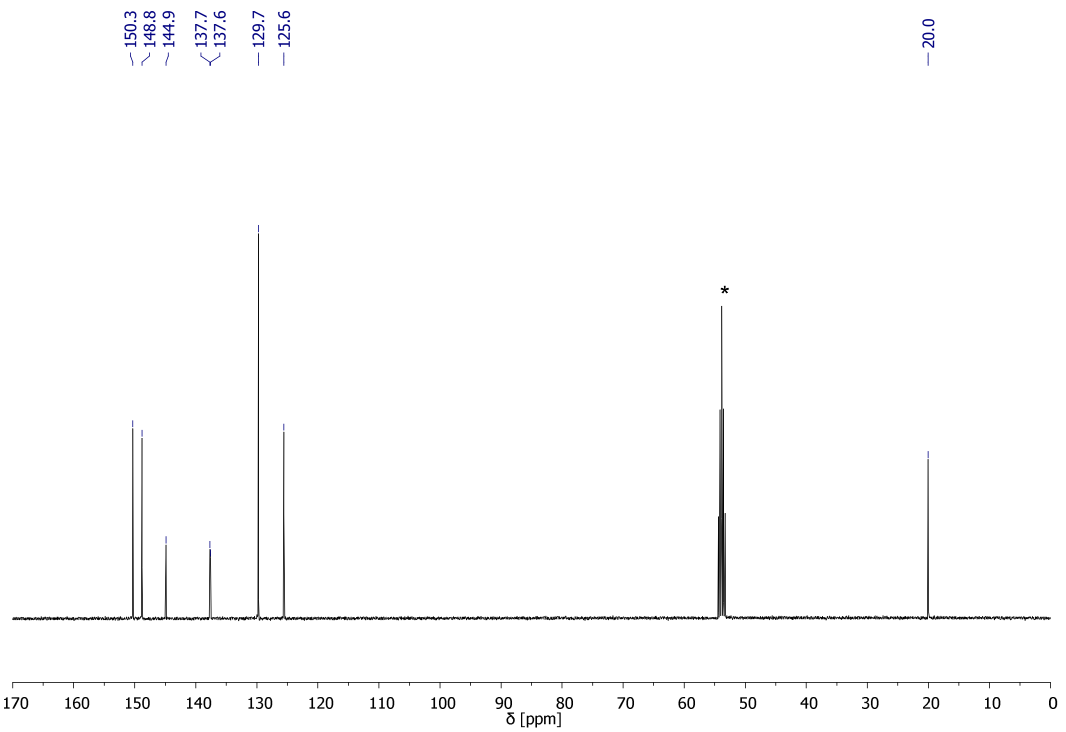

Fig. S67 ^{13}C NMR spectrum (100 MHz, CD_2Cl_2) of ***p*-Me-bpb** (* residual solvent).

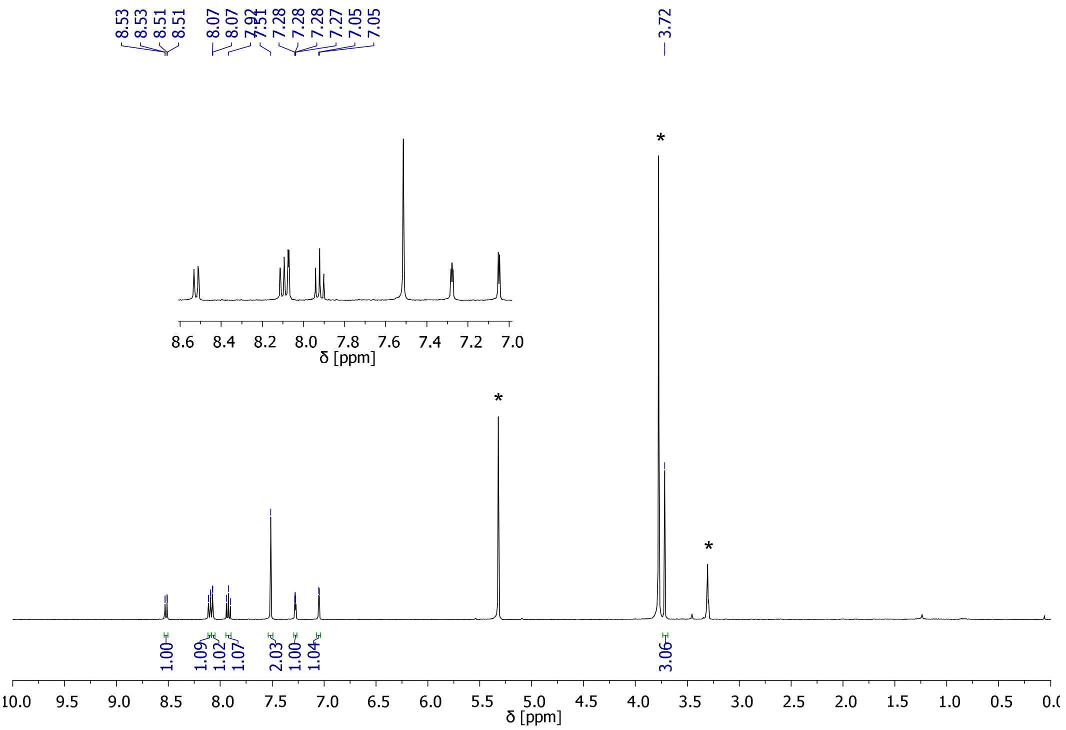

Fig. S68 ^{1}H NMR spectrum (400 MHz, CD_2Cl_2/CD_3OD) of ***m*-MeO-MC3** (* residual solvent).

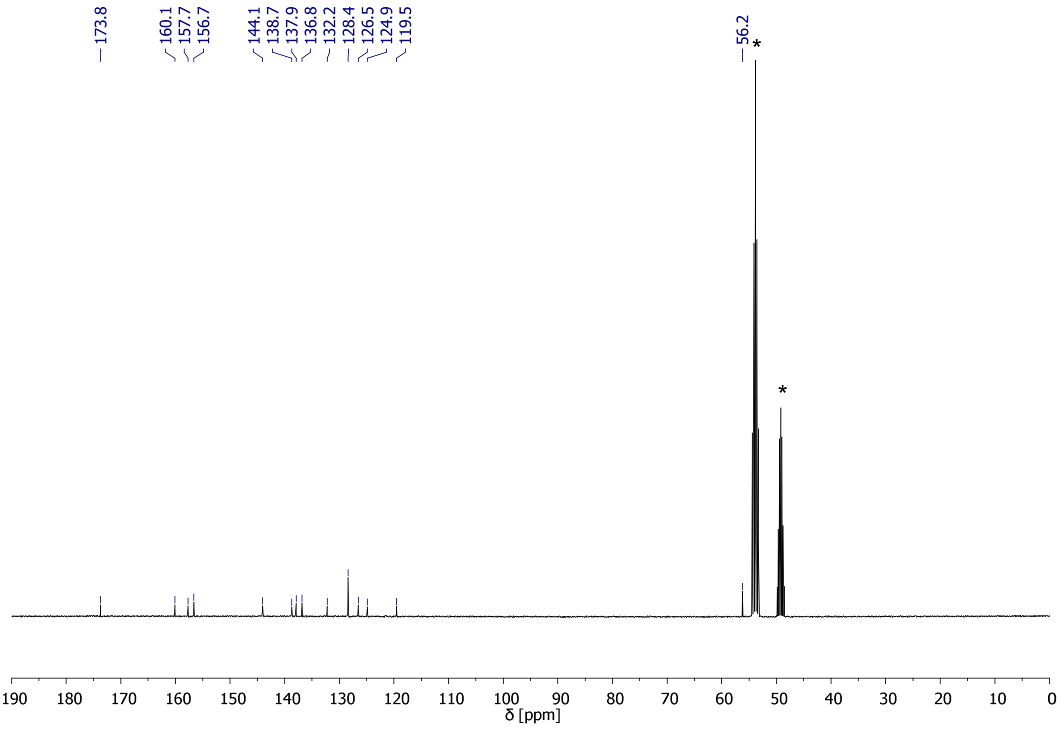

Fig. S69 ^{13}C NMR spectrum (100 MHz, CD_2Cl_2/CD_3OD) of ***m*-MeO-MC3** (* residual solvent).

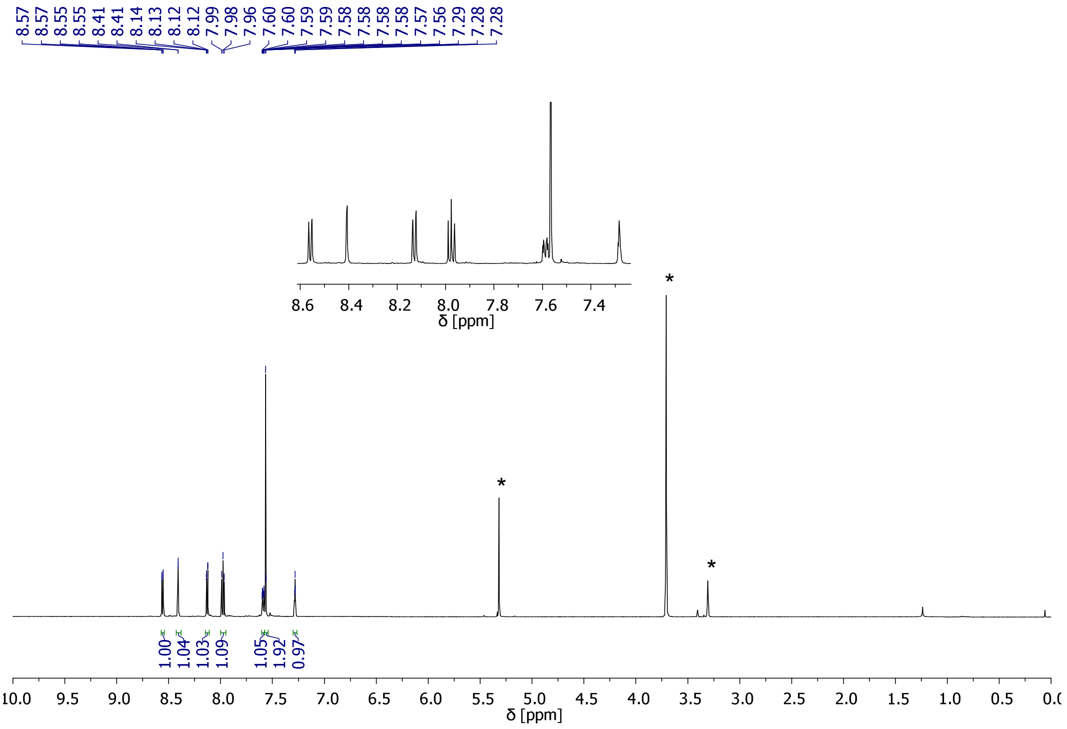

Fig. S70 ^{1}H NMR spectrum (600 MHz, CD_2Cl_2/CD_3OD) of ***m*-F-MC3** (* residual solvent).

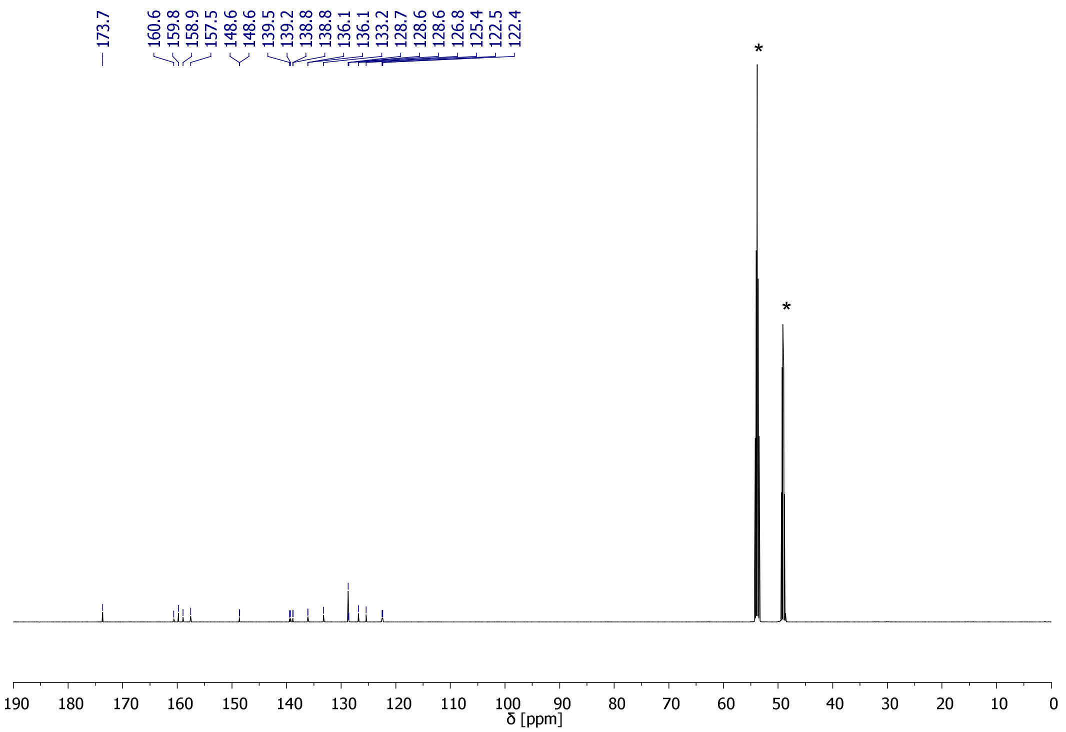

Fig. S71 ^{13}C NMR spectrum (151 MHz, CD_2Cl_2/CD_3OD) of ***m*-F-MC3** (* residual solvent).

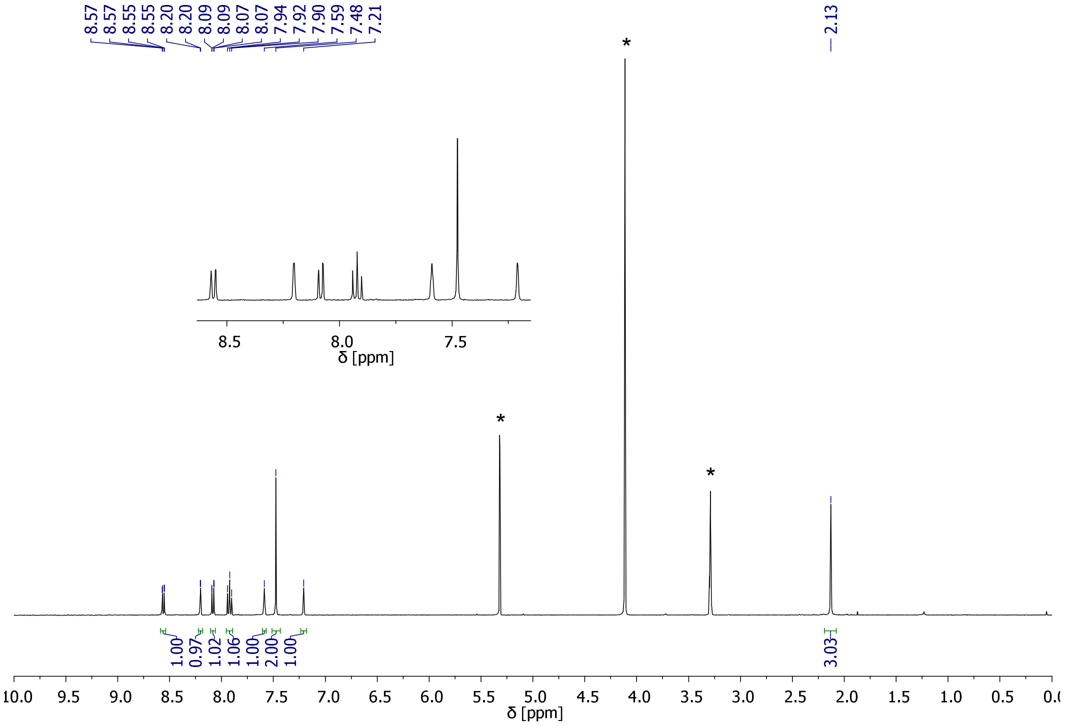

Fig. S72 ^{1}H NMR spectrum (400 MHz, CD_2Cl_2/CD_3OD) of ***m*-Me-MC3** (* residual solvent).

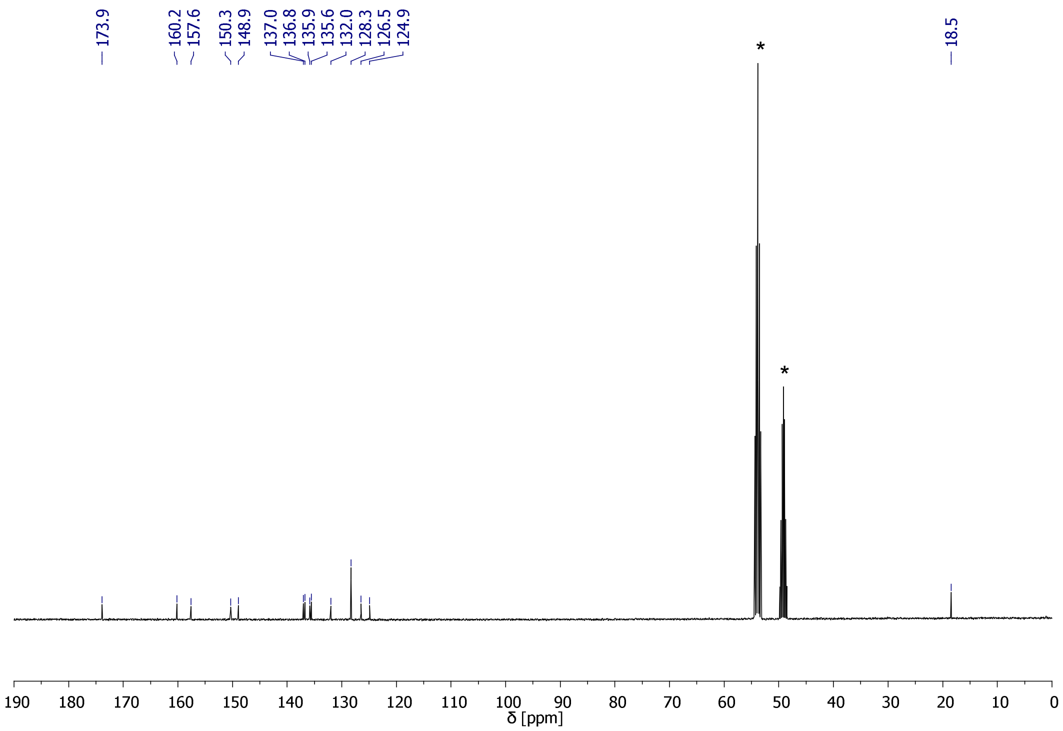

Fig. S73 ^{13}C NMR spectrum (100 MHz, CD_2Cl_2/CD_3OD) of ***m*-Me-MC3** (* residual solvent).

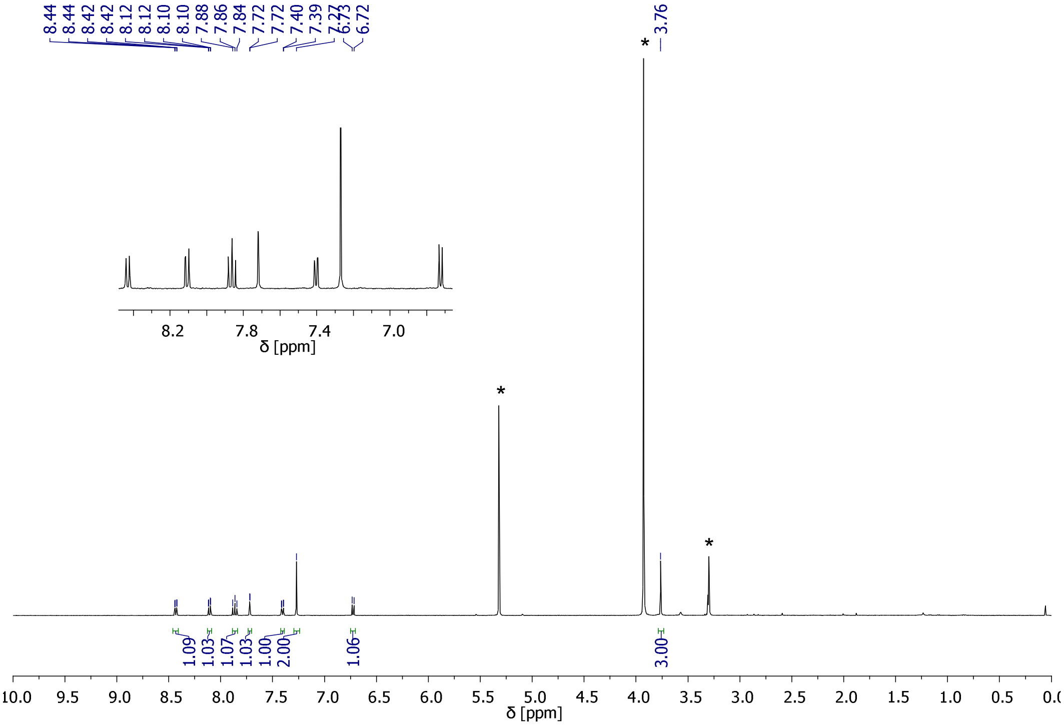

Fig. S74 ^{1}H NMR spectrum (400 MHz, CD_2Cl_2/CD_3OD) of ***p*-MeO-MC3** (* residual solvent).

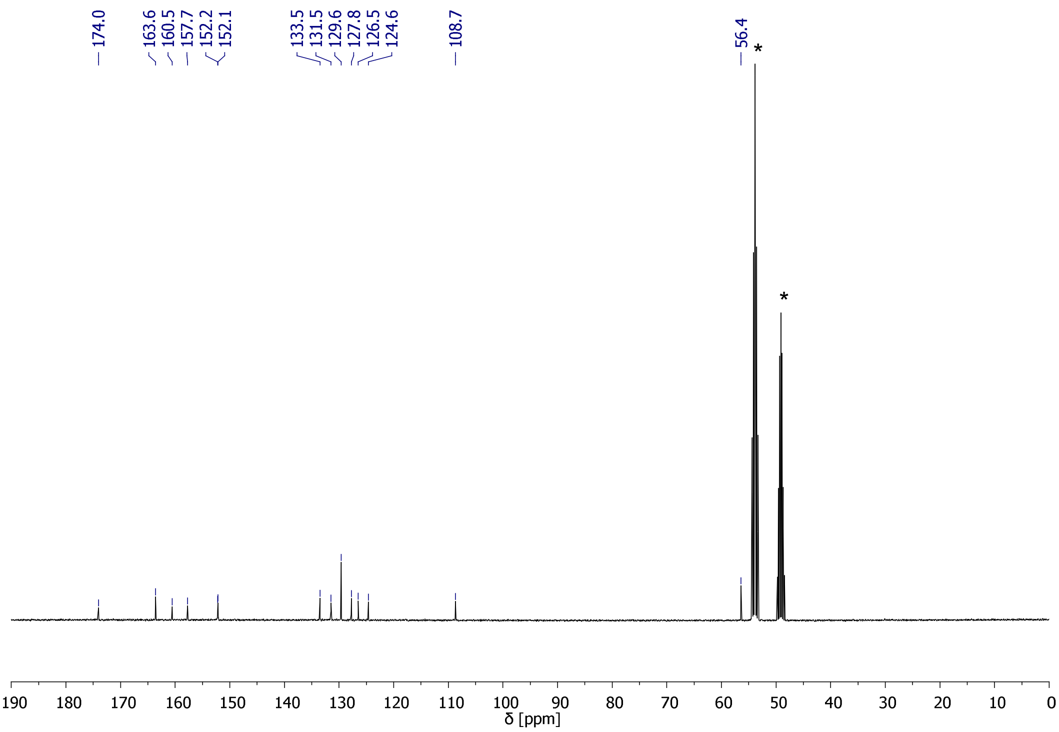

Fig. S75 ^{13}C NMR spectrum (100 MHz, CD_2Cl_2/CD_3OD) of ***p*-MeO-MC3** (* residual solvent).

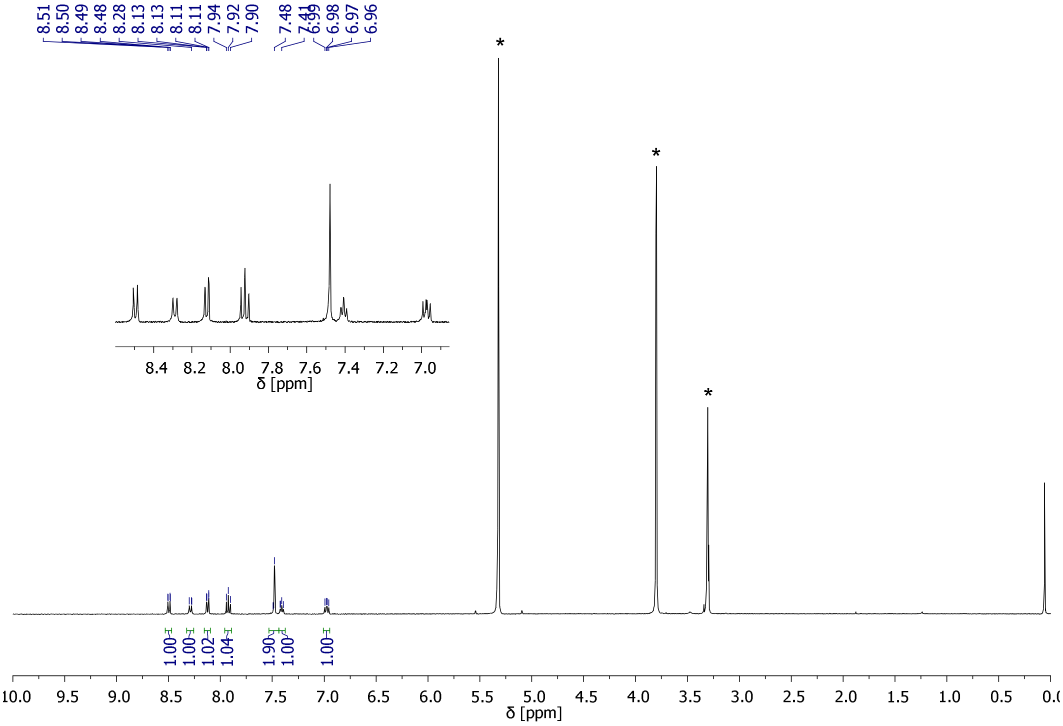

Fig. S76 ^{1}H NMR spectrum (600 MHz, CD_2Cl_2/CD_3OD) of ***p*-F-MC3** (* residual solvent).

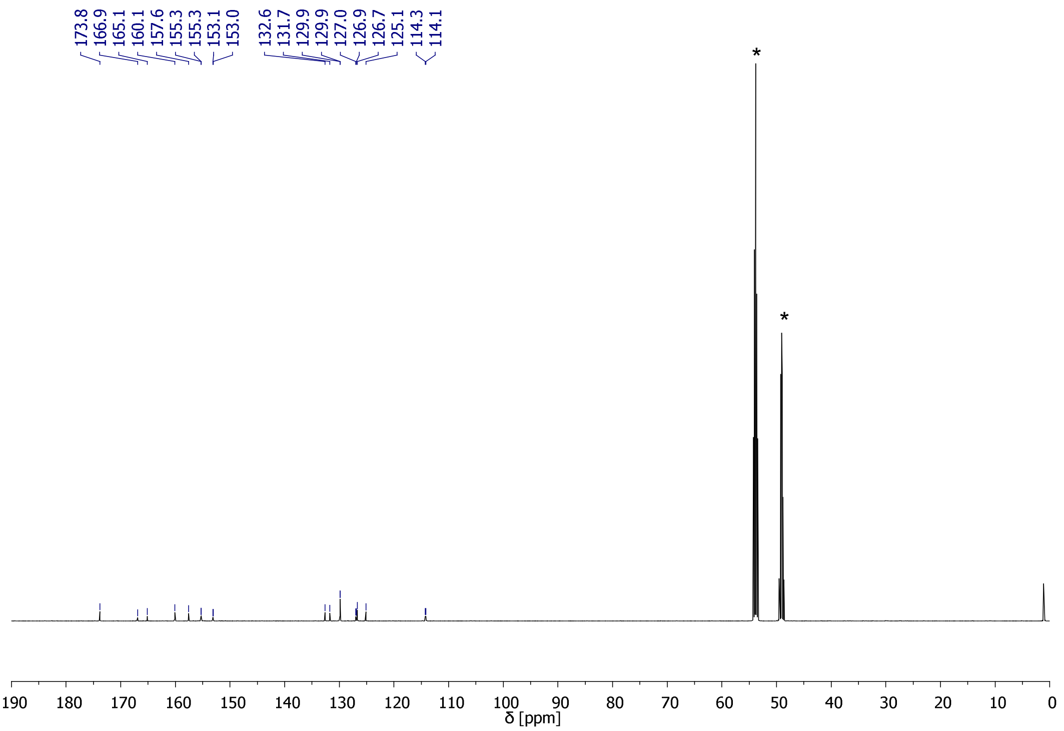

Fig. S77 ^{13}C NMR spectrum (151 MHz, CD_2Cl_2/CD_3OD) of ***p*-F-MC3** (* residual solvent).

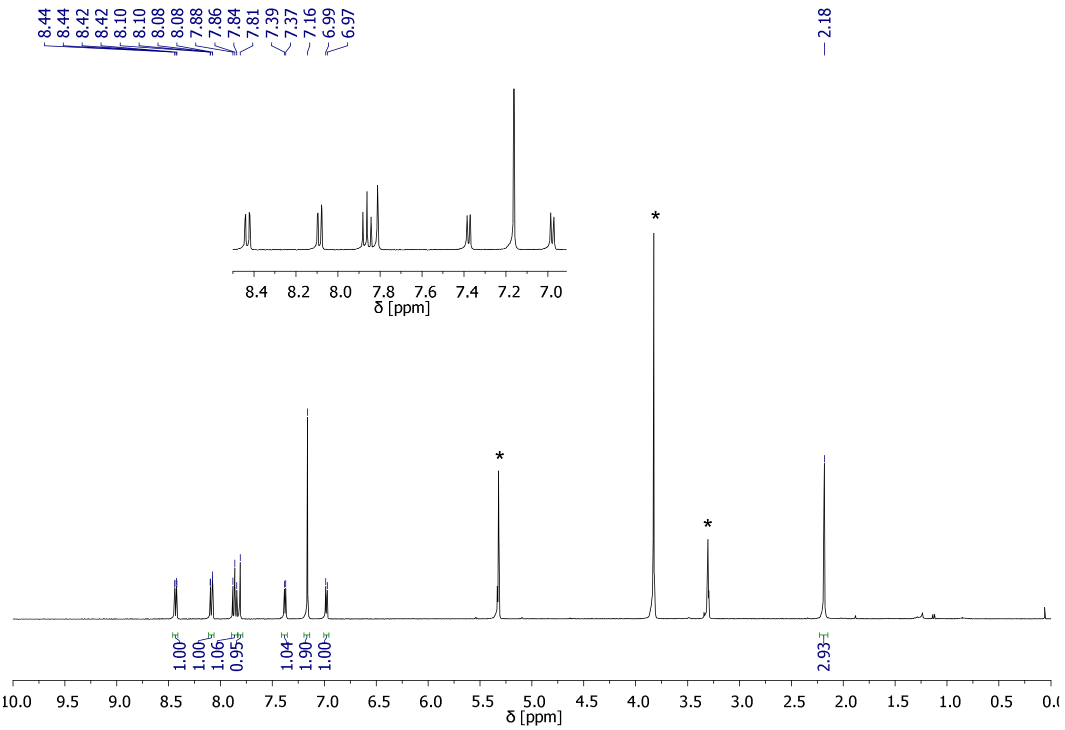

Fig. S78 ^{1}H NMR spectrum (400 MHz, CD_2Cl_2/CD_3OD) of ***p*-Me-MC3** (* residual solvent).

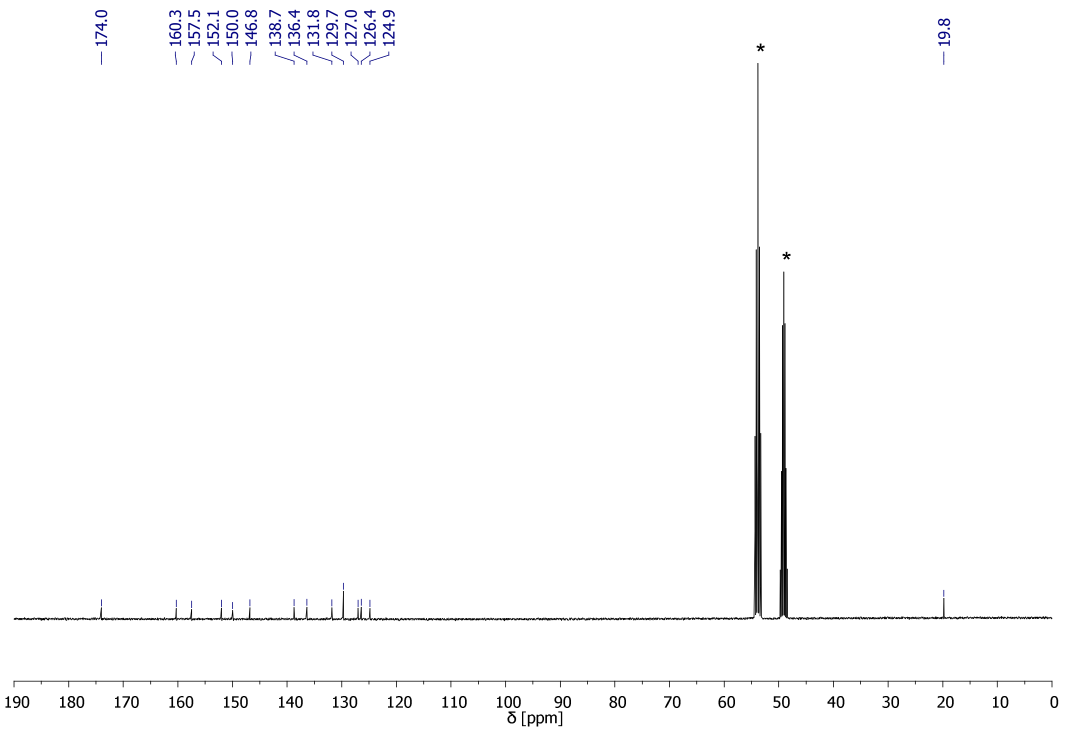

Fig. S79 ^{13}C NMR spectrum (100 MHz, CD_2Cl_2/CD_3OD) of ***p*-Me-MC3** (* residual solvent).

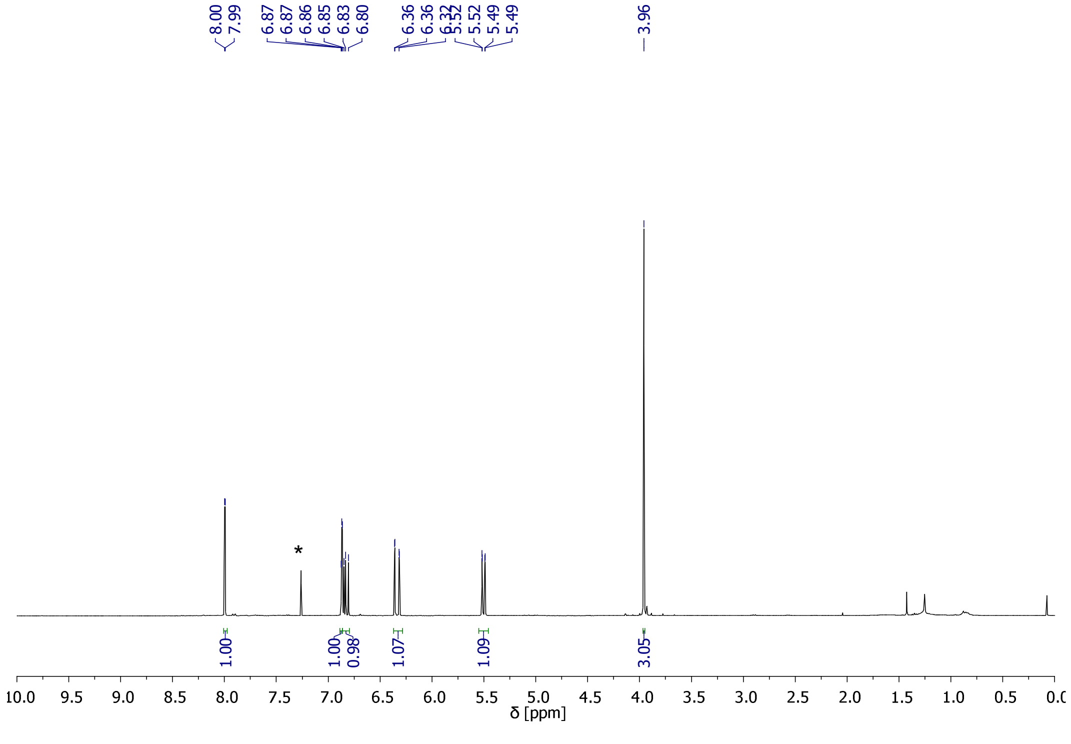

Fig. S80 ^{1}H NMR spectrum (400 MHz, $CDCl_3$) of compound **6** (* residual solvent).

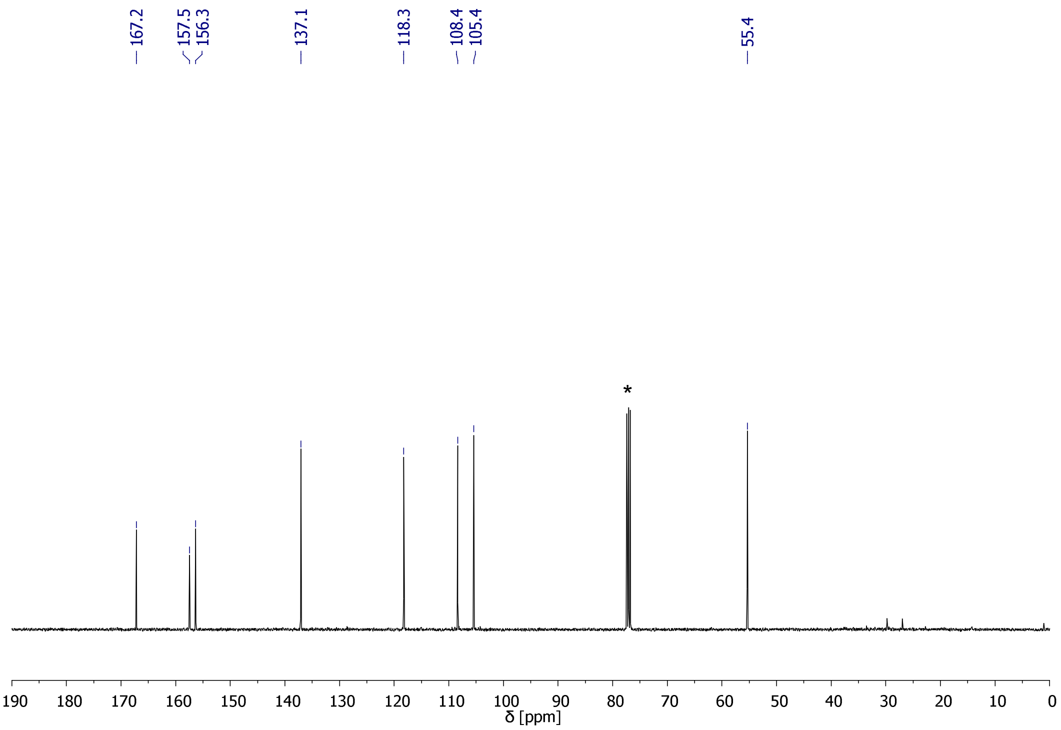

Fig. S81 ^{13}C NMR spectrum (100 MHz, $CDCl_3$) of compound **6** (* residual solvent).

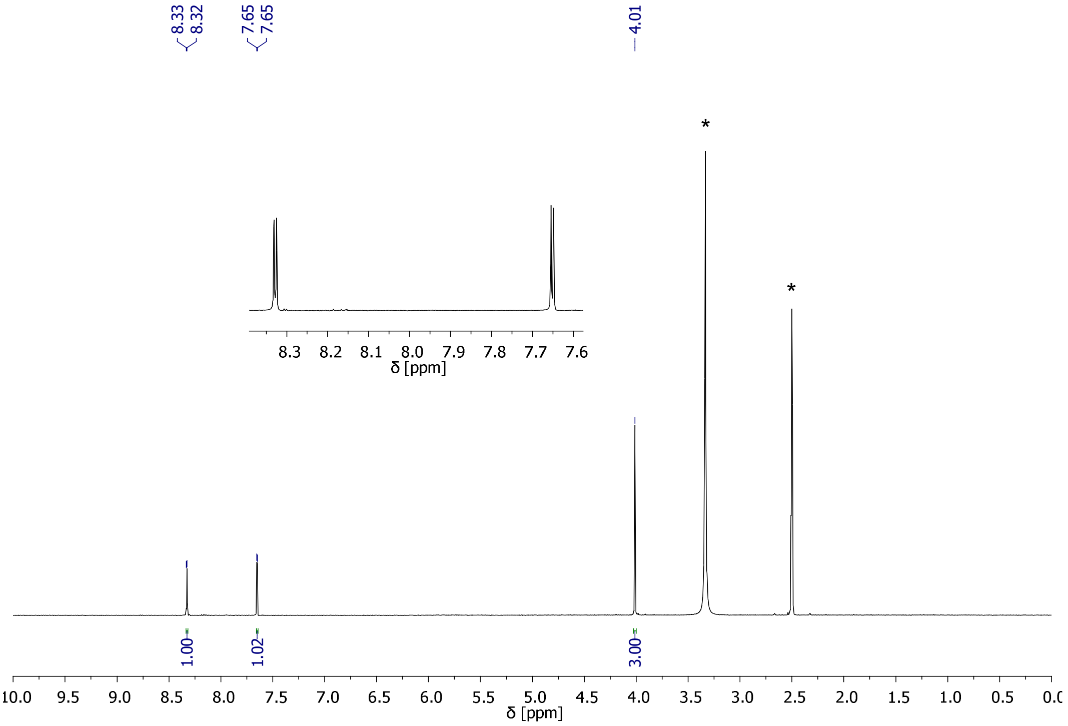

Fig. S82 ^{1}H NMR spectrum (400 MHz, DMSO-d_6) of compound **7** (* residual solvent).

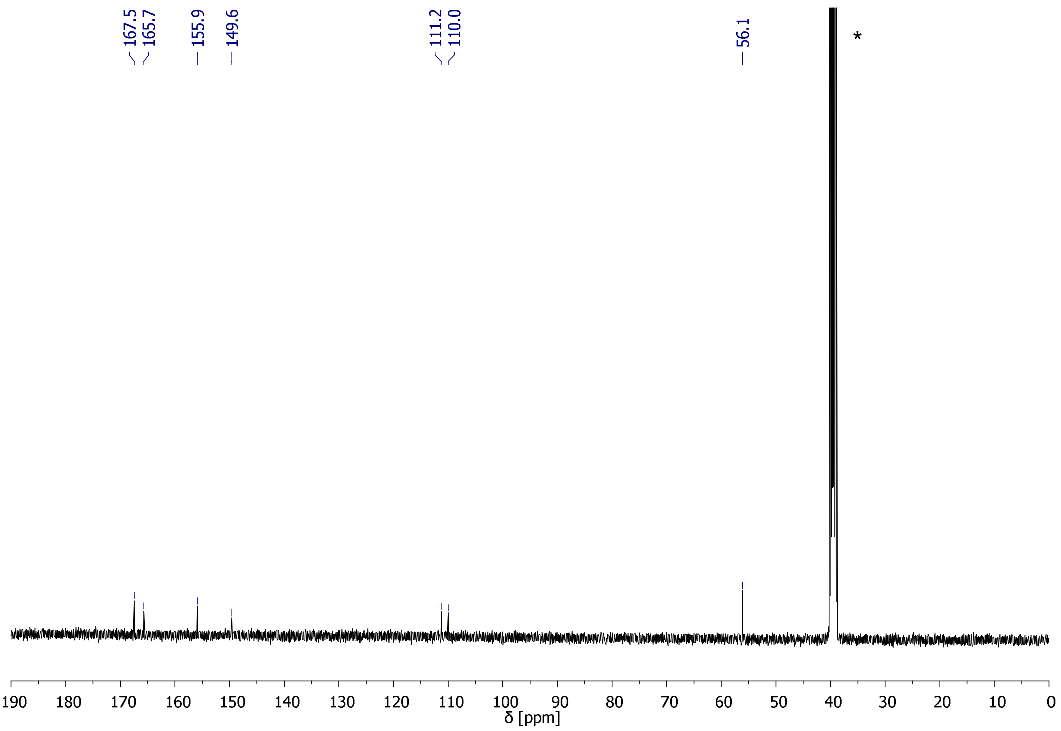

Fig. S83 ^{13}C NMR spectrum (100 MHz, DMSO-d_6) of compound **7** (* residual solvent).

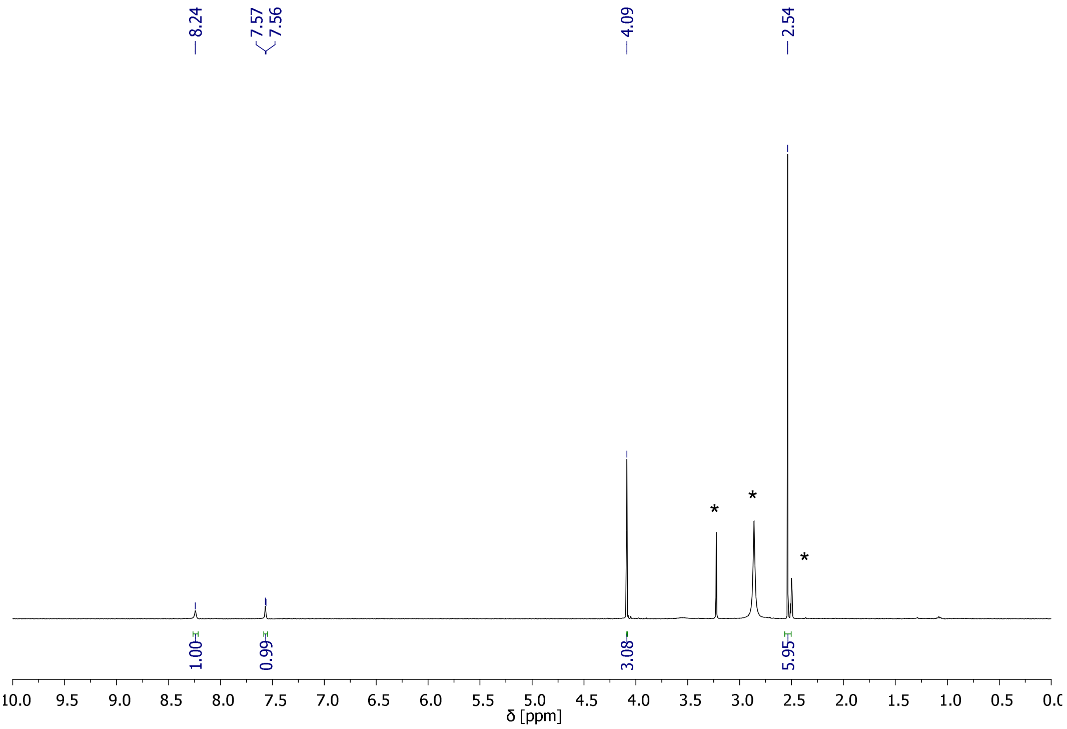

Fig. S84 ^{1}H NMR spectrum (400 MHz, 399 K, DMSO-d_6) of compound **8** (* residual solvent).

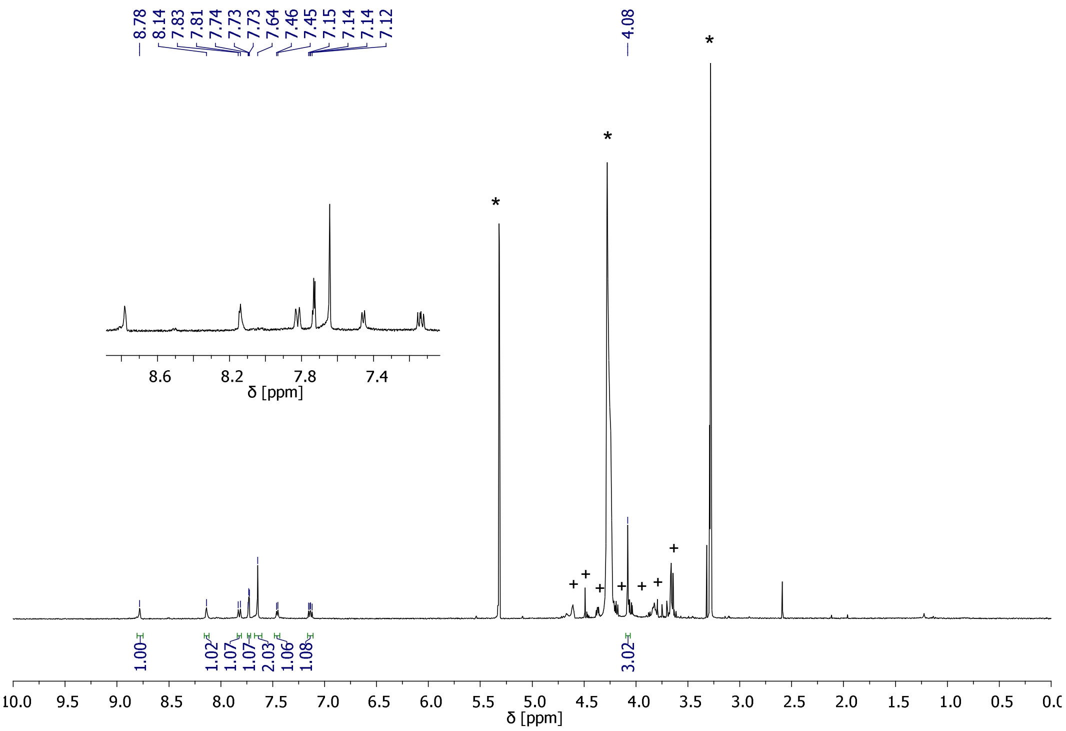

Fig. S85 ^{1}H NMR spectrum (400 MHz, CD_2Cl_2/CD_3OD) of **MeO-bda-MC3** (* residual solvent, $^{+}$ ascorbic acid).

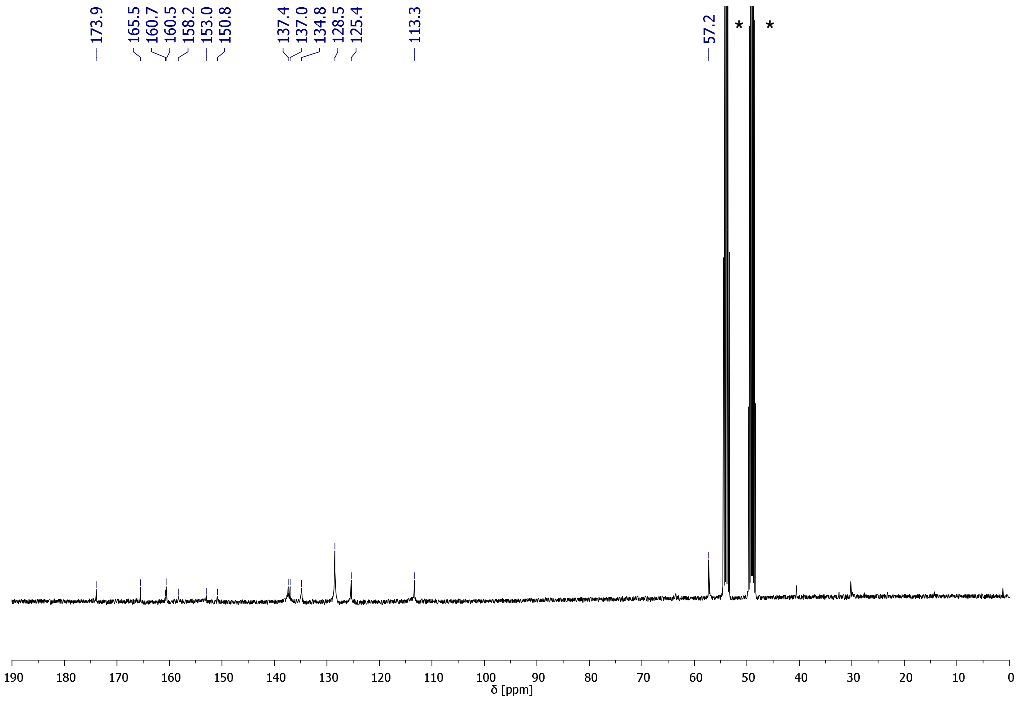

Fig. S86 ^{13}C NMR spectrum (100 MHz, CD_2Cl_2/CD_3OD) of **MeO-bda-MC3** (* residual solvent).

11. HR mass spectra

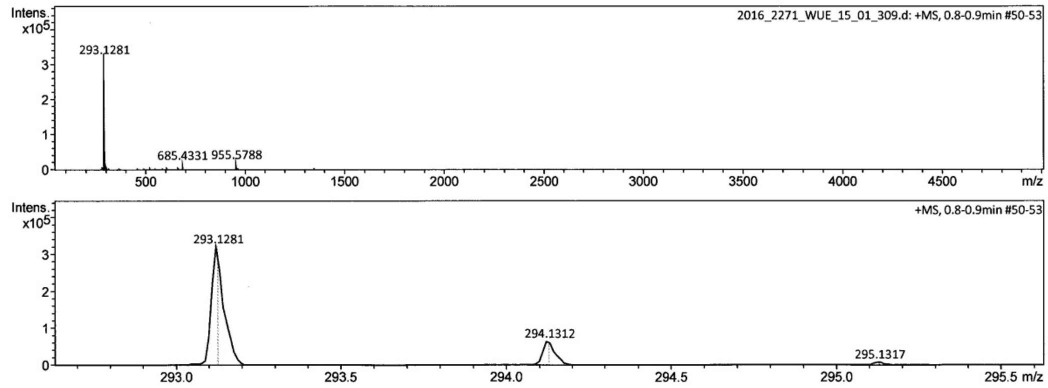

Fig. S87 HR ESI mass spectrum (MeCN/$CHCl_3$ 1:1, positive mode) of ***m*-MeO-bpb** $[M+H]^+$.

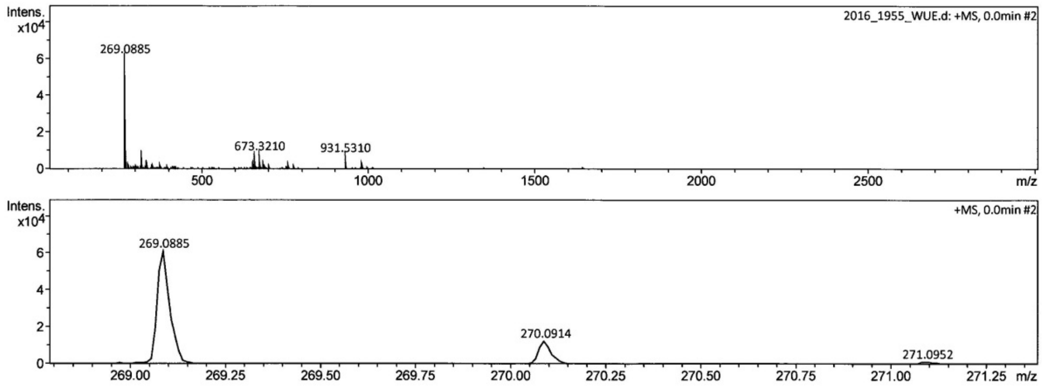

Fig. S88 HR ESI mass spectrum (MeCN/$CHCl_3$ 1:1, positive mode) of ***m*-F-bpb** $[M+H]^+$.

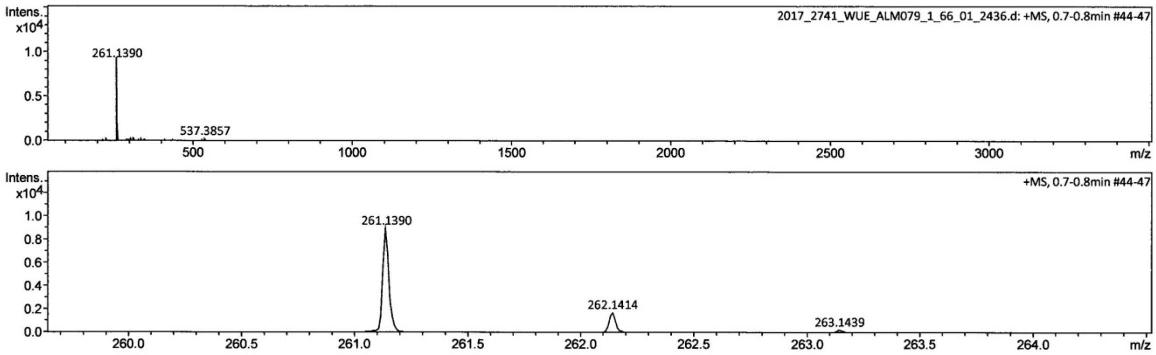

Fig. S89 HR ESI mass spectrum (MeOH/$CHCl_3$ 1:1, positive mode) of ***m*-Me-bpb** $[M+H]^+$.

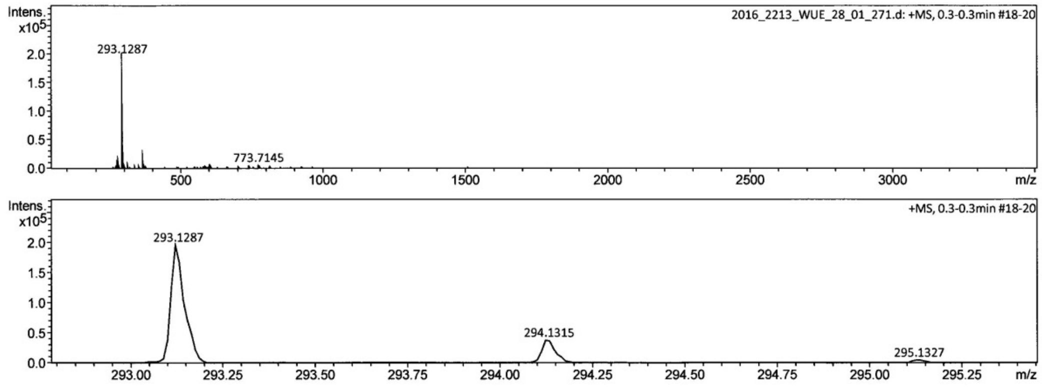

Fig. S90 HR ESI mass spectrum (MeCN/$CHCl_3$ 1:1, positive mode) of ***p*-MeO-bpb** $[M+H]^+$.

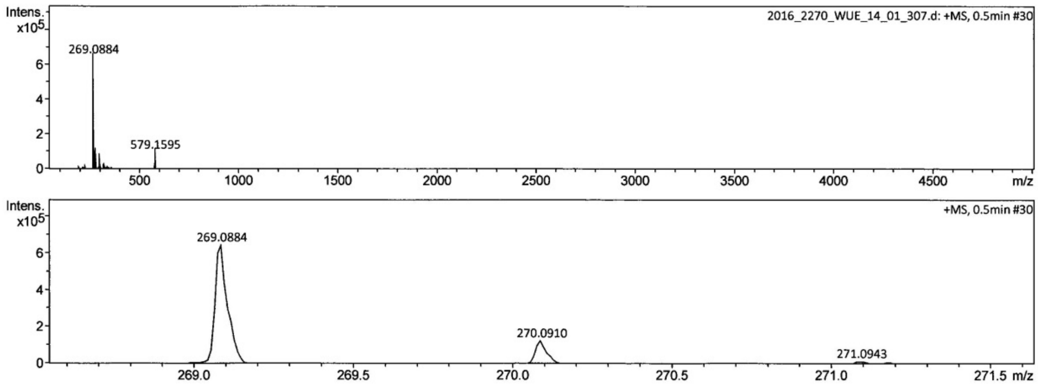

Fig. S91 HR ESI mass spectrum (MeCN/$CHCl_3$ 1:1, positive mode) of ***p*-F-bpb** $[M+H]^+$.

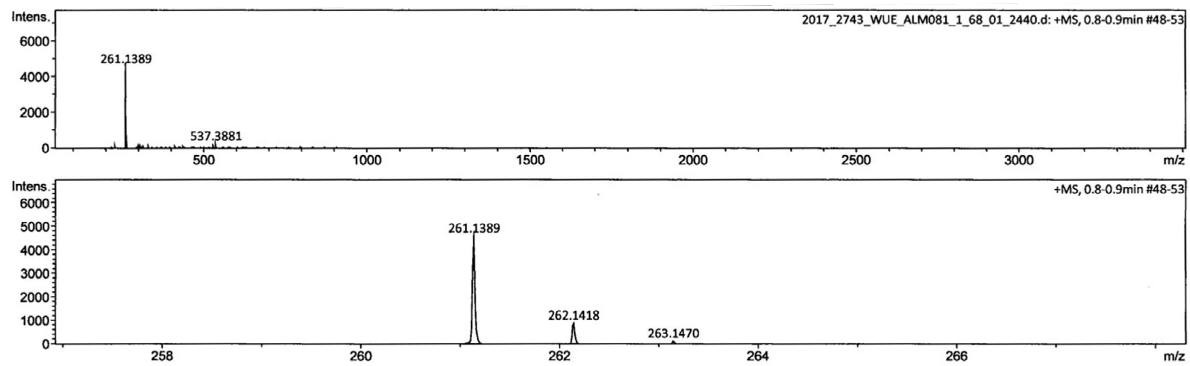

Fig. S92 HR ESI mass spectrum (MeOH/$CHCl_3$ 1:1, positive mode) of ***p*-Me-bpb** $[M+H]^+$.

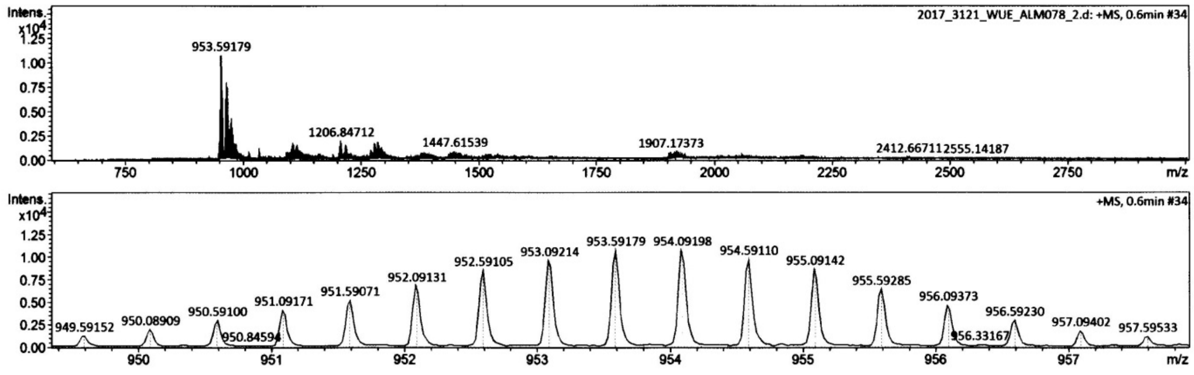

Fig. S93 HR ESI mass spectrum (MeOH/$CHCl_3$ 1:1, positive mode) of ***m*-MeO-MC3** $[M]^{2+}$.

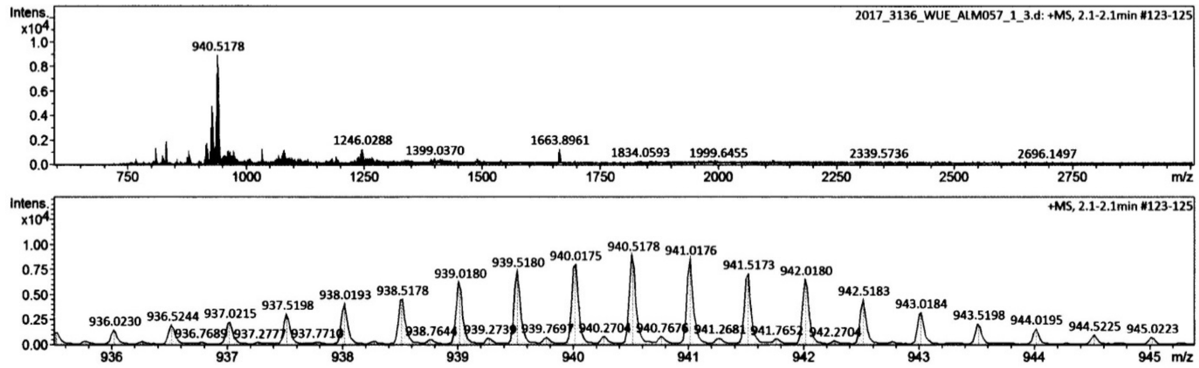

Fig. S94 HR ESI mass spectrum (MeOH/$CHCl_3$ 1:1, positive mode) of ***m*-F-MC3** $[M+2Na]^{2+}$.

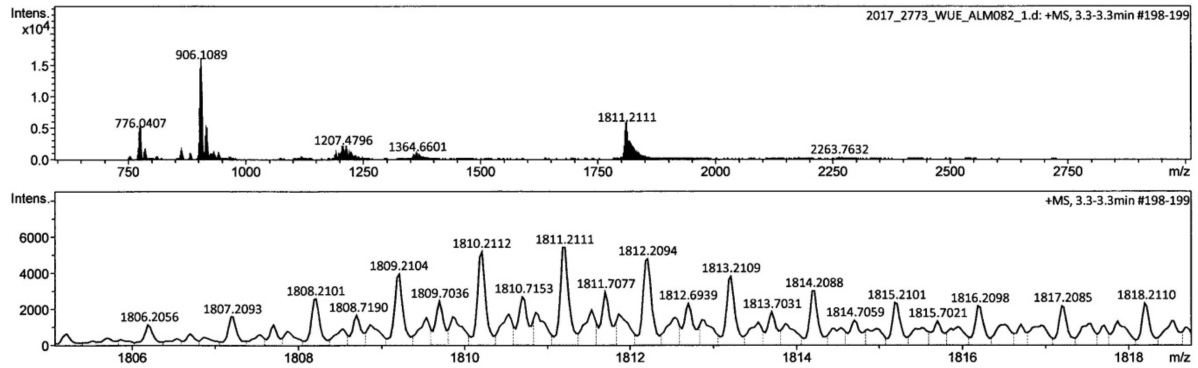

Fig. S95 HR ESI mass spectrum (MeOH/$CHCl_3$ 1:1, positive mode) of ***m*-Me-MC3** $[M]^{+}$.

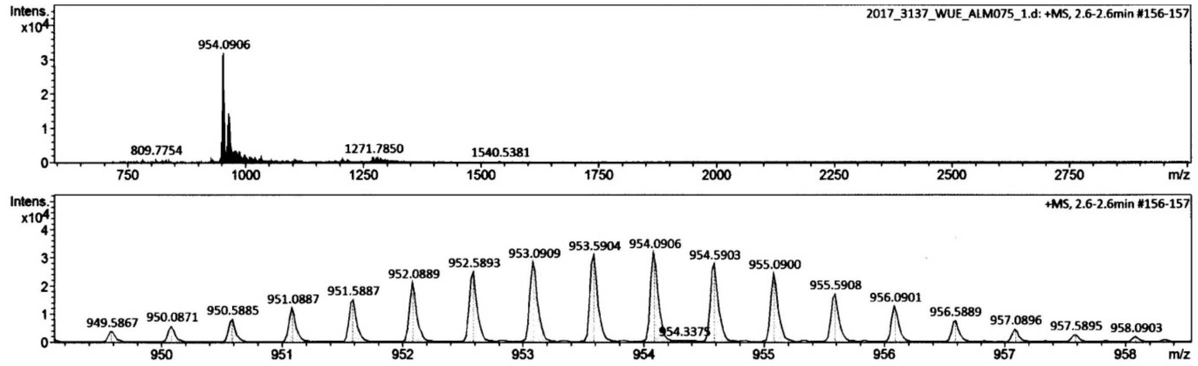

Fig. S96 HR ESI mass spectrum (MeOH/$CHCl_3$ 1:1, positive mode) of ***p*-MeO-MC3** $[M]^{2+}$.

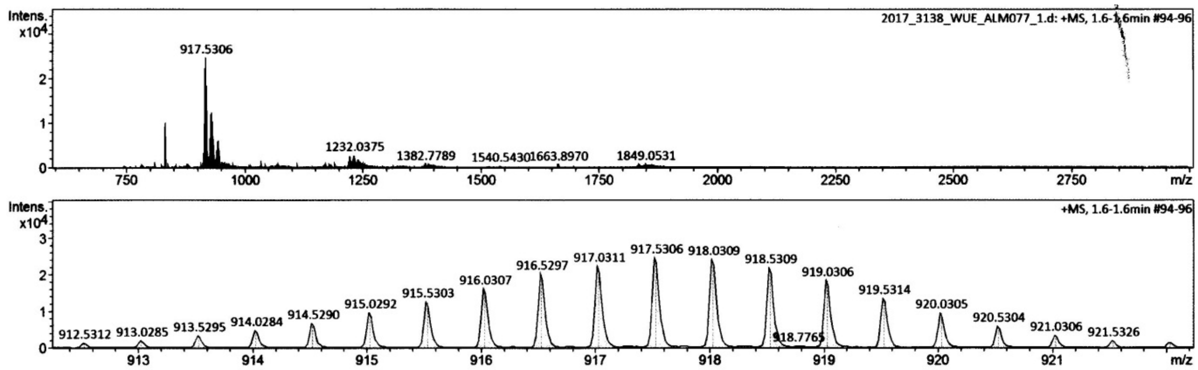

Fig. S97 HR ESI mass spectrum (MeOH/$CHCl_3$ 1:1, positive mode) of ***p*-F-MC3** $[M]^{2+}$.

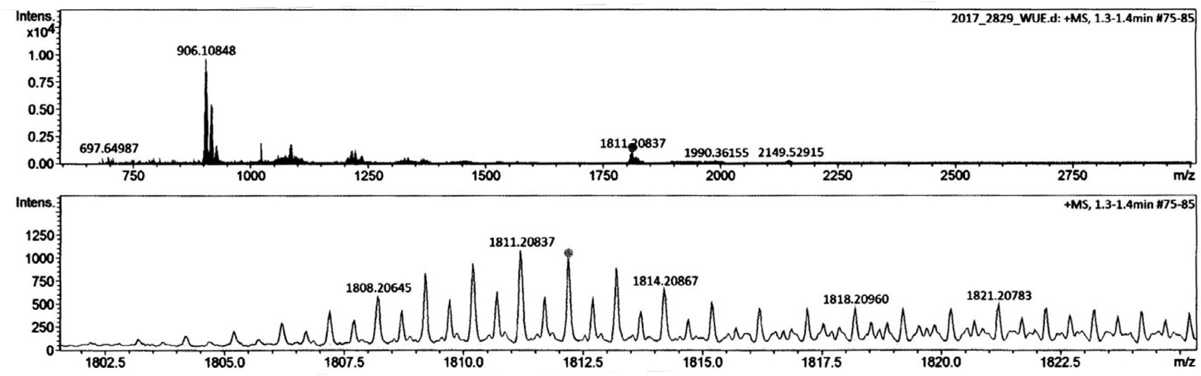

Fig. S98 HR ESI mass spectrum (MeOH/DCM 1:1, positive mode) of ***p*-Me-MC3** [M]$^+$.

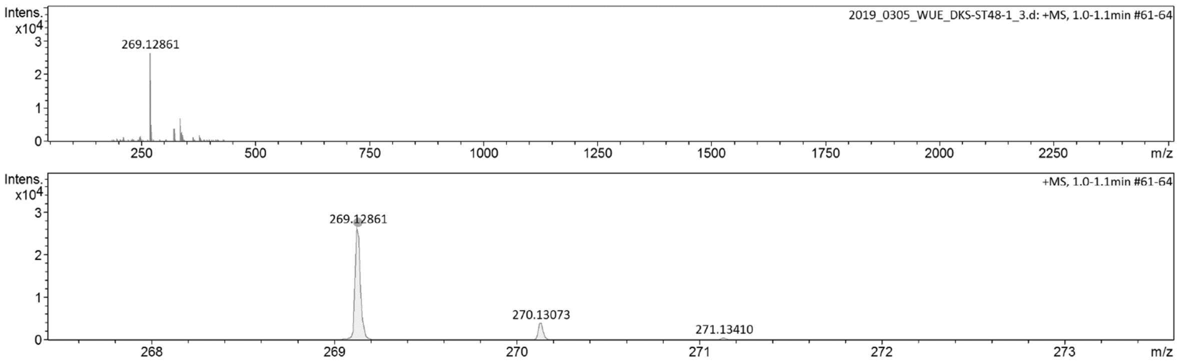

Fig. S99 HR ESI mass spectrum (MeOH/CHCl$_3$ 1:1, positive mode) of compound **6** [M+H]$^+$.

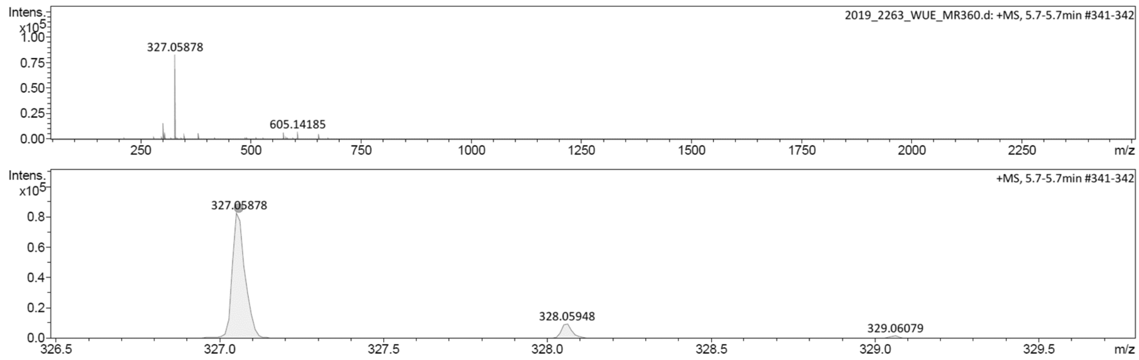

Fig. S100 HR ESI mass spectrum (MeOH/CHCl$_3$ 1:1, positive mode) of compound **7** [M+Na]$^+$.

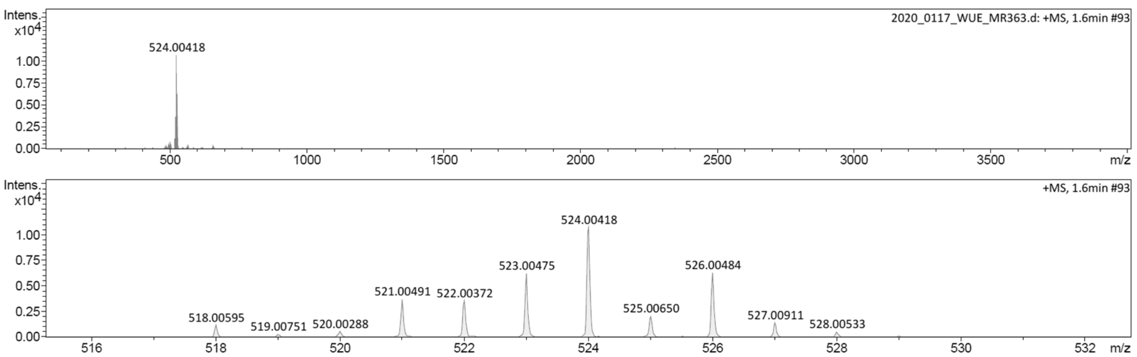

Fig. S101 HR ESI mass spectrum (MeCN/H$_2$O 1:1, positive mode) of compound **8** [M-dmso+MeCN+H]$^+$.

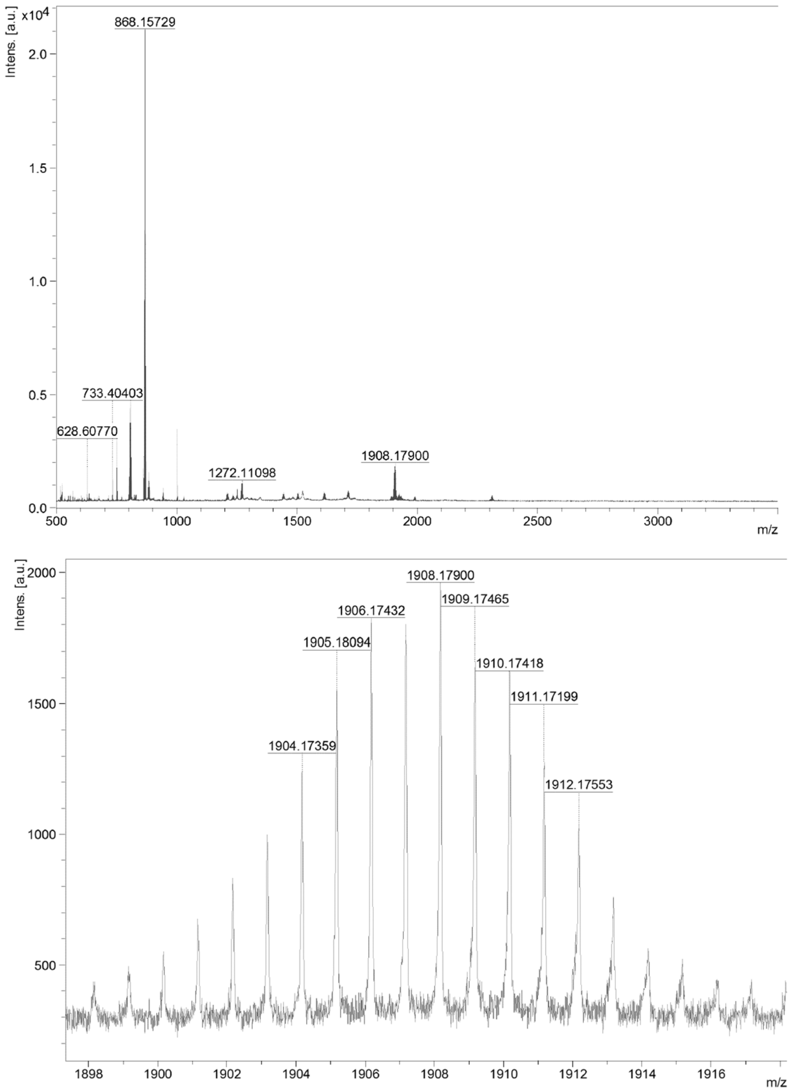

Fig. S102 HR MALDI mass spectrum (MeOH/$CHCl_3$ 1:1, positive mode) of **MeO-bda-MC3** $[M]^+$.

References

1. C. L. Donnici, D. H. Máximo-Filho, L. L. Cruz-Moreira, G. Teixeira-dos-Reis, E. Santos-Cordeiro, I. M. Ferreira-de-Oliveira, S. Carvalho and E. B. Paniago, *J. Braz. Chem. Soc.*, 1998, **9**, 455-460.
2. F. H. Burstall, *J. Chem. Soc.*, 1938, 1662-1672.
3. I. P. Evans, A. Spencer and G. Wilkinson, *J. Chem. Soc. Dalton Trans.*, 1973, 204-209.
4. E. Alessio, G. Balducci, M. Calligaris, G. Costa, W. M. Attia and G. Mestroni, *Inorg. Chem.*, 1991, **30**, 609-618.
5. E. Dulière, M. Devillers and J. Marchand-Brynaert, *Organometallics*, 2003, **22**, 804-811.
6. F. Li, B. Zhang, X. Li, Y. Jiang, L. Chen, Y. Li and L. Sun, *Angew. Chem. Int. Ed.*, 2011, **50**, 12276-12279.
7. Y. Gao, X. Ding, J. Liu, L. Wang, Z. Lu, L. Li and L. Sun, *J. Am. Chem. Soc.*, 2013, **135**, 4219-4222.
8. U. Neumann and F. Vögtle, *Chem. Ber.*, 1989, **122**, 589-591.
9. A. S. Wagman and H. E. Moser, 2010, Patent WO2010030811, Carbacephem β-lactam antibiotics.

10. D. O. Kirsanov, N. E. Borisova, M. D. Reshetova, A. V. Ivanov, L. A. Korotkov, I. I. Eliseev, M. Y. Alyapyshev, I. G. Spiridonov, A. V. Legin, Y. G. Vlasov and V. A. Babain, *Russ. Chem. Bull.*, 2012, **61**, 881-890.
11. M. Schulze, V. Kunz, P. D. Frischmann and F. Würthner, *Nat. Chem.*, 2016, **8**, 576-583.
12. G. R. Fulmer, A. J. M. Miller, N. H. Sherden, H. E. Gottlieb, A. Nudelman, B. M. Stoltz, J. E. Bercaw and K. I. Goldberg, *Organometallics*, 2010, **29**, 2176-2179.
13. G. Sheldrick, *Acta Crystallogr. A*, 2008, **64**, 112-122.
14. A. Spek, *Acta Crystallogr. A*, 1990, **46**, c34.
15. V. V. Pavlishchuk and A. W. Addison, *Inorg. Chim. Acta*, 2000, **298**, 97-102.
16. S. Gawęda, G. Stochel and K. Szaciłowski, *J. Phys. Chem. C*, 2008, **112**, 19131-19141.
17. J. J. P. Stewart, *J. Mol. Model.*, 2007, **13**, 1173-1213.
18. MOPAC2016, Version: 17.279L, J. J. P. Stewart, Stewart Computational Chemistry, web: http://OpenMopac.net.
19. W. C. Swope, H. C. Andersen, P. H. Berens and K. R. Wilson, *J. Chem. Phys.*, 1982, **76**, 637-649.
20. G. Bussi, D. Donadio and M. Parrinello, *J. Chem. Phys.*, 2007, **126**, 014101.
21. J. O. Lindner, K. Sultangaleeva, M. I. S. Röhr and R. Mitrić, *J. Chem. Theory Comput.*, 2019, **15**, 3450-3460.
22. TURBOMOLE V.7.0, 2015, a development of the University of Karlsruhe and Forschungszentrum Karlsruhe GmbH, 1989-2007, TURBOMOLE GmbH, since 2007, available from http://www.turbomole.com
23. J. P. Perdew, K. Burke and M. Ernzerhof, *Phys. Rev. Lett.*, 1996, **77**, 3865-3868.
24. F. Weigend and R. Ahlrichs, *Phys. Chem. Chem. Phys.*, 2005, **7**, 3297-3305.
25. D. Andrae, U. Häußermann, M. Dolg, H. Stoll and H. Preuß, *Theor. Chim. Acta*, 1990, **77**, 123-141.
26. A. Klamt and G. Schüürmann, *J. Chem. Soc. Perkin Trans. 2*, 1993, 799-805.
27. M. Jiang, H. Yang and H. Fu, *Organic Lett.*, 2016, **18**, 5248-5251.
28. L. Wang, L. Duan, B. Stewart, M. Pu, J. Liu, T. Privalov and L. Sun, *J. Am. Chem. Soc.*, 2012, **134**, 18868-18880.
29. L. Duan, Y. Xu, P. Zhang, M. Wang and L. Sun, *Inorg. Chem.*, 2010, **49**, 209-215.
30. L. Duan, F. Bozoglian, S. Mandal, B. Stewart, T. Privalov, A. Llobet and L. Sun, *Nat. Chem.*, 2012, **4**, 418-423.
31. L. Wang, D. W. Shaffer, G. F. Manbeck, D. E. Polyansky and J. J. Concepcion, *ACS Catal.*, 2020, **10**, 580-585.
32. Y. Jiang, F. Li, B. Zhang, X. Li, X. Wang, F. Huang and L. Sun, *Angew. Chem. Int. Ed.*, 2013, **52**, 3398-3401.
33. F. Li, C. Xu, X. Wang, Y. Wang, J. Du and L. Sun, *Chin. J. Catal.*, 2018, **39**, 446-452.
34. S. Neudeck, S. Maji, I. López, S. Meyer, F. Meyer and A. Llobet, *J. Am. Chem. Soc.*, 2014, **136**, 24-27.
35. S. Berardi, L. Francàs, S. Neudeck, S. Maji, J. Benet-Buchholz, F. Meyer and A. Llobet, *ChemSusChem*, 2015, **8**, 3688-3696.
36. L. Francàs, R. Matheu, E. Pastor, A. Reynal, S. Berardi, X. Sala, A. Llobet and J. R. Durrant, *ACS Catal.*, 2017, **7**, 5142-5150.
37. H.-W. Tseng, R. Zong, J. T. Muckerman and R. Thummel, *Inorg. Chem.*, 2008, **47**, 11763-11773.
38. A. Lewandowska-Andralojc, D. E. Polyansky, R. Zong, R. P. Thummel and E. Fujita, *Phys. Chem. Chem. Phys.*, 2013, **15**, 14058-14068.
39. L. Vigara, M. Z. Ertem, N. Planas, F. Bozoglian, N. Leidel, H. Dau, M. Haumann, L. Gagliardi, C. J. Cramer and A. Llobet, *Chem. Sci.*, 2012, **3**, 2576-2586.
40. D. J. Wasylenko, C. Ganesamoorthy, B. D. Koivisto, M. A. Henderson and C. P. Berlinguette, *Inorg. Chem.*, 2010, **49**, 2202-2209.
